# Surfaceome and Exoproteome Dynamics in Dual-Species *Pseudomonas aeruginosa* and *Staphylococcus aureus* Biofilms

**DOI:** 10.3389/fmicb.2021.672975

**Published:** 2021-06-25

**Authors:** Inés Reigada, Paola San-Martin-Galindo, Shella Gilbert-Girard, Jacopo Chiaro, Vincenzo Cerullo, Kirsi Savijoki, Tuula A. Nyman, Adyary Fallarero, Ilkka Miettinen

**Affiliations:** ^1^Drug Research Program, Division of Pharmaceutical Biosciences, Faculty of Pharmacy, University of Helsinki, Helsinki, Finland; ^2^Department of Immunology, Institute of Clinical Medicine, Rikshospitalet, University of Oslo, Oslo, Norway

**Keywords:** *Staphylococcus aureus*, *Pseudomonas aeruginosa*, dual-species biofilm, proteomics, exoproteome, surfaceome

## Abstract

Bacterial biofilms are an important underlying cause for chronic infections. By switching into the biofilm state, bacteria can evade host defenses and withstand antibiotic chemotherapy. Despite the fact that biofilms at clinical and environmental settings are mostly composed of multiple microbial species, biofilm research has largely been focused on single-species biofilms. In this study, we investigated the interaction between two clinically relevant bacterial pathogens (*Staphylococcus aureus* and *Pseudomonas aeruginosa*) by label-free quantitative proteomics focusing on proteins associated with the bacterial cell surfaces (surfaceome) and proteins exported/released to the extracellular space (exoproteome). The changes observed in the surfaceome and exoproteome of *P. aeruginosa* pointed toward higher motility and lower pigment production when co-cultured with *S. aureus*. In *S. aureus*, lower abundances of proteins related to cell wall biosynthesis and cell division, suggesting increased persistence, were observed in the dual-species biofilm. Complementary phenotypic analyses confirmed the higher motility and the lower pigment production in *P. aeruginosa* when co-cultured with *S. aureus.* Higher antimicrobial tolerance associated with the co-culture setting was additionally observed in both species. To the best of our knowledge, this study is among the first systematic explorations providing insights into the dynamics of both the surfaceome and exoproteome of *S. aureus* and *P. aeruginosa* dual-species biofilms.

## Introduction

Biofilms are defined as communities of microbial cells encased within a self-produced matrix that adhere to biological or non-biological surfaces (Hall-Stoodley et al., 2004; Paharik and Horswill, 2016). Biofilm formation offers microorganisms with protection from a wide range of environmental challenges, such as UV exposure, metal toxicity, acid exposure, dehydration, and salinity (Hall-Stoodley et al., 2004). Biofilms can be beneficial, for example, in the case of biofilms involved in the maintenance of a healthy human microbiota, but they often cause major problems as well (Yan and Bassler, 2019). In clinical settings, they are an important underlying cause of chronic infections. Switching into the biofilm state helps pathogenic bacteria to evade host defenses and withstand antibiotic chemotherapy (Costerton et al., 1999), which makes this growth mode regarded as the most important non-specific mechanism of antimicrobial tolerance (Kumar et al., 2017; Singh et al., 2017). In addition, conditions boosting the development of dormant, non-dividing cells (persisters) during biofilm formation are also of high relevance, as such phenotypic variants are associated with multidrug tolerance (Lewis, 2007), thereby further complicating the treatment of the infectious disease.

Typically, biofilm research has focused on single bacterial species, but in clinical and environmental conditions, biofilms are mostly composed of multiple species (Røder et al., 2016). The co-existence of different species within a biofilm can provide the inhabitants with numerous benefits, such as the availability of compounds synthesized by the cohabiting bacteria or higher evasion of the host defenses (Yang et al., 2011; Nguyen and Oglesby-Sherrouse, 2016). However, the competition between different co-existing species may also be ferocious, and microbes display numerous techniques for subverting each other (Elias and Banin, 2012; Chew et al., 2018). Either way, interspecies interaction often results in higher virulence and increased tolerance to antibiotic therapy (Rice et al., 2016; Thet et al., 2019). *Staphylococcus aureus* and *Pseudomonas aeruginosa*, a Gram-positive and a Gram-negative bacterial species, respectively, are opportunistic pathogens that have been shown to co-exist in biofilms related to numerous infections (Yang et al., 2011; Nair et al., 2014; Tande and Patel, 2014; Limoli et al., 2016). In cystic fibrosis (CF) patients, lung colonization by *S. aureus* (a normal component of the upper airway microbiome) facilitates *P. aeruginosa* colonization which, in turn, is strongly linked to worse disease outcomes (Lyczak et al., 2002). Although the interaction between these two species is competitive and especially deleterious to *S. aureus*, both partially benefit from it. In clinical isolates from patients in whom tobramycin failed to eradicate *P. aeruginosa*, it was observed that the interaction between a *S. aureus* surface protein and an exopolysaccharide component of *P. aeruginosa* was essential to shaping the architecture of *P. aeruginosa* biofilms, resulting in an even higher tolerance to tobramycin (Armbruster et al., 2016; Beaudoin et al., 2017). Additionally, the exoproteins of *S. aureus* have been shown to induce small-colony variants in *P. aeruginosa*, which increases the aminoglycoside tolerance of the pathogen (Michelsen et al., 2014). In an analogous manner, the production of 4-hydroxy-2-heptylquinoline-*N*-oxide by *P. aeruginosa* induces small-colony variants in *S. aureus*, making it more tolerant to tobramycin and vancomycin, as well as facilitating *S. aureus* immune evasion (Hoffman et al., 2006; Mitchell et al., 2010; Orazi and O’toole, 2017).

Another pathological condition where *S. aureus* and *P. aeruginosa* are the two most prevalent pathogens is chronic wounds (Serra et al., 2015). Similar to what has been observed in CF, *S. aureus* facilitates the attachment and subsequent biofilm formation by *P. aeruginosa*. Thus, the co-existence of these two bacteria increases their virulence and tolerance, making them more resilient to antibiotics (Deleon et al., 2014; Alves et al., 2018). The “omics-based” technologies (*e.g*., genomics, transcriptomics, and proteomics) present a valuable tool for studying such interactions in biofilms (He and Chiu, 2003; Azevedo et al., 2009). The analysis of proteins present in the extracellular milieu, including secreted proteins or proteins present in extracellular vesicles (exoproteomics), can help in identifying key targets for inhibiting virulence and adaptation to antibiotics (Guendouze et al., 2017), and it also provides information on the interspecies interactions and infectious processes (Chagnot et al., 2013). On the other hand, the analysis of proteins associated with the cell surface (surfaceomics) complements these analyses, as these proteins form the first line of molecular interaction with the host and the environment. Although a number of proteomics analyses have been carried out on multispecies biofilms (Herschend et al., 2017; Pallett et al., 2019), there is still a lack of extensive proteomics research of clinically relevant mixed-species biofilms, particularly in the case of *S. aureus* and *P. aeruginosa.*

In the present study, we established a dual-species biofilm model community for *S. aureus* and *P. aeruginosa* and used label-free quantitative proteomics for investigating the intra- and interspecies interactions. We focused on the surfaceome and the exoproteome in the co-cultured biofilms, and we compared their corresponding proteomes with those identified from single-species biofilms. Finally, the most interesting proteomic findings were further investigated by phenotypic assays.

## Materials and Methods

### Bacterial Strains and Growth Media

Clinical bacterial strains of *S. aureus* ATCC 25923 and *P. aeruginosa* PAO1 were acquired from the HAMBI mBRC—Microbial Domain Biological Resource Centre at the University of Helsinki (Helsinki, Finland). *S. aureus* ATCC 25923 was routinely maintained on tryptic soy agar plates (Lab M, Lancashire, United Kingdom), whereas *P. aeruginosa* PAO1 was maintained on Miller’s lysogeny broth (LB) agar (Fisher BioReagents, Pittsburgh, PA, United States). The suspension cultures of both species were prepared in Miller’s LB medium (VWR Chemicals, Radnor, PA, United States) at 37°C and aerated at 220 rpm. Selective nutrient agars, mannitol salt agar (MSA; Lab M, Lancashire, United Kingdom) and cetrimide agar supplemented with 10 ml/l molecular-biology-grade glycerol (Sigma-Aldrich, St. Louis, MO, United States), were also used for culturing *S. aureus* and *P. aeruginosa* strains.

### Single- and Dual-Species Biofilm Growth

**FIGURE 1:**
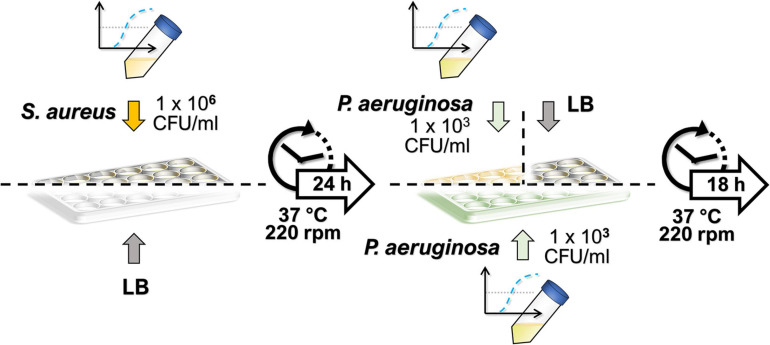
Culturing protocol of dual-species biofilms. *Staphylococcus aureus* biofilms were allowed to develop for 24 h, after which a low-concentration inoculum of *Pseudomonas aeruginosa* was added, and the dual-species biofilm was incubated under the same culture conditions for 18 h. Single-species biofilms were obtained by adding sterile lysogeny broth in place of the second species during culturing.

The culturing protocol used for the dual-species biofilms is depicted in Figure 1. In the dual-species biofilm growth, *S. aureus* biofilms were allowed to grow first, prior to the addition of *P. aeruginosa*, to facilitate *S. aureus* survival in the co-culture conditions. Thus, *S. aureus* colonies from an overnight-grown streak plate were inoculated into 5 ml of LB in duplicate until reaching a bacterial concentration of approximately 10^8^ colony-forming units (CFU)/ml. Mid-exponential cultures were diluted to approximately 1 × 10^6^ CFU/ml in LB, and *S. aureus* suspension was seeded onto 24-well plates (Nunc^TM^, Thermo Fisher Scientific, Waltham, MA, United States) at 1.5 ml/well. In place of *S. aureus*, 1.5 ml/well of sterile LB was used in wells intended for *P. aeruginosa* monocultures. The biofilms of *S. aureus* were allowed to mature for 24 h (37°C, 220 rpm), after which the planktonic suspensions were discarded, and 1.5 ml/well of *P. aeruginosa* suspension was added both onto *S. aureus* biofilms and to LB-conditioned wells (for obtaining the single-species biofilm of *P. aeruginosa*). Similarly, *P. aeruginosa* suspensions were obtained by inoculating a colony from an overnight grown streak plate into 5 ml of LB in duplicate. On this occasion, the mid-exponential cultures were diluted to approximately 1 × 10^3^ CFU/ml. To obtain the *S. aureus* monocultures, 1.5 ml of sterile LB was added per well in place of *P. aeruginosa*. After the biofilms were further incubated for 18 h (37°C, 220 rpm), the planktonic solutions of both species were removed, and the wells were washed with 1.5 ml of LB. The biofilms were then re-suspended in 1 ml of sterile phosphate-buffered saline (PBS; Lonza, Basel, Switzerland) by vigorously scraping with a 1-ml tip while pipetting up and down. The harvested biofilms were vortexed for 10 s and further sonicated in a water bath in Ultrasonic Cleaner 3800 (Branson Ultrasonics, Danbury, CT, United States) at 25°C and 35 kHz for 5 min to disrupt possible microaggregates. The samples were serially diluted in sterile LB and plated onto cetrimide or MSA for the selective culture of *P. aeruginosa* or *S. aureus*, respectively. The number of viable colonies was calculated by counting them after an overnight incubation.

In order to monitor the bacterial density of *S. aureus* ATCC 25923 and *P. aeruginosa* PAO1 in the mono- and co-cultured biofilms, we quantified the number of viable cells in the developing biofilms at different time points (4, 8, 12, 16, and 18 h) during the 18 h of incubation as described above.

### Flow Cytometry Analysis

The flow cytometry analysis was performed using a BD Accuri 6 plus (BD Biosciences, Franklin Lakes, NJ, United States). The biofilms were harvested as described in Section “Single- and Dual-Species Biofilm Growth” and were washed twice with PBS (2 × 3 min, 7,000 *g*, 4°C). The remaining cells were stained with 0.1 μM of SYTO 9 (Invitrogen^TM^, Thermo Fisher Scientific, Waltham, MA, United States) and 20 μM of propidium iodide (PI) (Invitrogen^TM^, Thermo Fisher Scientific, Waltham, MA, United States) for 15 min at room temperature (RT). SYTO 9 was used to stain viable cells, while PI was utilized to mark non-viable cells, as its binding to DNA is excluded from cells with an intact plasma membrane. After incubation, two washing steps were performed with PBS (2 × 3 min, 7,000 *g*, 4°C).

### Fluorescence Microscopy

The single- and dual-species biofilms were visualized using two fluorescent nucleic-acid-binding dyes, hexidium iodide (HI), and SYTO 9 (Invitrogen^TM^, Thermo Fisher Scientific, Waltham, MA, United States). The dye HI is claimed to preferably stain Gram-positive bacteria by displaying a red fluorescence, while SYTO 9 stains both Gram-positive and Gram-negative bacteria and has a green fluorescence (Mason et al., 1998). The stock solution of HI was prepared by dissolving HI (5 mg) in 1 ml of dimethyl sulfoxide (DMSO) and further diluted to obtain a concentration of 4.67 mM. The stain SYTO 9 was provided by the manufacturer at a concentration of 3.34 mM in DMSO. The working solution of the dye mixture was obtained by combining the stocks (3 μl of SYTO 9 and 2 μl of HI) in 1 ml of sterile water.

After the biofilms were formed as indicated in Section “Single- and Dual-Species Biofilm Growth,” they were washed once using PBS, followed by the addition of 70 μl of the working solution of the dye mixture. The samples were kept in the darkness, at RT, and after 15 min the unbound dye was removed, and images were acquired using an Invitrogen^TM^ EVOS^®^ FL Imaging System (Thermo Fisher Scientific, Waltham, MA, United States). The filters for the green fluorescent protein (λ_*excitation*_ = 470/22 nm; λ_*emission*_ = 510/42 nm) and red fluorescent protein (λ_*ex*__*citation*_ = 531/40 nm; λ_*emission*_ = 593/40 nm) with a 60× objective were used. The representative images of the biofilms were taken in duplicate, and images were recorded using both filters. The overlapping of green and red fluorescence was made directly on the microscope using the multiple-channel function.

### Protein Extraction and Purification

Single- and dual-species biofilms were obtained as indicated above using individually inoculated biological triplicates. The protein extraction process was performed on ice. Culture supernatants (two technical replicates for one biological replica) were separated from biofilms (four technical replicates for one biological replica) for the exoproteome analyses, while the remaining biofilms were dispersed by vigorous scraping using 1 ml of 100 mM Tris-HCl, pH 6.8, with a 1-ml tip while pipetting up and down. The washed cells were collected by centrifugation (3 min, 8,000 *g*, 4°C) for the surfaceome analyses.

#### Exoproteome Extraction

The culture supernatants containing non-adherent planktonic bacteria from the dual-species biofilms were pelleted by centrifugation (3 min, 8,000 *g*, 4°C), and the supernatants were sterile-filtered through a 0.22-μm Millex-GV PVDF filter (13 mm) (Merck, Darmstadt, Germany). Aliquots (1,000 μl) of the filtrates were precipitated with 4% trichloroacetic acid (TCA) for 45 min on ice. The proteins were precipitated by centrifugation (21,000 *g*, 30 min, 4°C), and the precipitates were washed twice with 1 ml ice-cold acetone (21,000 *g*, 15 min, 4°C) and dried by a short evaporation at 57°C. Then, proteins were solubilized in 0.1% RapiGest SF (Waters, Milford, MA, United States) and reconstituted in 100 mM triethylammonium bicarbonate (TEAB) buffer, pH 8.5 (Thermo Fisher Scientific, Waltham, MA, United States). For the protein content estimation, direct absorbance measurements were done at 280 nm using a μDrop Plate on a Multiskan Sky Microplate Spectrophotometer (Thermo Fisher Scientific, Waltham, MA, United States). The samples were reduced by 10 mM DL-dithiothreitol (DTT; Sigma-Aldrich, Saint Louis, MO, United States) for 45 min at 60°C and alkylated by 15 mM iodoacetamide (Sigma-Aldrich, Saint Louis, MO, United States) for 60 min at RT and protected from light. The extra-iodoacetamide was quenched with an additional 20 mM DTT. Aliquots corresponding to 15 μg of protein were taken, and the volume was adjusted to 90 μl with 100 mM TEAB. Sequence-grade modified trypsin (Promega, Madison, WI, United States) was reconstituted in the provided buffer solution and added at a ratio of 1:50 enzyme-to-protein in a final volume of 100 μl. The samples were digested for 20 h at 37°C, after which the reaction was stopped by acidification with 0.6% trifluoroacetic acid (TFA; Merck, Darmstadt, Germany). Finally, the samples were centrifuged (10 min, 18,000 *g*), and the supernatants were collected for further analysis.

#### Surfaceome Extraction

Trypsin shaving was carried out as described previously for *S. aureus* biofilms (Hiltunen et al., 2019). The dispersed biofilms were pelleted (2 × 3 min, 7,000 *g*, 4°C) and resuspended in 100 mM TEAB. Sequence-grade modified trypsin was added at 55 ng/μl into a final volume of 100 μl, and the samples were incubated for 20 min at 37°C. The digested samples were pelleted (3 min, 7,000 *g*, 4°C), and the supernatants were transferred to Costar Spin-X 0.22-μm filters (Corning Inc., Corning, NY, United States) and centrifuged for 2 min at 16,000 *g*. The filtrates were further incubated for 20 h at 37°C to finalize the digestion. The protein content was estimated by direct absorbance measurements at 280 nm using a μDrop Plate on a Multiskan Sky Microplate Spectrophotometer, and the digestions were stopped by acidification with 0.6% TFA.

The trypsin shaving protocol used here was shown to be suitable for both *S. aureus* and *P. aeruginosa* by carrying out trypsinization at 55 ng/μl for 20 min on their corresponding biofilms, which were recovered as described above. The digested samples were washed once in PBS (3 min, 7,000 *g*), serially diluted in PBS, and plated on LB agar. After an overnight incubation, the viable colonies were counted. No viability loss was detected in the trypsinized samples as compared to the non-trypsinized bacteria (Supplementary Figures 1A,B). In the case of *S. aureus*, in order to avoid the misinterpretation of *S. aureus* viability due to the break-up of clusters, the integrity of the cell membrane was further confirmed by flow cytometry (Supplementary Figures 1C,D).

Finally, all the surfaceome and exoproteome samples were purified and concentrated using the ZipTip C18 system (Merck, Darmstadt, Germany) and dried.

### Protein Identification and Quantification

The liquid chromatography–tandem mass spectrometry (LC–MS/MS) analysis of exoproteome and surfaceome samples was carried out similarly for the exoproteome and surfaceome samples as described previously (Lorey et al., 2017; Savijoki et al., 2020). Briefly, samples were dissolved in 0.1% (v/v) formic acid, and equal volumes of the concentrated and purified tryptic peptides were loaded to an Easy-nLC 1000 nano-LC system (Thermo Fisher Scientific, Waltham, MA, United States), coupled with an Orbitrap QExactive Plus^TM^ mass spectrometer (Thermo Fisher Scientific, Waltham, MA, United States) equipped with a nanoelectrospray ion source (Easy-Spray^TM^, Thermo Fisher Scientific, Waltham, MA, United States). The LC separation was performed using a C18 column (25-cm bed length, 2-μm beads, 100 Å, 75-μm inner diameter) with a flow rate of 300 nl/min. The peptides were eluted with 2–30% solvent gradient (100% acetonitrile/0.1% formic acid) over 60 min. The MS was operated in the data-dependent acquisition mode, with the 10 most abundant multiple-charged ions selected for fragmentation. The obtained MS raw files were submitted to MaxQuant software (v.1.6.1.0) for protein identification and label-free quantification using a database composed of *S. aureus* ATCC 25923 and *P. aeruginosa* PAO1 protein sequences in forward and reverse. Matching between runs was omitted. The mass tolerances of 20 and 4.5 ppm were applied for the first and main search, respectively. Trypsin digestion without proline restriction option was applied, with two missed cleavages allowed. The minimal unique + razor peptide number was set to one, and false discovery rate (FDR) was set to 0.01 for peptide and protein identification. The obtained mass spectrometry proteomics data have been deposited to the ProteomeXchange Consortium *via* the PRIDE (Perez-Riverol et al., 2019) partner repository with the dataset identifier PXD02344.

### Follow-Up Studies

#### Differences in Pigmentation

The *P. aeruginosa* PAO1 monocultures and the dual-species biofilms were grown in 24-well plates as described in Section “Single- and Dual-Species Biofilm Growth,” after which the samples were centrifuged for 15 min (10,000 *g*, 4°C) and the supernatants were collected for further analysis. For the pyoverdine measurement, a fluorescent-based detection method was used as in Elliott (1958). Briefly, 200-μl samples of the supernatants were carefully transferred to a 96-well plate (Thermo Fisher Scientific^TM^ Nunc 96 Well Black/Clear Bottom Plate, TC Surface), and the top fluorescence was measured (λ_*excitation*_ = 360 nm, λ_*emission*_ = 474 nm) in a Varioskan LUX Multimode microplate reader (Thermo Fisher Scientific, Waltham, MA, United States). For the pyocyanin quantification, the method described by Essar et al. (1990); Debritto et al. (2020) was employed. The supernatants were acidified to pH < 2 with HCl (Riedel De Haen, Thermo Fisher Scientific, Waltham, MA, United States), and 100-μl samples were transferred to a 96-well plate (Nunclon^TM^Δ surface polystyrene plates, Nunc, Roskilde, Denmark) to record the absorbance at 520 nm in a Varioskan LUX Multimode microplate reader. The obtained relative florescence units in the case of pyoverdine and the absorbance units in the case of pyocyanin were normalized with the total CFU count per well of the *P. aeruginosa* biofilm formed in mono- or co-culture conditions.

#### Motility Assay

*Pseudomonas aeruginosa* PAO1 biofilms were formed as described in Section “Single- and Dual-Species Biofilm Growth.” Both the monocultured biofilms and the biofilms co-cultured with *S. aureus* ATCC 25923 were harvested in combination with their respective supernatants. This suspension was vortexed for 10 s and further sonicated in a water bath in Ultrasonic Cleaner 3800 at 25°C, 35 kHz, for 5 min to disrupt possible microaggregates. Subsequently, 50 μl of this suspension was plated on soft LB agar plates. The agar was prepared by adding nutrient agar (Sigma-Aldrich, St. Louis, MO, United States) at 0.5% concentration to LB. The added suspension was allowed to absorb and set at 37°C. The growth of the colony diameter was assessed after 8 h. To verify that the migration zone only consisted of *P. aeruginosa* and not of motile *S. aureus* (Pollitt et al., 2015), samples of four different locations of the halos were collected and plated on selective agar plates (MSA and cetrimide agar).

#### Antibiotic Susceptibility Testing

Vancomycin was chosen to compare the susceptibility of *S. aureus* biofilms in monoculture or co-cultured with *P. aeruginosa*, while polymyxin B was used to study the respective susceptibility of *P. aeruginosa* biofilms. Both reagents were purchased from Sigma-Aldrich (St. Louis, MO, United States). Polymyxin B was tested at 50, 100, and 200 μM, while vancomycin was tested at 50, 100, and 200 μM. The selection of the concentrations was based on finding one able to reduce the biofilms formed in monoculture. The stocks of the antibiotics were prepared in LB.

The biofilms were formed as described in Section “Single- and Dual-Species Biofilm Growth,” after which the planktonic solutions were removed, and the wells were washed with 1.5 ml LB. Subsequently, 1.5 ml of antibiotic solution was added to the wells and incubated for 4 h at 37°C. After the incubation, the biofilms were harvested as described in Section “Single- and Dual-Species Biofilm Growth.” The samples serially diluted with sterile LB were plated using cetrimide or MSA for the selective culture of *P. aeruginosa* or *S. aureus*, respectively. Viable colonies were counted following an overnight incubation. The results are expressed as the log_10_ reduction achieved by each concentration in comparison to the respective untreated biofilm controls in mono- or co-culture settings.

### Proteomic Data Processing and Bioinformatic Analysis

For the determination of differential expression, missing values were imputed by random draws from an adjusted normal distribution using the Perseus software, v.1.6.2.3 (Tyanova et al., 2016). At least two valid values in at least one group were required for the protein to be included in the analysis. The log_2_-transformed normalized intensities (LFQ) were used for statistical comparisons of the differential expression in Perseus using Student’s *t*-test with a permutation-based FDR adjustment, applying a FDR of 0.15 due to the small sample size and the exploratory purpose of this study (Korpela et al., 2016; Neves et al., 2017). To avoid bias arising from a markedly lower fraction of staphylococcal *vs*. *P. aeruginosa*-derived proteins in the co-culture surfaceomes, an LFQ cutoff of 1.75 × 10^8^ was applied for the *S. aureus* surfaceome dataset. Such threshold value was selected as it corresponded to the lowest monoculture quantity at which co-culture quantifications were acquired. Cross-tabulation and binary logistic regression analyses were carried out in IBM SPSS Statistics v. 25 (Armonk, NY, United States).

The isoelectric point (pI) and molecular weight (Mw) predictions were acquired from EMBOSS Pepstats (Rice et al., 2000; Chojnacki et al., 2017). The secretion modes were predicted with SignalP 5.0 (Almagro Armenteros et al., 2019) and SecretomeP 2.0 (Bendtsen et al., 2005). The grand average of hydropathy (GRAVY) indices were acquired from the GRAVY calculator by Dr. Stephan Fuchs (http://www.gravy-calculator.de). Protein interactions were studied using STRING database, v. 11 (Szklarczyk et al., 2019). The interaction scores were set to medium (0.400) or high (0.700) confidence based on the network complexity. The interacting proteins were clustered using Markov clustering (Brohée and Van Helden, 2006), with the inflation parameter set to 4.0. Functional enrichment in terms of Gene Ontology biological process (The Gene Ontology Consortium, 2017) and Kyoto Encyclopedia of Genes and Genomes (KEGG) pathway (Kanehisa and Goto, 2000; Kanehisa et al., 2019) annotations was statistically assessed in STRING (Szklarczyk et al., 2019) by both rank- and gene set-based approaches, with FDR of 0.15.

The comparison of group means for antibiotic susceptibility and pigmentation test was carried out using independent-samples Student’s *t*-tests, which were processed by GraphPad Prism, v. 8.00 software.

## Results and Discussion

### Dual-Species Biofilm Model

The *in vitro* biofilm model proposed herein allowed the formation of a considerable biofilm biomass of both *S. aureus* and *P. aeruginosa* on polystyrene 24-well plates. In the pathologies where these two pathogens have been co-isolated, for instance, in the case of cystic fibrosis, it has been observed that *S. aureus* can be outcompeted by *P. aeruginosa* and that its survival would depend on its capacity of forming small colony variants, tilting it into a more persistent phenotype (Hoffman et al., 2006). In fact, the increase on the incidence of *P. aeruginosa* has been shown to coincide with a decreasing *S. aureus* incidence in this pathology, which may indicate an antagonistic relationship between the two pathogens (Hotterbeekx et al., 2017). Since *P. aeruginosa* can promptly overtake or even eradicate *S. aureus* in co-culture conditions (Filkins et al., 2015; Woods et al., 2019), the effect of the inoculum density of *P. aeruginosa* on its biofilm formation was evaluated to find an optimal starting cell density. The inoculum density range of three magnitudes (10^3^ to 10^5^ CFU/ml) produced comparable *P. aeruginosa* monoculture biofilms after 18 h in terms of viable counts (Supplementary Figure 2). In consequence, highly diluted inocula of *P. aeruginosa* were used for further experiments to slow down the competitive advantage of *P. aeruginosa* over *S. aureus.*

**FIGURE 2:**
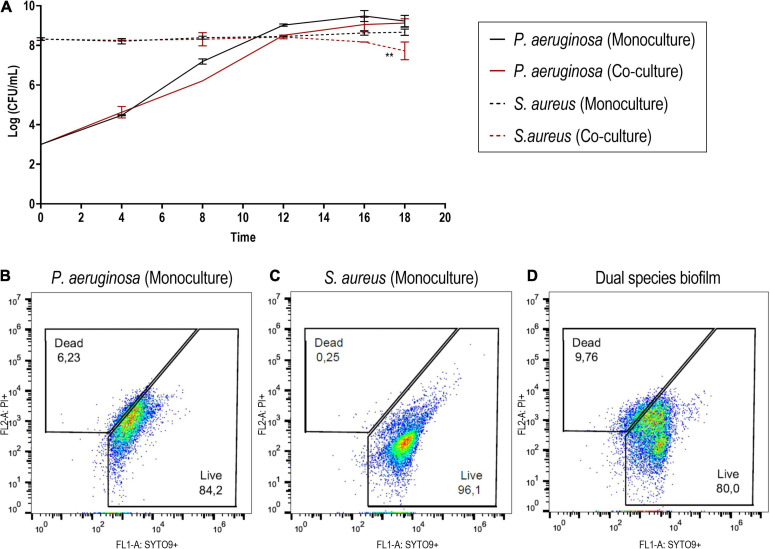
**(A)** Monitoring of the bacterial density of *Staphylococcus aureus* ATCC 25923 and *Pseudomonas aeruginosa* PAO1 in mono- and co-cultured biofilms at different time points. **(B–D)** Cytographs corresponding to cells recovered from the biofilm formed by *P. aeruginosa* **(B)** and *S. aureus* **(C)** in monoculture and the dual-species biofilm **(D)**. SYTO 9 was used to stain viable cells, while PI was utilized to stain cells with disrupted cell membrane integrity. The results are expressed as the mean of at least two biological repetitions with their ± SD. Statistical differences were assessed by comparing the mono- and co-cultured biofilms of each bacterial species (Student’s *t*-test, ^∗∗^*p* < 0.01).

**FIGURE 3:**
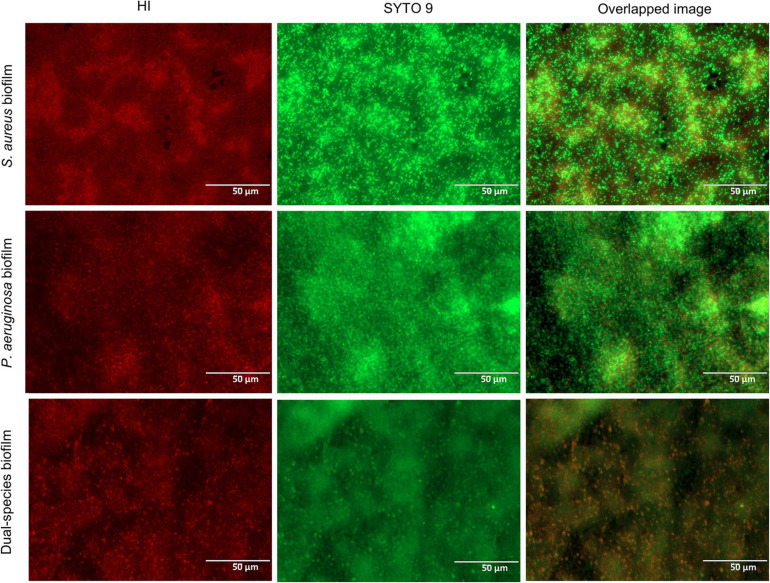
Fluorescence microscopy images of single- and dual-species biofilms stained with a dye mixture of SYTO 9 (green) and hexidium iodide (red). Based on the growth condition of the co-culture model used, the images of the monoculture biofilms correspond to 42-h *Staphylococcus aureus* biofilm and 18-h *Pseudomonas aeruginosa* biofilm. Representative images were captured at a scale bar of 50 μm.

Thus, in the co-culture model, *S. aureus* biofilms were grown for 24 h with a regular 1 × 10^6^ CFU/ml starting inoculum density (Skogman et al., 2012) prior to the introduction of *P. aeruginosa* at around 1 × 10^3^ CFU/ml. These co-culture conditions enabled the high recovery of both bacterial species (Figure 2). Despite the presence of *P. aeruginosa*, *S. aureus* cell density remained constant for the vast majority of the incubation period. Only after the 18-h co-culture period did *S. aureus* experience a c.a. 1-log reduction of viability in co-culture (Figure 2A) when compared to the biofilm formed in monoculture, which reached an average density of 1.5 × 10^8^ CFU/ml. In turn, *P. aeruginosa* biofilms (slightly below 1 × 10^9^ CFU/ml) were not affected by the presence of *S. aureus* in co-culture. Furthermore, the architecture of the mono- and co-culture biofilms was visualized by fluorescence microscopy using a dye mixture composed of HI and SYTO 9 (Figure 3). The images of biofilms formed on the bottom of 24-well plates showed the co-existence of both bacterial species and suggested that high bacterial biomass adhered to the surface.

### Surfaceome and Exoproteome Dynamics: Protein Identification and Quantification

The surfaceome and exoproteome samples of co-cultured and monocultured *P. aeruginosa* and *S. aureus* biofilms, consisting of three biological replicates of each condition, were submitted to LC–MS/MS analysis. More than 2,200 proteins were identified by LC–MS/MS in both exoproteome and surfaceome samples after excluding potential contaminants (Supplementary Data Sheet 1). To avoid misinterpretation arising from potential cross-contamination in the LC–MS/MS analysis, proteins identified across both species in any sample (226) were excluded from further analysis in all conditions. Such cross-identifications were mostly low-intensity one-peptide identifications.

**FIGURE 4:**
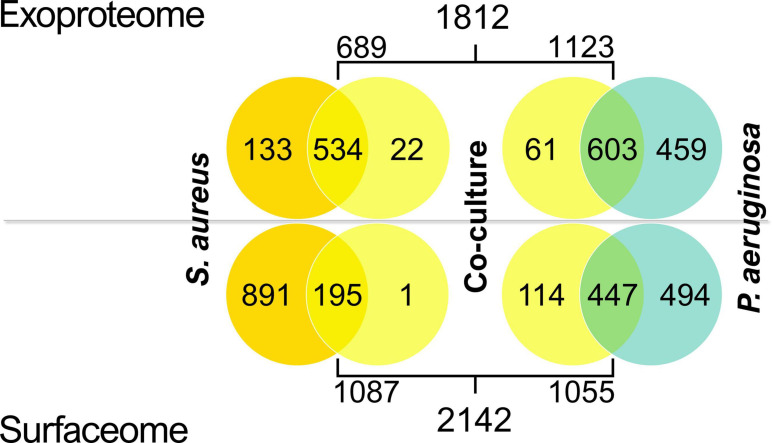
Venn diagrams displaying the number of valid identifications (present in at least two out of three replicate samples) in the exoproteomes and surfaceomes in *Staphylococcus aureus* and *Pseudomonas aeruginosa* co-culture samples.

Most exoproteome identifications were made in both the mono- and co-culture samples in both species, whereas the majority of surfaceome proteins had valid identifications only in the monocultures (Figure 4). This was especially observed in *S. aureus* surfaceomes, where over 80% of all identifications were made solely in monocultures. In *P. aeruginosa* surfaceomes, almost equal numbers of proteins were identified either in monocultures or in mono- and co-cultures.

In the exoproteome and surfaceome datasets, 28 and 43% of *P. aeruginosa* and *S. aureus* theoretical proteomes, respectively, were covered. The predicted pI and Mw values of the identifications between the datasets were illustrated in an *in silico* two-dimensional gel electrophoresis figure (Supplementary Figure 3). In both species and conditions, the analysis has favored the lower end of the pI distribution (the acidic mode). The higher frequency of identification of proteins with alkaline pI, especially in the surface-exclusive identifications of *S. aureus*, might indicate that trypsin-mediated surface shaving performed better in *S. aureus* than in *P. aeruginosa*. The distribution of the GRAVY indices of the identifications acted correspondingly (Supplementary Figure 4). As expected, few proteins with high hydrophobicity (more positive GRAVY) were recovered in the analysis.

To further test whether protein localization was reflected onto their identification, cross-tabulation analyses were carried out (Supplementary Table 1). In the exoproteomes, an association between secretion mode and the presence of a protein in the identifications was observed in both species (Fisher’s exact test, *p* < 0.001). The Sec and non-classically secreted proteins were slightly overrepresented in the exoproteome in *P. aeruginosa*, whereas proteins that lack the predicted means of exportation were considerably overrepresented in *S. aureus* exoproteome where only Sec proteins were present in higher-than-expected numbers. Proteins liberated by cell lysis can also be considered a part of the exoproteome (Armengaud et al., 2012), and their typically rapid degradation suggests that the observed differential patterns are governed by other phenomena than accumulation, as only the most stable proteins would remain in abundance in the supernatant. In the surfaceome samples, no connection between secretion mode and identification was seen in *P. aeruginosa* (Fisher’s exact test, *p* = 0.294). Conversely, a significant association was observed in *S. aureus* surfaceomes (*p* < 0.001), which contained significantly more proteins predicted to be intracellular. The presence of proteins without any predicted means of exportation, in addition to the high presence of the bifunctional autolysin Atl, could hint toward both extensive autolysis and the controlled release of cytoplasmic proteins into the environment (Pasztor et al., 2010; Ebner et al., 2015). Remarkably, it has been reported how, in some staphylococcal strains, the presence of Atl is linked to higher proportions of extracellular cytoplasmic proteins, but not to increased cell lysis, suggesting that the cytoplasmic proteins identified in the surfaceome had reached the cell surface during culturing (Dreisbach et al., 2020). Additionally, it has been observed how some cytoplasmic proteins reversibly associates with the cell surface in a pH-dependent manner, being recycled by *S. aureus* and moonlighting as component of the extracellular matrix (Foulston et al., 2014). Thereby, while cell lysis may largely contribute to the release of cytoplasmic proteins predicted to be intracellular, the moonlighting of cytoplasmic proteins *via* an unknown exportation pathway may account for the presence of some of these intracellular proteins on cell surfaces and extracellular milieu (Bendtsen et al., 2005). Moreover, given the stable cell density of *S. aureus* throughout biofilm development as well as the low percentage of lysed cells at the end of the incubation period (Figures 2A–D), it might be considered that the high percentage of such proteins in *S. aureus* surfaceomes and exoproteomes is not only due to cell lysis.

Based on non-normalized total peptide intensities, the protein with the highest intensity in LC–MS/MS analysis in *P. aeruginosa* exoproteomes was elastase LasB (Supplementary Table 2). This metalloprotease has a wide range of virulence-related functions, including biofilm formation, host cell invasion, and immune evasion (Cathcart et al., 2011). Redox-related protein azurin and probable peroxidase PA3529 were additionally included in the highest-intensity proteins in both mono- and co-culture exoproteomes of *P. aeruginosa*. Staphylolytic protease LasA, flagellar components, and probable phage proteins were specifically included in the top 10 co-culture exoproteins. The majority of the exoproteome identifications with highest intensities were predicted to be secreted *via* either the Sec-dependent or the non-classical pathways. In *S. aureus*, the top 10 most intense proteins were very similar between the two conditions (mono- and co-culture) (Supplementary Table 3). Interestingly, despite the lack of predicted secretion for most of the proteins, many of them have been identified as adhesive moonlighting proteins (Jeffery, 2018). These included, *e*.*g*., ArcB (ornithine carbamoyltransferase), GapA (glyceraldehyde-3-phosphate dehydrogenase), Atl (bifunctional autolysin), PdhB (pyruvate dehydrogenase E1 component subunit beta), Fba (fructose-bisphosphate aldolase), and DnaK chaperone.

The corresponding surfaceome catalogs showed a higher variability between the culture conditions when compared to the exoproteomes (Supplementary Tables 4, 5). In *P. aeruginosa* monoculture surfaceome, the highest-intensity proteins are involved in nutrient transport, fatty acid and carbohydrate metabolism, oxidation–reduction, and pigment biosynthesis (Supplementary Table 4). The most abundant *P. aeruginosa* proteins within the co-culture surfaceome included the membrane-associated ATP-synthase subunit beta, carbohydrate-metabolic protein SucB, redox proteins thiol:disulfide interchange proteins DsbA and Dps, as well as regulator proteins AlgP and DksA. Of these, AlgP and 50S ribosomal protein L2 (RplB) are predicted to have moonlighting functions and have been previously discovered on bacterial cell surfaces (Vecchietti et al., 2012; Wang and Jeffery, 2016).

As with the exoproteomes, the majority of the proteins identified as most intense in the *S. aureus* surfaceomes were cytoplasmic enzymes with reported moonlighting activity on the cell surface (Supplementary Table 5). In *S. aureus* monoculture, these included the arginine deiminase ArcA, GapA, GlpD (aerobic glycerol-3-phosphate dehydrogenase), AtpD (ATP synthase), PdhB (pyruvate dehydrogenase E1), TdcB (serine/threonine dehydratase), and DnaK, which have been previously detected *in vivo* in the biofilm matrix of *S. aureus* in a study performed in a rat model of bone implant infection (Lei et al., 2017). Correspondingly, the most intense proteins in the co-culture surfaceome also included several which were identified in the implant-associated *S. aureus* biofilm matrix (Lei et al., 2017), namely, ArcA (arginine deiminase), Mqo (malate:quinone oxidoreductase), RpsA and RpsB (30S ribosomal proteins), and GapA.

**FIGURE 5:**
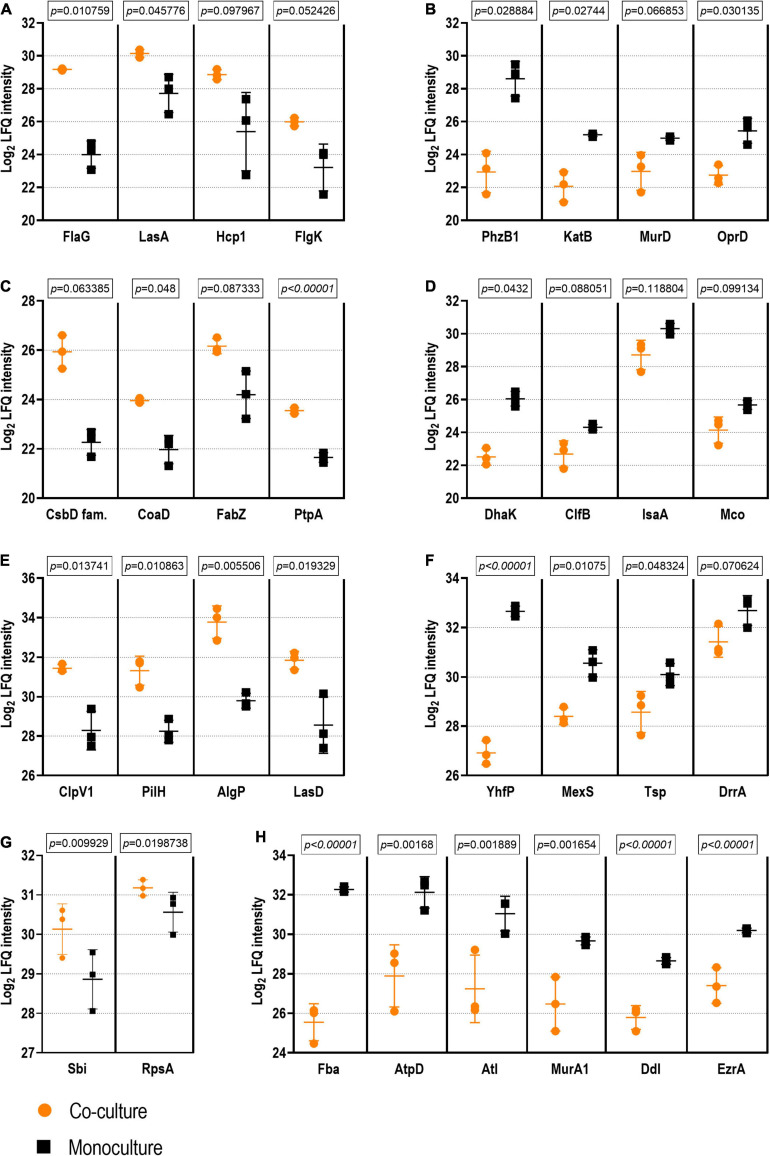
Examples of proteins with statistical differences of abundance in co-culture *vs*. monoculture. The exoproteome is represented by examples of *Pseudomonas aeruginosa* proteins with higher **(A)** and lower abundance **(B)** as well as by examples of *Staphylococcus aureus* proteins with higher **(C)** and lower abundance **(D)** in co-culture *vs*. monoculture. The surfaceome is represented by examples of *P. aeruginosa* proteins with higher **(E)** and lower abundance **(F)** as well as by examples of *S. aureus* proteins with higher **(G)** and lower abundance **(H)** in co-culture *vs*. in monoculture. Student’s *t*-test *q*-Values (permutation-based false discovery rate) are presented for each comparison (see Supplementary Tables 6–9 for the complete dataset).

Regarding protein quantification, a total of 762 and 498 proteins were quantified in the *P. aeruginosa* and *S. aureus* exoproteomes, respectively. In the surfaceomes, 495 proteins were quantified in *P. aeruginosa* and 348 in *S. aureus* (with the threshold LFQ applied, as described in Section “Proteomic Data Processing and Bioinformatic Analysis”). The complete dataset of differentially expressed proteins is available as the Supplementary Tables 6-9. These data were used for a pathway enrichment analysis to identify putative biological changes arising from the co-culture condition (presented in the following sections “Effect of Co-culture Condition on Exoproteomes” and “Follow-up Studies” for exoproteomes and surfaceomes, respectively). The representative examples of differential expression are shown in Figure 5.

### Effect of Co-culture Condition on Exoproteomes

#### Co-culture-Induced Changes in *P. aeruginosa* Exoproteomes

The KEGG pathway enrichment in the culture conditions of *P. aeruginosa* (mono- or co-culture) was assessed using the STRING database (Szklarczyk et al., 2019) using the catalogs of differentially expressed proteins (FDR, 0.15) that were more abundant in the given condition. In *P. aeruginosa* exoproteome, 35 pathways were found to be enriched in monoculture and three in co-culture conditions (Supplementary Figure 5). Multiple pathways related to amino acid metabolism were specifically enriched in the monocultured *P. aeruginosa*. Other examples of monoculture-specific enrichments included the biosynthesis of amino acids, glycolysis/gluconeogenesis, microbial metabolism in diverse environments, and pyruvate metabolism. Although all these pathways concern intracellular activities, it is not uncommon to find such proteins in the exoproteome. Proteins involved in translation, carbon metabolism, and amino acid metabolism have been previously identified in *P. aeruginosa* exoproteomes (Choi et al., 2011; Couto et al., 2015). The proposed explanations for the phenomenon include the deliberate non-classical secretion of these proteins as extracellular moonlighters and their increased non-specific liberation *via* the basal levels of cell lysis (Couto et al., 2015; Turnbull et al., 2016). The latter interpretation could indicate the monocultured *P. aeruginosa* as having an overall increased metabolism.

**FIGURE 6:**
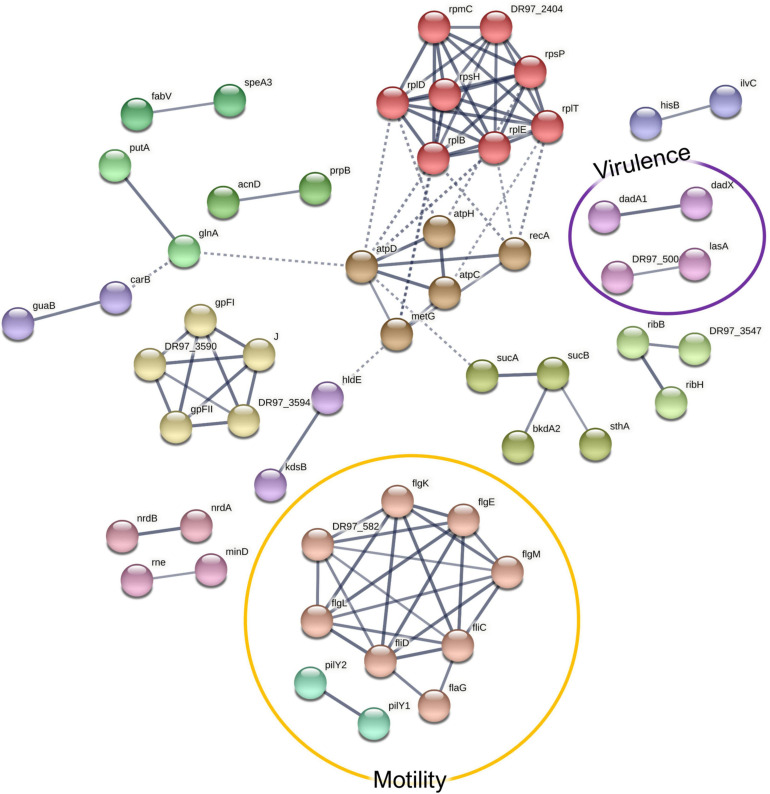
A STRING database (Szklarczyk et al., 2019) network of interacting proteins with a significantly higher abundance in *Pseudomonas aeruginosa* co-culture *versus* monoculture exoproteome. Due to the large number of interactors, only high-confidence interactions are displayed.

In the co-culture conditions, the specifically enriched cellular pathways included flagellar assembly- and ribosome-mediated activities (Figure 6 and Supplementary Figure 5). In this setting, 80 proteins were significantly more highly abundant (FDR, 0.15; Supplementary Table 6), of which the consistent overabundance of flagellar proteins implies that *P. aeruginosa* becomes more motile in the presence of *S. aureus*. Detached flagella are well known contaminants in cell-free supernatants, but the more they are expressed, the more these proteins can be expected to shed into the supernatant (Gerstel et al., 2009). On the other hand, the concurrent increase of flagellar hook proteins (FlgE, FlgL, and FliD) in the supernatant could point toward the intentional ejection of intact flagella, which represents a recently described mechanism of γ-proteobacteria to conserve energy upon starvation (Ferreira et al., 2019). To shed some light on the effects of the co-culture conditions on the motility of *P. aeruginosa*, a motility assay was carried out, which is further described in Section “Co-culturing With *S. aureus* Increases *P. aeruginosa* Motility.”

Anti-staphylococcal proteins, such as LasA (the staphylolytic toxin), and another associated aminopeptidase (Ravichandran et al., 2015) were more abundant in the co-culture setting. This is an expected result, given the exposure of *P. aeruginosa* to *S. aureus* peptidoglycan (Korgaonkar et al., 2013). This might have important pathogenic implications as the peptidase may act upon human proteins, such as elastin (Spencer et al., 2010), which has been shown to promote epithelial cell invasion (Cowell et al., 2003). Another co-culture-enriched *P. aeruginosa* protein was Hcp1, a component of the type VI secretion system (T6SS), which has been shown to be induced in response to a mixed species biofilm environment, where it enables the outcompeting of other species (Cheng et al., 2019). Together with the anti-staphylococcal proteins, it has been related with a higher virulence toward the host in the pathogenesis of chronic pulmonary infection in CF (Mougous et al., 2006). Correspondingly, the T6SS secretion machinery proteins ClpV1, ClpB, and TssC were more abundant in the co-cultured *P. aeruginosa* surfaceomes. Other pathogenicity-associated proteins more abundant in the co-culture conditions included a putative ferric enterobactin esterase, two hemagglutinins, an immune evasive protease—MucD (Mochizuki et al., 2014), and two members encoded by the *dad* operon, which contribute to biofilm formation and rhamnolipid production (Oliver and Silo-Suh, 2013).

**FIGURE 7:**
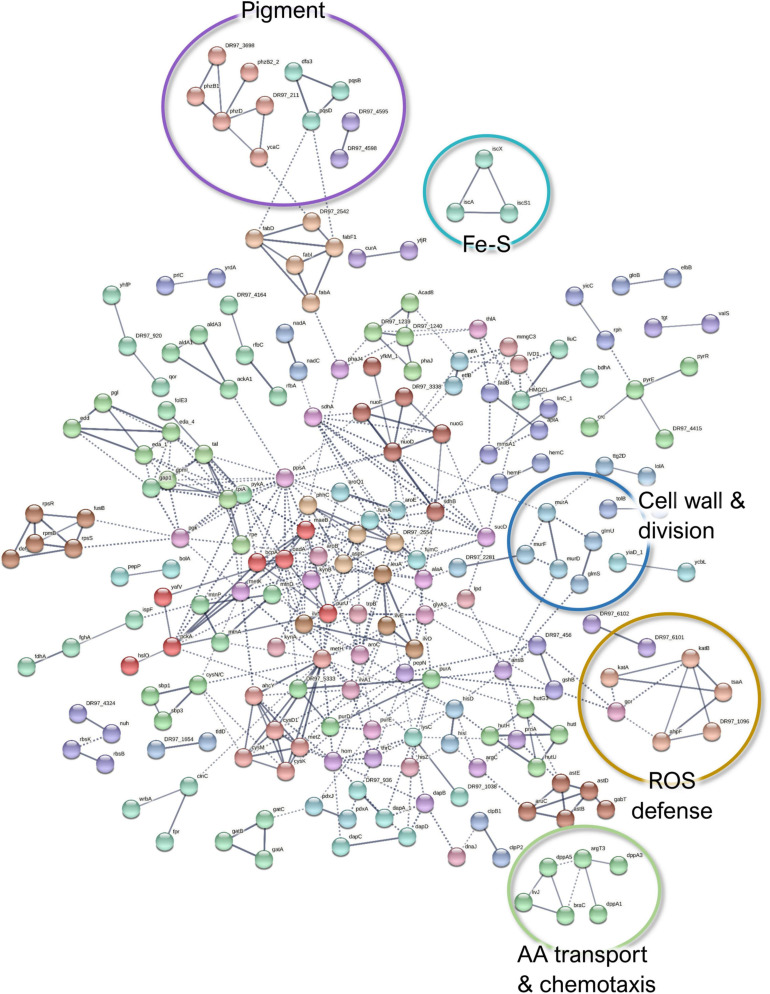
A STRING database (Szklarczyk et al., 2019) network of interacting proteins with a significantly lower abundance in *Pseudomonas aeruginosa* co-culture *versus* monoculture exoproteome. Due to the large number of interactors, only high-confidence interactions are displayed.

When comparing the co-culture- and the monoculture-associated exoproteomes, 300 *P. aeruginosa* proteins were identified as less abundant in co-culture conditions (Figure 7). These proteins are predicted to be involved in oxidative stress response, cell wall biosynthesis and membrane translocation, iron–sulfur (Fe–S) cluster assembly, TCA cycle/carbohydrate metabolism, and amino acid transport and metabolism (Supplementary Figure 5). Additionally, proteins related to phenazine pigment biosynthesis (PhzB1, PhzD, PhzB2_2, PqsD, PqsB, and Dfa3) were significantly enriched in the monoculture (Supplementary Table 6). Given the evident change in phenotype, this was further explored by the follow-up study described in Section “*P. aeruginosa* Produces More Pigments in Monoculture Than in Co-culture.”

Among the ones involved in oxidative stress response, the catalases KatA and KatB, three alkylhydroperoxide reductase components (AhpD, AhpF, and AhpC/TsaA), the chaperonin HslO, and three quinone oxidoreductases were significantly less abundant in *P. aeruginosa* exoproteomes under co-culture conditions compared to the monoculture conditions. Although these proteins are known for their intracellular activities, they may be distributed to different subcellular locations, including the extracellular fractions (Ochsner et al., 2000). For example, KatA can be secreted and is predicted to remain for prolonged periods in the extracellular milieu, conferring protective effects against reactive oxygen species (ROS) (Hassett et al., 2000). The decreased abundance of ROS defense proteins in *P. aeruginosa* could indicate that cells are less exposed to oxidative stress in the presence of *S. aureus*. This could be attributed either to the different physicochemical characteristics of the co-culture environment or the use of the redox stress proteins present in the *S. aureus* surfaceomes or exoproteomes as “public goods”. The downregulation of most of the Fe–S cluster biosynthetic proteins could also be associated with less oxidative stress (Romsang et al., 2014). However, this could also imply that there is more iron available as the increase of Fe–S cluster proteins has been associated with a response to iron starvation (Nelson et al., 2019), and it has been previously reported that the iron acquisition-related genes in *P. aeruginosa* are downregulated when this species is co-cultured with *S. aureus* (Mashburn et al., 2005). Whether this translates to protein changes outside of the cells remains to be shown. This also applies to the TCA cycle; carbohydrate fatty acids and amino acids are not expected in the exoproteome, but virulence-related moonlighting functions have been suggested for them (Henderson and Martin, 2013; Henderson, 2014).

Finally, the decreased abundance of several cell wall biosynthesis proteins (*e.g*., UDP-N-acetylmuramoylalanine-D-glutamate ligase MurD, MurF, MurA, and Tol-Pal system protein TolB) could point toward more persistent *P. aeruginosa* in co-culture. These findings, in addition to the lower abundance of MexS (anoxidoreductase) and OprD (porin D) with known contribution to antibiotic efflux (Sobel et al., 2005), could indicate that *P. aeruginosa* has a higher tolerance to antibiotics when co-cultured with *S. aureus.* Examples of the differentially expressed proteins of *P. aeruginosa* exoproteome are presented in Figures 5A,B.

#### Co-culture-Induced Changes in *S. aureus* Exoproteomes

In the *S. aureus* exoproteome, 498 proteins were quantified, of which 39 were significantly more abundant (FDR, 0.15) in co-culture than in monoculture conditions (Supplementary Table 7). The proteins with a slightly higher abundance in the co-culture exoproteome included a group of ribosomal proteins (Supplementary Figure 6). Ribosomal proteins have been proposed to stabilize/strengthen the biofilm matrix (Graf et al., 2019). A total of 90 proteins were more abundant in the *S. aureus* monoculture *versus* in the co-culture conditions (Supplementary Table 7). The monoculture-enriched pathways included aminoacyl-tRNA biosynthesis, glycolysis/gluconeogenesis, and microbial metabolism in diverse environments (Supplementary Figure 7).

**FIGURE 8:**
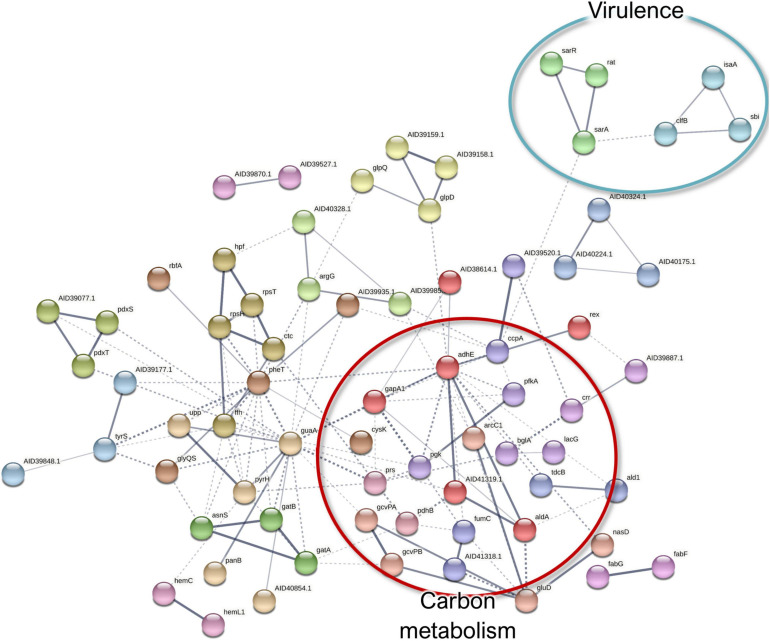
A STRING database (Szklarczyk et al., 2019) network of interacting proteins with a significantly lower abundance in *Staphylococcus aureus* co-culture *versus* monoculture exoproteome.

Although some proteins involved in carbohydrate metabolism and translation were more highly abundant in the co-culture exoproteomes, a more significant abundance of such proteins was observed in monoculture conditions (Figure 8). The presence of this proteins on the exoproteome is probably given by non-specific liberation due to cell lysis, and they suggest a lower metabolic activity and, thus, a higher degree of a persistence-like-state *S. aureus* when cultured in the dual-species biofilm (Orazi and O’toole, 2017). On the other hand, cytoplasmic proteins are known to be excreted by *S. aureus* to enhance invasiveness and tackle host immunity (Ebner et al., 2016), which, in addition to the observed higher abundance of pathogenicity proteins (IsaA, ClfB, and Sbi), could provide hints of higher virulence in monocultured *S. aureus*. Examples of the differentially expressed proteins of *S. aureus* exoproteome are presented in Figures 5C,D.

#### Co-culture-Induced Changes in *P. aeruginosa* Surfaceomes

In *P. aeruginosa* surfaceomes, pathways of amino acid metabolism, glycolysis/gluconeogenesis, and microbial metabolism in diverse environments were enriched in the co-cultured biofilms (Supplementary Figure 8 and Supplementary Table 8). Indeed the outcomes in the surfaceome profile indicated a generally higher metabolism in co-cultures as opposed to the corresponding exoproteome samples. Based on the general nature of these metabolic pathways, the implication of this disparity may be coincidental, as different proteins contribute to enrichments in the different datasets.

A total of 204 proteins were altogether significantly more abundant (FDR, 0.15) in the co-culture samples compared to the monocultures (Supplementary Figure 9 and Supplementary Table 8). Among them, the peptidoglycan binding protein FimV and the motility proteins PilH and PilJ are involved in twitching motility (Delange et al., 2007; Wehbi et al., 2011), which agrees with the detected increase in motility-related protein abundances in the exoproteome. Recent studies demonstrated that the type 4 pili are upregulated in mixed-species conditions, which helps *P. aeruginosa* to compete with other bacterial species (Cheng et al., 2019), including *S. aureus* species, thereby leading to a more aggressive mixed-species infection (Limoli et al., 2019).

Two alginate regulatory proteins, AlgP and AlgU, were strongly enriched proteins in the *P. aeruginosa* surfaceome with 16- and threefold upregulation, respectively. Besides its function in the intracellular space, studies have identified AlgP at the cell surface and in outer membrane vesicles produced by *P. aeruginosa* (Toyofuku et al., 2012; Vecchietti et al., 2012). It has been suggested that such vesicles facilitate intraspecies communication (Jan, 2017), so it is possible that the export of these regulators has a role in shaping the mucoid phenotype in the biofilm community.

DsbA and DsbC, thiol:disulfide interchange proteins, were also more abundant in co-culture surfaceomes. These proteins assist in the proper folding of secreted enzymes harboring disulfide bridges, many of which are recognized as factors contributing to virulence and immune evasion (Ha et al., 2003; Heras et al., 2009). The components of the Dsb machinery have therefore been identified as potential antibacterial drug targets (Heras et al., 2015). In this study, the increased abundance of these proteins in the *P. aeruginosa* surfaceome, in the presence of *S. aureus*, could be linked with the increased secretion of lytic toxins as means for extracting iron and acquiring a competitive advantage (Ravichandran et al., 2015). Furthermore, the Dsb system is essential for *P. aeruginosa* motility (Urban et al., 2001) and pilus-driven twitching motility by facilitating proper protein folding (Ha et al., 2003).

**FIGURE 9:**
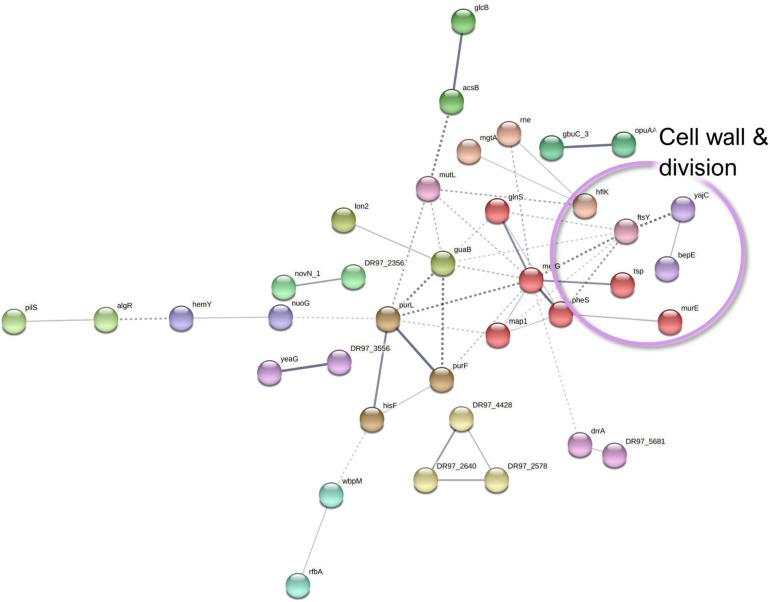
A STRING database (Szklarczyk et al., 2019) network of interacting proteins with a significantly lower abundance in *Pseudomonas aeruginosa* co-culture *versus* monoculture surfaceome.

In the *P. aeruginosa* surfaceome, 61 proteins that belong to diverse functional categories were less abundant in co-culture conditions compared to the monoculture. From these, only the aminoacyl-tRNA synthetase-mediated cellular pathways were dominating (Supplementary Figure 8 and Supplementary Table 8), implying a lower translational activity and an increased persistence in co-culture conditions. Several aminoacyl-tRNA ligases have been detected on previous surfaceome explorations and have been linked with their membrane association at membrane protein translation or with so-far-unknown moonlighting functions (Wang and Jeffery, 2016). In addition, the interactors include other proteins of interest with putatively important implications in terms of virulence and tolerance (Figure 9 and Supplementary Table 8).

The ABC transporter component (drrA) and the co-expressed oxidoreductase MexS (DR97_5681) were markedly with lower abundance in the dual-species biofilm surfaceome (Supplementary Figure 8). Of note is that MexS was also downregulated in the dual-species biofilm exoproteomes. Since MexS mediates the repression of T3SS (Jin et al., 2011), its lower expression here is in harmony with the hints of increased T3SS expression. As mentioned in Section “Co-culture-Induced Changes in *P. aeruginosa* Exoproteomes,” this downregulation may imply phenotypic antimicrobial tolerance in co-culture.

Furthermore, several *P. aeruginosa* proteins involved in cell wall and outer membrane biogenesis, as well as protein translocation into the cell membrane, demonstrated a lower abundance in co-culture conditions. MurE (UDP-N-acetylmuramoyl-L-alanyl-D-glutamate-2,6-diaminopimelate ligase) and the periplasmic tail-specific protease Prc (tsp) are involved in cell wall peptidoglycan biosynthesis and cell division, whereas a modulator protein, HflK, signal recognition particle receptor FtsY, and preprotein translocase subunit YajC have a role in membrane protein trafficking. This could altogether imply decreased cell division as discussed with the exoproteome findings.

Among the low-abundance proteins, nucleotide sugar epimerase/dehydratase WbpM and glucose-1-phosphate thymidylyltransferase (RmlA) have been proposed to participate in the biosynthesis of O-antigen for the outer membrane lipopolysaccharide (Rocchetta et al., 1999). Although the lower abundance of these proteins correlates to the decreased outer membrane biosynthesis and to halted division (Schäkermann et al., 2013), the differential O-antigen synthesis may also affect biofilm architecture and persistence in *P. aeruginosa* infections (Huszczynski et al., 2019). In addition, decreased O-antigen is linked with immune evasiveness and biofilm adaptation (Maldonado et al., 2016). The reduced abundances of WbpM and RmlA in co-culture conditions could be linked with increased T3SS-mediated secretion, virulence, and persistence in infections (Augustin et al., 2007).

As detected with the *P. aeruginosa* exoproteome, two proteins involved in iron acquisition (Fpr, ferredoxin-NADP reductase and a FecR family protein) were less abundant in the co-cultured *P. aeruginosa* biofilm surfaceome. Examples of the differentially expressed proteins of *P. aeruginosa* surfaceome are presented in Figures 5E,F.

#### Co-culture-Induced Changes in *S. aureus* Surfaceomes

In *S. aureus*, five and 327 proteins demonstrated significant (FDR, 0.15) up- and downregulation, respectively. Only the immunoglobulin-binding protein Sbi, an immune evasion factor (Smith et al., 2012), was increased more than twofold in the co-culture, whereas most of the downregulated proteins had a more substantial decrease (Supplementary Figure 10 and Supplementary Table 9). Of note is that Sbi demonstrated a contrasting behavior in the *S. aureus* exoproteomes, which might be explained by its increased abundance and/or translocation as an early response to the co-culture condition. On the other hand, other virulence factors were detected as remarkably less abundant, which included the MSCRAMM family adhesin SdrC and the adhesive moonlighting chaperone DnaK.

**FIGURE 10:**
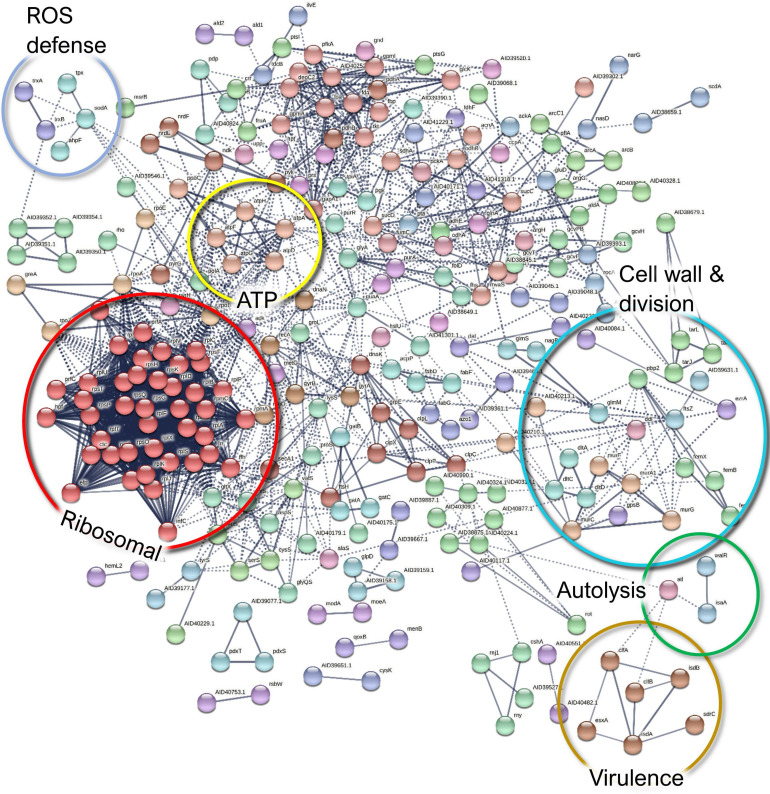
A STRING database (Szklarczyk et al., 2019) network of interacting proteins with a significantly lower abundance in *Staphylococcus aureus* co-culture *versus* monoculture surfaceome.

Among the low-abundance proteins, septation ring formation regulator EzrA and the ATP-dependent metallopeptidase FtsH/Yme1/Tma family protein were identified together with the cell division protein FtsZ (Figure 10). These three proteins are involved in cell division, and this finding may be related to the known ability of *P. aeruginosa* to halt *S. aureus* growth (Mitchell et al., 2010), thus indicating increased *S. aureus* persistence in the co-culture. Moreover, the downregulation of proteins involved in TCA cycle, carbon metabolism, ATP synthesis, and translation (Supplementary Figure 10 and Supplementary Table 9) also provides indirect evidence for the decreased *S. aureus* metabolism in the presence of *P. aeruginosa*, although the putative moonlighting functions of these proteins may be unrelated. Furthermore, ribosomal proteins were markedly more abundant among the monoculture-enriched proteins of *S. aureus*. These proteins contribute to the biofilm biomatrix stability (Graf et al., 2019) but might also indirectly indicate lower levels of translational activity in the co-cultured *S. aureus*.

Finally, ribitol phosphate teichoic acid biosynthesis proteins (TarI, TarL, and TarJ), which contribute to cell wall biogenesis, as well as several proteins involved in autolysis were less abundant in co-culture. These include the bifunctional autolysin Atl, the transglycosylase IsaA, and the transcriptional regulator proteins WalR. Examples of the differentially expressed proteins of *S. aureus* surfaceome are presented in Figures 5G,H.

### Follow-Up Studies

#### *Pseudomonas aeruginosa* Produces More Pigments in Monoculture Than in Co-culture

**FIGURE 11:**
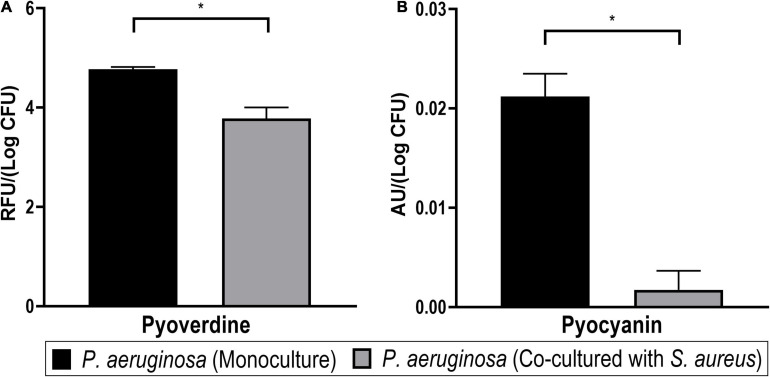
Pigment production of *Pseudomonas aeruginosa* in mono- and dual-species biofilms. Significant differences of both pyoverdine **(A)** and pyocyanin **(B)** pigments were detected between both cultures (Student’s *t*-test, *p < 0.05). Bars represent the average of two biological repetitions with their SD.

Many pathogenic strains of *P. aeruginosa* produce pigment molecules, such as pyocyanin and pyoverdine, which are connected to iron acquisition and interspecies interactions. In our dual-species biofilm model, *P. aeruginosa* cultures, in the absence of *S. aureus*, were visually pigmented. Furthermore, the proteomics analyses pointed toward the higher abundance of pigment biosynthesis proteins (such as those from the *phz* and *pqs* operons) and iron acquisition-related proteins (such as Fe–S cluster assembly, Fpr, ferredoxin-NADP reductase, and a FecR family protein) when this bacterium is monocultured. To confirm these findings, the pigmentation phenotype of *P. aeruginosa* was quantified in mono- and co-culture biofilms (Figures 11A,B).

The siderophore pyoverdine, a fluorescent yellow-green pigment, is a high-affinity iron chelator that has an important role in providing sufficient iron for biofilm formation in *P. aeruginosa* (Banin et al., 2005). The biosynthesis of siderophores is also connected to quorum sensing regulation (Stintzi et al., 1998; Diggle et al., 2007). In this study, the relative quantification of pyoverdine revealed significantly lower levels of the pigment in the co-cultured biofilm supernatants (Figure 11A). Since a lower expression of iron-acquisition proteins was observed in the exoproteome and the surfaceome of *P. aeruginosa* in co-culture, it possibly indicates a reduced need for these proteins in this condition. Thus, the reduced production of pyoverdine could possibly also reflect that iron is more readily available for *P. aeruginosa* in the presence of *S. aureus*, which would warrant the downregulation of pyoverdine production in co-culture. This result seems to be in agreement with that of Mashburn et al. (2005). However, it is not ruled out that the decrease in pyoverdine in co-cultured *P. aeruginosa* might result from a change in quorum sensing signaling or other pathways.

In addition, pyocyanin pigment was quantified by acidifying aliquots of the supernatant to pH < 2, which is expected to bring the molecule to a charged state with a characteristic red color, which was measured *via* changes in *A*_520__*nm*_ (Essar et al., 1990; Debritto et al., 2020). It was confirmed that pyocyanin was significantly less produced by the co-cultured *P. aeruginosa* biofilm supernatants (Figure 11B). This outcome is in agreement with a previous report of pyocyanin expression in *P. aeruginosa* being decreased in co-culture with *S. aureus* (Tognon et al., 2019). Like pyoverdine, pyocyanin is also involved in the iron acquisition of *P. aeruginosa* biofilms (Wang et al., 2011). On the other hand, it has also been demonstrated that this pigment is toxic against staphylococci (*in vitro*) (Biswas et al., 2009) and mammalian cells (*in vivo*) (Lau et al., 2004) and may be induced as a response to *S. aureus* peptidoglycan (Korgaonkar and Whiteley, 2011; Yang et al., 2020). This makes the lower pyocyanin production an exception for the generally higher virulence (as derived from the proteomics profiles) in co-cultured *P. aeruginosa*.

#### Co-culturing With *S. aureus* Increases *P. aeruginosa* Motility

According to our findings, both the exoproteome and the surfaceome analyses support that *P. aeruginosa* demonstrates higher-motile phenotype when co-cultured with *S. aureus*. These findings are in alignment with recent reports (Limoli et al., 2019). However, the increased presence of flagellar components in the co-culture supernatant, combined with the higher observed abundance of flagellar hook proteins, could also indicate the intentional ejection of intact flagella. This could support the opposite interpretation that *P. aeruginosa* actively reverts the motile phenotype when co-cultured with *S. aureus* (Ferreira et al., 2019). A motility assay was carried out to study the phenotypic presentation of the observed phenomena.

**FIGURE 12:**
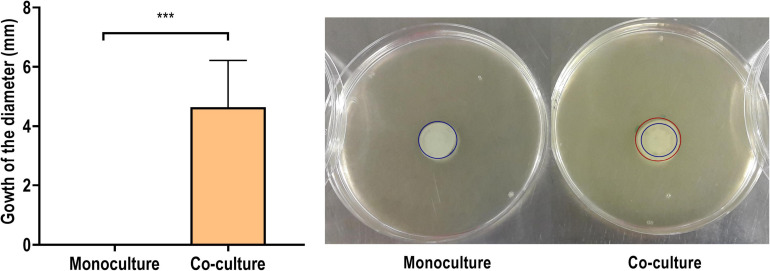
Increase on the diameter (in mm) of a 50-μl inoculum obtained from the combination of the biofilm and the supernatant of a *Pseudomonas aeruginosa* PAO1 biofilm formed in monoculture or in co-culture with *Staphylococcus aureus* ATCC 25923 after 8 h of incubation in soft agar. In the representative images on the right, the blue circle represents the initial diameter and the red circle the grown diameter. The results are expressed as the mean of five biological repetitions with their SD (Student’s *t*-test, ***p < 0.001).

Figure 12 shows the development of a colony diameter at 8 h of incubation, as compared to the initial inoculum diameter, of *P. aeruginosa* mono- and co-cultured in the conditions of the proteomics analysis. The colonies derived from a *P. aeruginosa* biofilm co-cultured with *S. aureus* grew up to 3.9 ± 0.9 mm, while no growth was observed in the corresponding monoculture samples (*p* < 0.001). It was additionally confirmed that this increase of the bacterial population’s diameter was only due to *P. aeruginosa*, as the presence of *S. aureus* was not detected in the distal colony parts.

These results offer confirmation for the proteomics-derived notion of the higher motility of *P. aeruginosa* in co-culture with *S. aureus*. However, in this experimental setting, it cannot be determined whether the changes in *P. aeruginosa* motility are only phenotypical or have arisen from accumulative mutations promoted by the presence of *S. aureus* (Tognon et al., 2017*)*. Additionally, while this assay provides evidence of the increased motility, further analysis would be needed to distinguish the specific type of motility increased. The abundance of flagellar proteins in the supernatant, reflected in the exoproteome, could mean an increase of the swimming and swarming types of motility, while the increased expression of peptidoglycan binding protein FimV and the proteins PilH and PilJ reflects an increase in twitching motility (Schniederberend et al., 2019). In any case, differences in motility are associated with changes in the virulence toward the host (Kazmierczak et al., 2015; Guoqi et al., 2018). Flagella and type 4 pili are important in the early stages of infection, as they allow *P. aeruginosa* to attach and colonize surfaces (Kazmierczak et al., 2015) while facilitating the subsequent biofilm formation (Liaqat et al., 2019). The proteins also help the bacteria escape from surfaces when desirable (Burrows, 2012) and disperse through host tissue, further disseminating the infection (Kazmierczak et al., 2015).

#### *Pseudomonas aeruginosa* Is Less Susceptible to Antibiotics in Co-culture Condition

Multi-species biofilms have been associated with a higher tolerance toward antibiotic therapy in different types of infections. Evidence of decreased cellular division and, indirectly, of general metabolism and protein synthesis in the co-cultured bacteria was acquired in this proteomic exploration as well. We therefore studied whether such increased tolerance was also observed in the co-culture developed in this assay.

**FIGURE 13:**
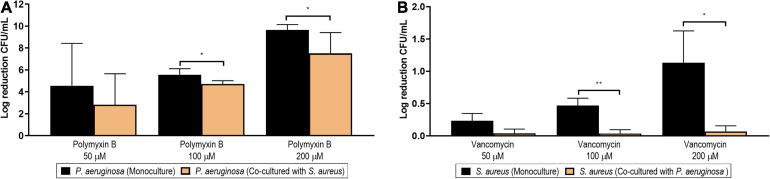
Log reduction of the viable counts (colony-forming unit/ml) of *Pseudomonas aeruginosa* PAO1 **(A)** and *Staphylococcus aureus* **(B)** biofilms after the treatment with different concentrations of polymyxin B (50, 100, and 200 μM) and vancomycin (50, 100, and 200 μM), respectively. The black columns represent the log reduction achieved in monoculture conditions, while the orange columns represent the log reduction in co-culture conditions. The results are expressed as the mean of three or more biological repetitions with their SD (Student’s *t*-test; *p < 0.05 and **p < 0.01).

Figure 13A shows the log reduction achieved by polymyxin B at different concentrations (200, 100, and 50 μM) in *P. aeruginosa* biofilms formed in monoculture (black columns) or in co-culture with *S. aureus* (orange columns). The effects of polymyxin B on the viable counts of *P. aeruginosa* are additionally presented in Supplementary Figure 11A as non-normalized log CFU/ml. At a concentration of 200 μM, polymyxin B managed a complete eradication of the monoculture biofilm. However, the same complete inhibitory effect was not observed against the dual-species biofilm, as a significant difference was observed between the log reduction in mono- and co-culture (*p* = 0.018, black bar *vs*. orange bar). At a concentration of 100 μM, a significant biofilm reduction of over four logs was achieved on the biofilm in co-culture, but this reduction was still lower than the one achieved against the biofilm developed in monoculture (*p* = 0.024, black bar *vs*. orange bar). No differences were observed when comparing the log reduction generated by the lowest concentration tested (50 μM).

The most accepted mechanism of action (MoA) for polymyxin B is the interaction with the lipopolysaccharides of Gram-negative bacteria, leading to permeability changes in the outer membrane (Zavascki et al., 2007). However, alternative mechanisms have been proposed. Among them, the hydroxyl radical death pathway (HRDP) theory postulates that the bactericidal effect is caused by the ROS generated by polymyxin B *via* the Fenton reaction (Imlay et al., 1988). Such an increase in ROS production after polymyxin B treatment has been reported in both planktonic and sessile *P. aeruginosa* (Lima et al., 2019). It has been speculated that mutants deficient in iron import and/or reduction might have a higher tolerance to antibiotics (Yeom et al., 2010). Although our exoproteome data showed downregulated proteins with roles in iron acquisition in dual-species biofilm, we cannot conclude that this is directly caused by a higher abundance of iron or a decreased level of Fe–S-damaging oxidative stress promoted by the higher abundance of redox stress proteins in the *S. aureus* surfaceome. The other suggested MoA is the interaction of polymyxin B with the cell division machinery (Trimble et al., 2016). Our proteome data showed that several proteins involved in cellular division and cell wall biosynthesis were downregulated in the mixed dual-species biofilm. Whichever the underlying mechanism is, our finding altogether suggests that the environmental changes induced by co-culturing with *S. aureus* promote a phenotypic tolerance to polymyxin B in *P. aeruginosa* biofilms.

Finally, the decreased expression of MexS and porin D, among the other proteomic changes pointing toward a less metabolically active phenotype of co-cultured *P. aeruginosa*, suggests that increased unspecific tolerance would likely be also seen against the various other antibiotics that require active cell division and metabolism to exert their effect. In addition, other antibiotic families, including fluoroquinolones, aminoglycosides, or β-lactams, have also been reported to act through the HRDP (Dwyer et al., 2009), so a higher tolerance to those could also be expected.

#### *Staphylococcus aureus* Is Less Susceptible to Antibiotics in Co-culture Conditions

Consistent with the results seen in *P. aeruginosa*, co-cultured *S. aureus* biofilms were also more resilient to antibiotic treatment in comparison to monocultures. As shown in Figure 13B, the log reduction achieved in monoculture with vancomycin at 200 and 100 μM differed significantly from the respective biofilms formed in co-culture (*p* = 0.021 and *p* = 0.004, when comparing the black and orange columns of 200 and 100 μM, respectively). The effects of vancomycin on the viable counts of *S. aureus* are additionally presented in Supplementary Figure 11B as non-normalized log CFU/ml.

Of note is that it is not surprising that the log reduction achieved in *S. aureus* monoculture is only of 1-log at such a high concentration (200 μM), as the older age and the associated lower metabolic activity of this biofilm are probably contributing to its higher tolerance (Stewart, 2015). The lower anti-biofilm activity of vancomycin against the co-cultured *S. aureus* may be associated with the downregulation of the cell division proteins identified in the surfaceome, as its antibacterial activity is based on the inhibition of cell wall synthesis. Alternatively, vancomycin is also known to inhibit transglycosylase, which was also among the lower-abundance proteins identified in the co-cultured *S. aureus* surfaceome. Interestingly, several proteins related with autolysis were also less abundant in the co-cultured *S. aureus* surfaceome, pointing toward a decreased autolysis, which has previously been associated with increased vancomycin tolerance in *S. aureus* (Koehl et al., 2004).

These findings align well with previous reports describing an increased tolerance to antibiotics associated with dual-species biofilms, involving both Gram-negative and Gram-positive bacteria (Orazi and O’toole, 2017; Cendra et al., 2019).

## Conclusion

In this study, the dual-species biofilm formation of two clinically relevant bacterial species (*P. aeruginosa* strain PAO1 and *S. aureus* strain ATCC 25923) was characterized by label-free proteomic analyses targeting the surfaceomes and exoproteomes. The proteome analyses between the mono- and co-cultured biofilms indicated the enrichment of virulence-related proteins in *P. aeruginosa* in the presence of *S. aureus*, whereas *P. aeruginosa* produced several stress response proteins at a lower level under the same condition. Thus, our findings indicate that *S. aureus* protects *P. aeruginosa* from oxidative stress, thereby contributing to its tolerance and virulence. Changes in *P. aeruginosa* proteomes suggest increased motility, decreased iron acquisition, and the downregulation of pigment biosynthesis proteins. Pathogenic moonlighting proteins were differentially abundant in mono- and co-cultured *S. aureus* biofilms, and proteomics-based evidence of lower reproductive activity was obtained. Phenotypic confirmations indicated a higher production of pyoverdine and pyocyanin pigments in monocultured *P. aeruginosa* biofilms, possibly due to a higher iron availability in the co-culture environment. A more motile phenotype in the presence of *S. aureu*s was in line with the increased abundance of motility-related proteins in co-cultured *P. aeruginosa* biofilm exoproteomes and surfaceomes. Finally, the co-cultured biofilms demonstrated a higher tolerance to antibiotics in both species, which highlights the importance of assessing the efficacy of antimicrobial compounds on settings that more faithfully resemble the ones where biofilms are found in the clinic. The present study shows the applicability of label-free proteomics for simultaneous surfaceomic and exoproteomic analyses of mixed-species biofilms, which is of crucial importance for developing more effective tools to eradicate biofilms in clinical settings.

## Data Availability Statement

The mass spectrometry proteomics data have been deposited to the ProteomeXchange Consortium via the PRIDE (Perez-Riverol et al., 2019) partner repository with the dataset identifier PXD023445.

## Author Contributions

SG-G, PS-M-G, IR, IM, and AF contributed to the conceptualization. KS, SG-G, IM, JC, PS-M-G, and IR contributed to the methodology. IM and JC took charge of the software. IM, SG-G, PS-M-G, IR, and AF contributed to the validation. PS-M-G, IR, and IM contributed to the formal analysis. SG-G, IM, PS-M-G, and IR contributed to the investigation. KS, VC, AF, and TN contributed to the resources. IM contributed to the data curation. IM, IR, and PS-M-G contributed to the original draft preparation. SG-G, IM, KS, TN, JC, VC, and AF contributed to the review and editing. SG-G, IM, PS-M-G, IR, and AF contributed to the visualization. IM, AF, and KS contributed to the supervision. AF carried out the project administration and funding acquisition. All the authors have read and agreed to the published version of the manuscript.

## Conflict of Interest

The authors declare that the research was conducted in the absence of any commercial or financial relationships that could be construed as a potential conflict of interest.
