# Supplementary material for: Surfaceome and Exoproteome Dynamics in Dual-Species Pseudomonas aeruginosa and Staphylococcus aureus Biofilms
Source: Front Microbiol. 2021 Jun 25;12:672975. doi: 10.3389/fmicb.2021.672975 (PMC8267900; doi:10.3389/fmicb.2021.672975)
Supplement: Supplementary file 1 [file Presentation_1.pdf]

Supplementary Material:

Surfaceome and exoproteome dynamics in dual-species *Pseudomonas aeruginosa* and *Staphylococcus aureus* biofilms

Inés Reigada[1†*], Paola San-Martin-Galindo[1†*], Shella Gilbert-Girard[1], Jacopo Chiaro[1], Vincenzo Cerullo[1], Kirsi Savijoki [1], Tuula A. Nyman[2], Adyary Fallarero[1] and Ilkka Miettinen[1]

[1]Drug Research Program, Division of Pharmaceutical Biosciences, Faculty of Pharmacy, University of Helsinki, Finland.

[2]Department of Immunology, Institute of Clinical Medicine, University of Oslo and Rikshospitalet Oslo, 0372 Oslo, Norway

[†] **These authors have contributed equally to this work and share first authorship.**

*** Correspondence:**
Inés Reigada/ ines.reigada@helsinki.fi
Paola San-Martin-Galindo/paola.sanmartingalindo@helsinki.fi

1 Supplementary Figures

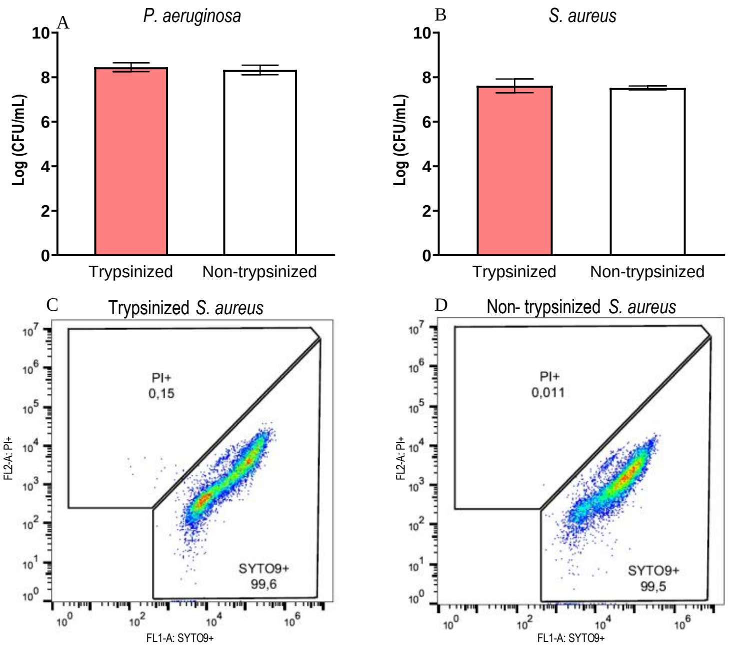

Figure S1. In A-B: Viable colonies recovered from *P. aeruginosa* PAO1 biofilms (A), and *S. aureus* ATCC 25923 biofilms (B) incubated 20 min with or without 55 ng/µl trypsin. The bars show the mean of two individually inoculated and treated biofilms ± SD. In C-D: Representative cytographs corresponding to the viable cells within the *S. aureus* biofilm with (C) and without (D) trypsinization (on the same conditions as in A-B). SYTO 9 was used to stain viable cells while PI was utilized to stain cells with disrupted cell membrane integrity.

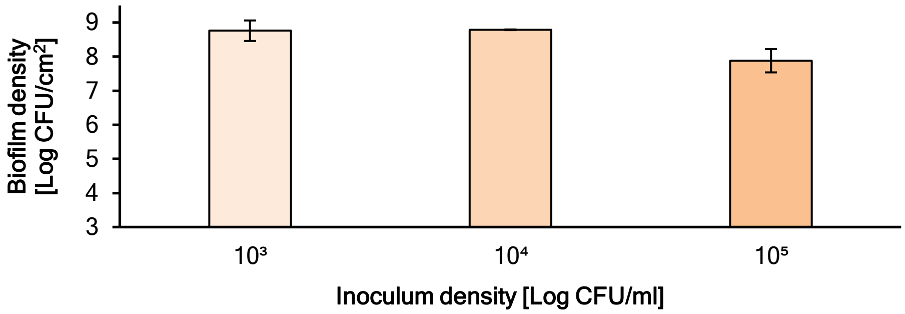

Figure S2. The effect of inoculum density on biofilm viable counts in *P. aeruginosa* monoculture. The bars show the mean of two separately harvested biofilms ± SD.

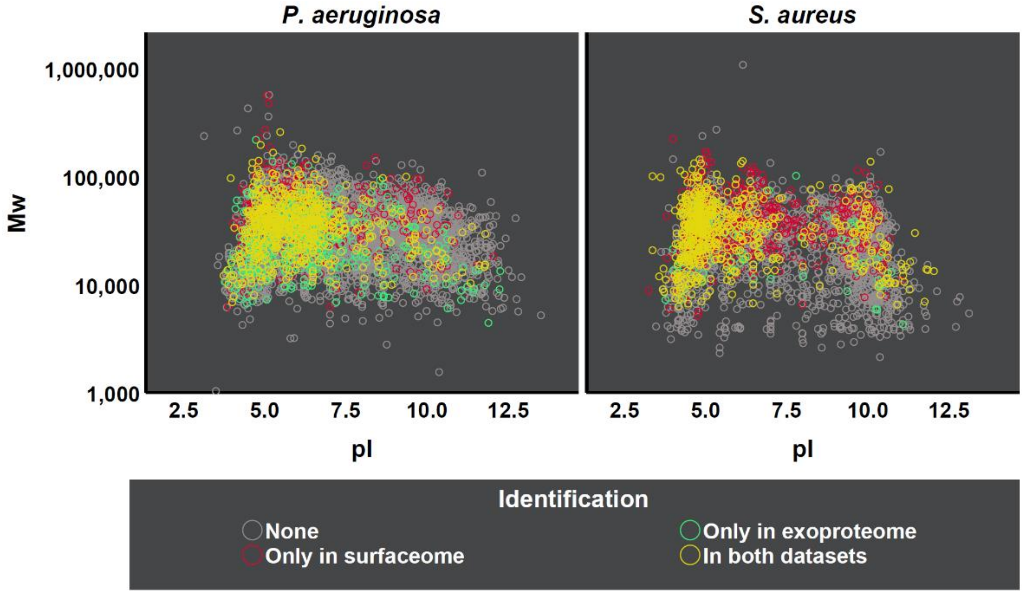

Figure S3. *In silico* 2DE maps illustrating the predicted isoelectric points (pI) and molar weights (Mw) of the identifications. The circle colors indicate the condition in which the protein was identified.

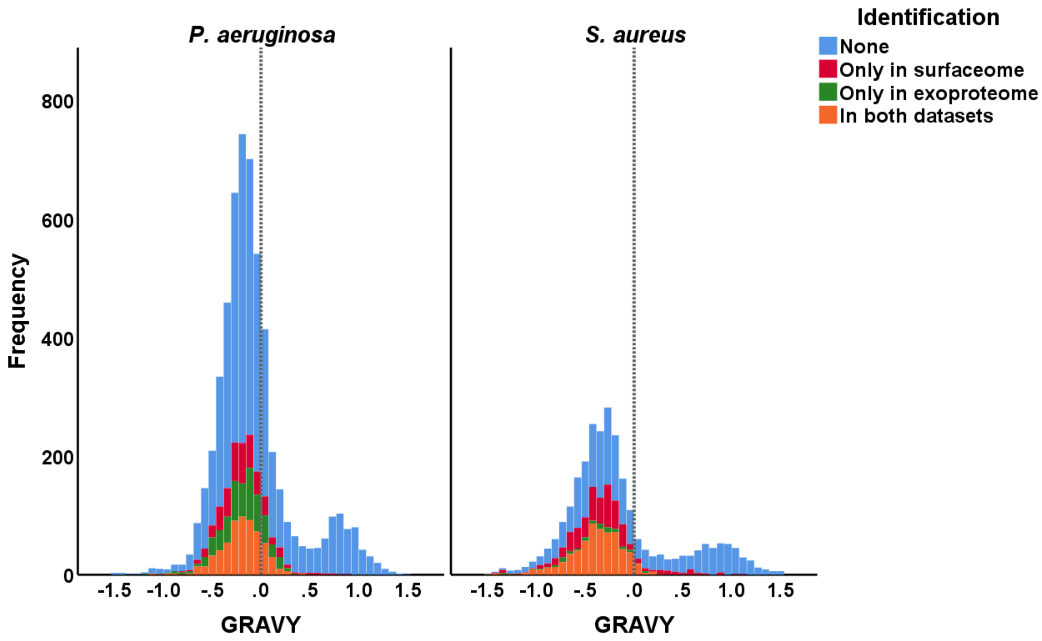

Figure S4. Grand average of hydropathicity (GRAVY) distribution of the mixed species identifications contrasted to the theoretical proteomes. From bottom to top, the stacked histograms represent the proteins identified in both the exoproteome and the surfaceome samples (orange), exclusively in exoproteomes (green), exclusively in surfaceomes (red), or not at all (blue). The vertical reference line separates the negative (hydrophilic) and positive (hydrophobic) values.

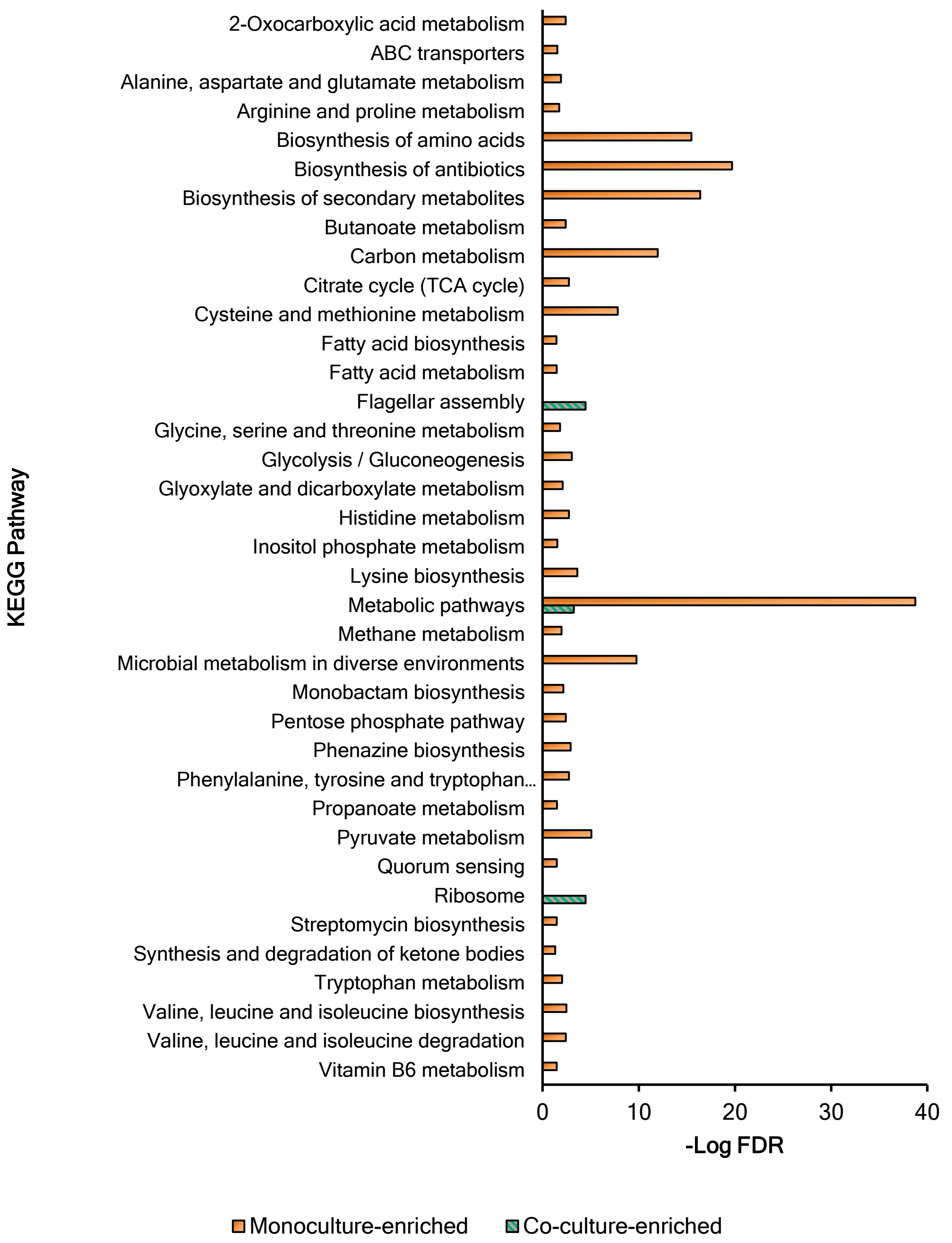

Figure S5. Geneset-based functional enrichment in monocultured vs. co-cultured *P. aeruginosa* biofilm exoproteomes. The statistical assessment of KEGG pathway enrichment was carried out using the STRING database (Szklarczyk et al. 2019). FDR: false discovery rate.

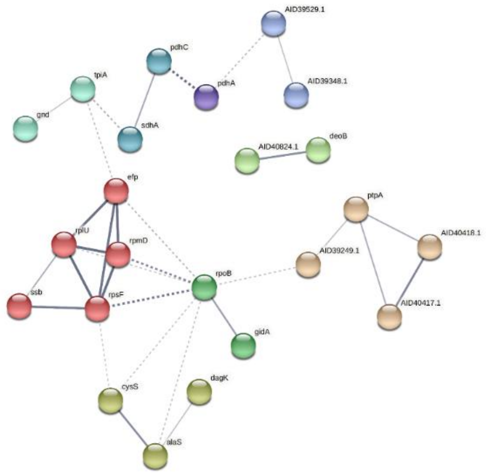

Figure S6. A STRING database (Szklarczyk et al., 2019) network of interacting proteins with significantly higher abundances in *S. aureus* co-culture versus monoculture exoproteome.

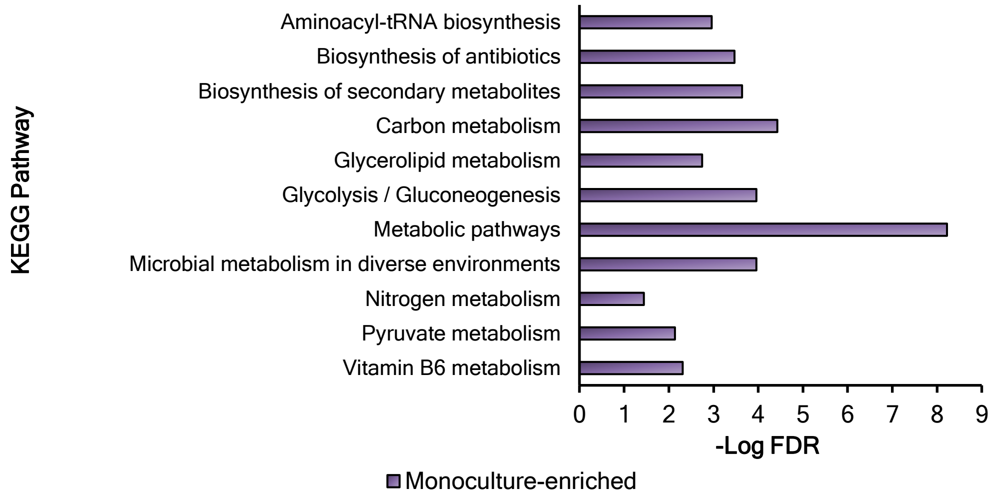

Figure S7. Geneset-based functional enrichment in monocultured vs co-cultured *S. aureus* biofilms exoproteome. The statistical assessment of KEGG pathway enrichment was carried out using the STRING database (Szklarczyk et al. 2019). FDR: false discovery rate.

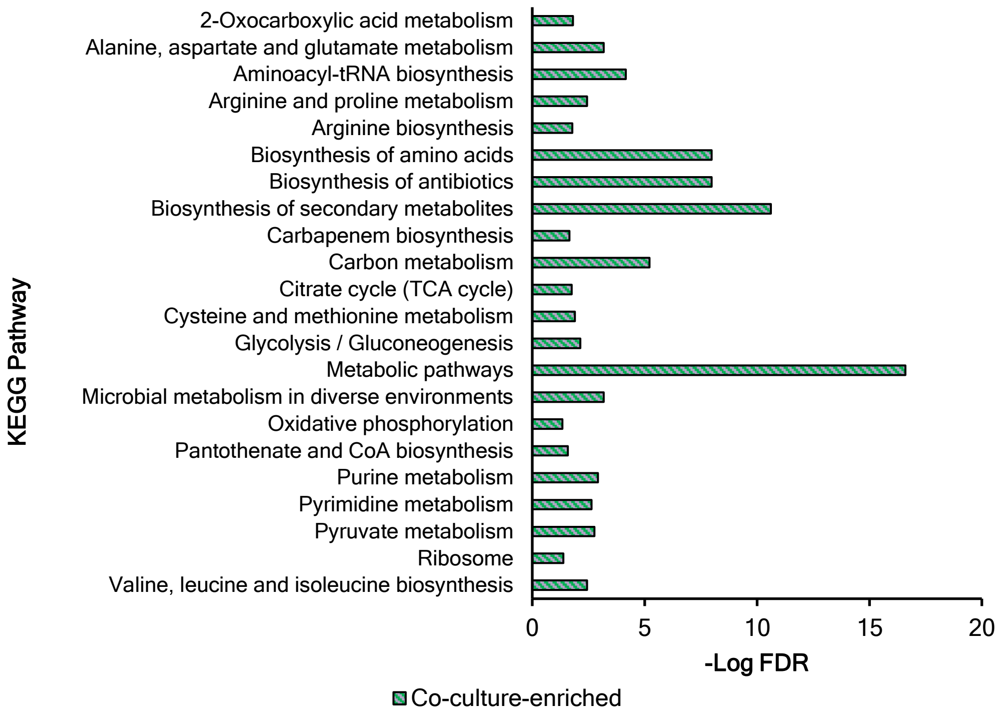

Figure S8. Geneset-based functional enrichment in monocultured vs. co-cultured *P. aeruginosa* biofilm surfaceomes. The statistical assessment of KEGG pathway enrichment was carried out using the STRING database (Szklarczyk et al. 2019).

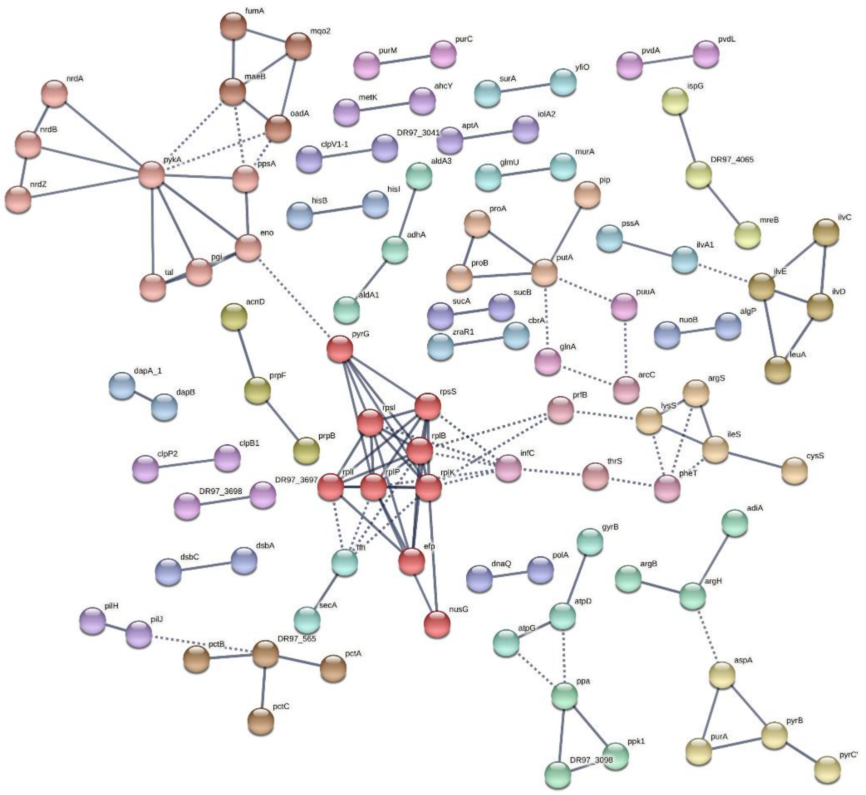

Figure S9. A STRING database (Szklarczyk et al., 2019) network of interacting proteins with significantly higher abundances in *P. aeruginosa* co-culture versus monoculture surfaceome.

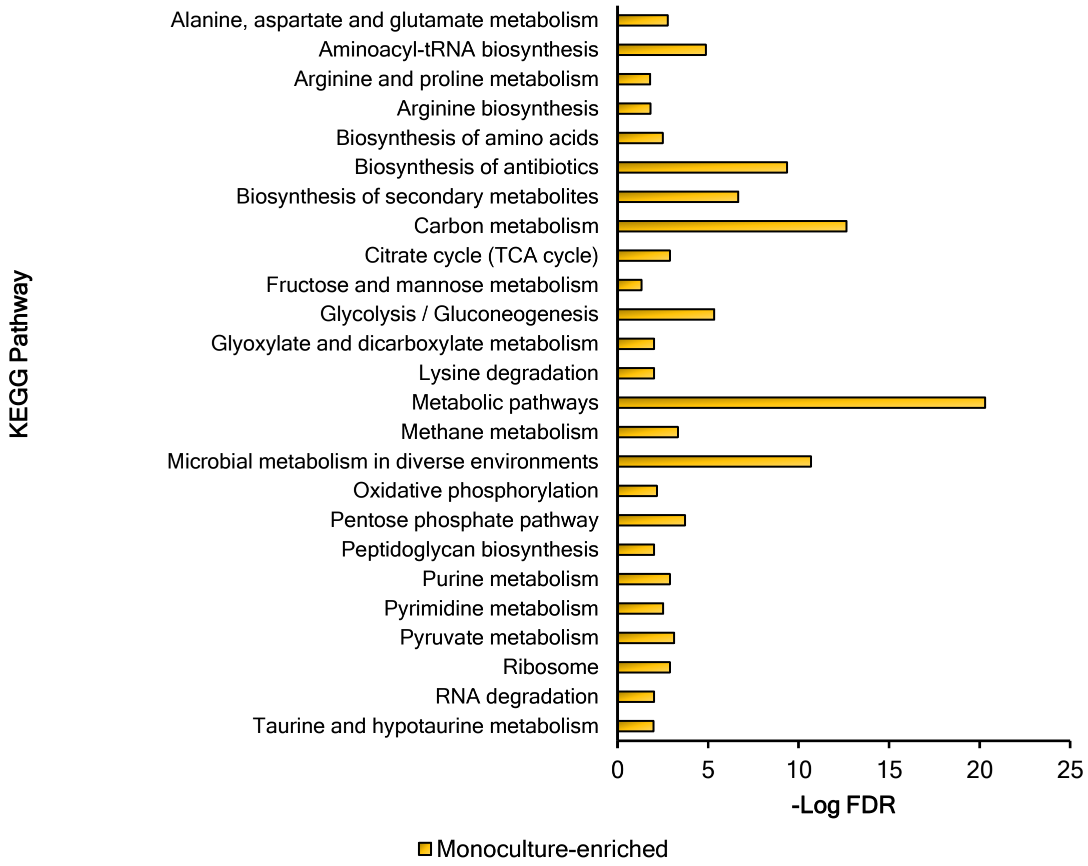

Figure S10. Geneset-based functional enrichment in monocultured vs. co-cultured *S. aureus* biofilm surfaceomes. The statistical assessment of KEGG pathway enrichment was carried out using the STRING database (Szklarczyk et al. 2019).

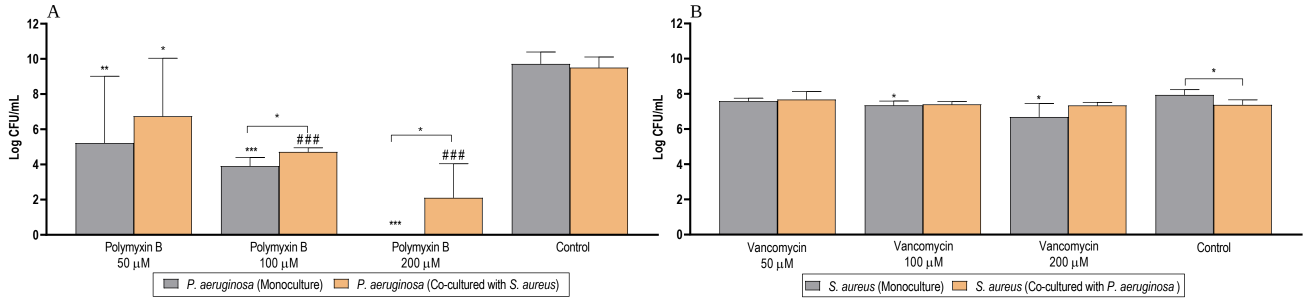

Figure S11. Viable counts of *P. aeruginosa* PAO1 (A) and *S. aureus* ATCC 25923 (B) biofilms after the treatment with different concentrations (50, 100 and µM) of polymyxin B (A) and vancomycin (B). Grey bars shows the viable counts (Log CFU/mL) of treated biofilms formed in monoculture, while orange bars presents corresponding data on biofilms formed in co-culture. "*" indicates statistical difference to the control in monoculture (Student's *t*-test; * $p < 0.05$, **$p<0.01$;*** $p < 0.001$), while "#" represents statistical difference to the control in co-culture (Student's *t*-test; ### $p < 0.001$). Results are expressed as the mean of three or more biological repetitions with their SD.

2 Supplementary Tables

Table S1. Cross-tabulation analysis of the association between predicted secretion mode* and identification in the exoproteome or the surfaceome. The signal peptide-guided secretion pathways include the general secretory pathway (Sec), the twin-arginine translocation pathway (Tat) and the lipoprotein secretory pathway (Lipo). Non-classical secretion is additionally considered. The rows in the table represent the observed and expected counts of proteins in each category. Each subscript letter denotes a subset of secretion categories whose column proportions do not differ significantly from each other at the *p* 0.05 level. *Predicted secretion modes were obtained from SignalP 5.0 and SecretomeP 2.0 (Bendtsen et al., 2005;Almagro Armenteros et al., 2019)

				**Secretion**				
				**None predicted**	**SP**	**TAT**	**LIPO**	**Non-classical**
**Identified in the exoproteomes?**	***P. aeruginosa***	Not identified	Count	3465_{a}	493_{b}	$49_{a, b}$	206_{a}	211_{b}
			Exp. count	3413	527	52	195	237
		Identified	Count	819_{a}	168_{b}	$16_{a, b}$	39_{a}	87_{b}
			Exp. count	871	134	13	50	61
		Total count		4284	661	65	245	298
	S. aureus	Not identified	Count	$944_{a, c}$	90_{c}	$<5_{a, b, c}$	$61_{a, b}$	852_{b}
			Exp. count	1056	108	n < 5	56	727
		Identified	Count	$487_{a, c}$	56_{c}	$< 5_{a, b, c}$	$15_{a, b}$	133_{b}
			Exp. count	375	38	n < 5	20	258
		Total count		1431	146	< 5	76	985
**Identified in the surfaceomes?**	***P. aeruginosa***	Not identified	Count	3451_{a}	545_{a}	55_{a}	207_{a}	234_{a}
			Exp. count	3466	535	53	198	241
		Identified	Count	833_{a}	116_{a}	10_{a}	38_{a}	64_{a}
			Exp. count	819	126	12	47	57
		Total count		4284	661	65	245	298
	S. aureus	Not identified	Count	663_{b}	$96_{a, c}$	$< 5_{a, b, c}$	$44_{a, b}$	735_{c}
			Exp. count	835	85	n < 5	44	574
		Identified	Count	768_{b}	$50_{a, c}$	$< 5_{a, b, c}$	$32_{a, b}$	250_{c}
			Exp. count	597	61	n < 5	32	411
		Total count		1431	146	< 5	76	985

Table S2. The 10 most abundant protein species in *P. aeruginosa* mono- and co-culture exoproteomes. For the Gene Ontology (GO) annotations (The Gene Ontology Consortium 2017), only singe function and cellular component are displayed where several are available. Predicted secretion modes* include the general secretory pathway (Sec), the twin-arginine translocation pathway (Tat), the lipoprotein secretory pathway (Lipo) and non-classical (NC) secretion (O: none predicted). *Predicted secretion modes were obtained from SignalP 5.0 and SecretomeP 2.0 (Bendtsen et al., 2005;Almagro Armenteros et al., 2019).

	Entry name	Function	Gene	GO biological process	GO cellular component	Secretion
***P. aeruginosa* monoculture exoproteome**	ELAS_PSEAE	Elastase LasB	lasB	bacterial-type flagellum-dependent swarming motility	extracellular region	Sec
	Q9HY81_PSEAE	Probable peroxidase	PA3529	cell redox homeostasis	cytosol	O
	ETFB_PSEAE	Electron transfer flavoprotein subunit beta	etfB		cytosol	NC
	Q9HUD3_PSEAE	Malic enzyme	PA5046			O
	DLDH2_PSEAE	Dihydrolipoyl dehydrogenase	lpdG	cell redox homeostasis	cytoplasm	O
	AZUR_PSEAE	Azurin	azu	oxidation-reduction process	periplasmic space	Sec
	SUCD_PSEAE	Succinate--CoA ligase [ADP-forming] subunit alpha	sucD	nucleoside triphosphate biosynthetic process	cytosol	O
	BRAC_PSEAE	Leucine-, isoleucine-, valine-, threonine-, and alanine-binding protein	braC	branched-chain amino acid transport	periplasmic space	Sec
	ETFA_PSEAE	Electron transfer flavoprotein subunit alpha	etfA	fatty acid beta-oxidation using acyl-CoA dehydrogenase		NC
	G3XD47_PSEAE	Arginine/ornithine binding protein AotJ	aotJ	L-arginine import across plasma membrane	extracellular space	Sec
***P. aeruginosa* co-culture exoproteome**	ELAS_PSEAE	Elastase LasB	lasB	bacterial-type flagellum-dependent swarming motility	extracellular region	Sec
	FLID2_PSEAE	B-type flagellar hook-associated protein 2	fliD	bacterial-type flagellum-dependent cell motility	bacterial-type flagellum filament cap	NC
	AZUR_PSEAE	Azurin	azu	oxidation-reduction process	periplasmic space	Sec
	FLICB_PSEAE	B-type flagellin	fliC	bacterial-type flagellum-dependent cell motility	bacterial-type flagellum filament	NC
	LASA_PSEAE	Staphylolytic protease LasA	lasA	pathogenesis	extracellular space	Sec
	Q9HY81_PSEAE	Probable peroxidase	PA3529	cell redox homeostasis	cytosol	O
	G3XD39_PSEAE	Probable bacteriophage protein	PA0622			NC
	Q9I5S9_PSEAE	Probable bacteriophage protein	PA0623			NC
	Q9I4N7_PSEAE	Flagellar protein FlaG	PA1093			NC
	SUCD_PSEAE	Succinate--CoA ligase [ADP-forming] subunit alpha	sucD	nucleoside triphosphate biosynthetic process	cytosol	O

Table S3. The 10 most abundant protein species in *S. aureus* mono- and co-culture exoproteomes. For the Gene Ontology (GO) annotations (The Gene Ontology Consortium 2017), only singe function and cellular component are displayed where several are available. Predicted secretion modes include the general secretory pathway (Sec), the twin-arginine translocation pathway (Tat), the lipoprotein secretory pathway (Lipo) and non-classical (NC) secretion (O: none available). *Predicted secretion modes were obtained from SignalP 5.0 and SecretomeP 2.0 (Bendtsen et al., 2005;Almagro Armenteros et al., 2019).

	Entry name	Function	Gene	GO biological process	GO cellular component	Secretion
***S. aureus* monoculture exoproteome**	WP_000129413.1	arginine deiminase	arcA	arginine catabolic process to ornithine	cytoplasm	O
	WP_000136159.1	ornithine carbamoyltransferase ArcB	arcB	arginine biosynthetic process	cytoplasm	O
	WP_000751265.1	transglycosylase IsaA	isaA	metabolic process	extracellular region	Sec
	WP_000279414.1	glyceraldehyde-3-phosphate dehydrogenase 1	gapA	glycolytic process	cell wall	O
	WP_000634175.1	universal stress protein UspA	uspA_2	response to stress	cytoplasm	O
	WP_001074521.1	bifunctional autolysin Atl	atl	peptidoglycan catabolic process		Sec
	WP_000068176.1	pyruvate dehydrogenase E1 component subunit beta	pdhB	glycolytic process		O
	WP_000792567.1	immunoglobulin-binding protein sbi	sbi	pathogenesis	extracellular region	Sec
	WP_000481438.1	transketolase	tkt	pentose-phosphate shunt		NC
	WP_001031407.1	class I fructose-bisphosphate aldolase	fda	glycolytic process		O
***S. aureus* co-culture exoproteome**	WP_000136159.1	ornithine carbamoyltransferase ArcB	arcB	arginine biosynthetic process	cytoplasm	O
	WP_000129413.1	arginine deiminase	arcA	arginine catabolic process to ornithine	cytoplasm	O
	WP_000279414.1	glyceraldehyde-3-phosphate dehydrogenase 1	gapA	NADH regeneration	cell wall	O
	WP_000034716.1	molecular chaperone DnaK	dnaK	chaperone cofactor-dependent protein refolding	cytoplasm	NC
	WP_001131841.1	fructose-bisphosphate aldolase	fba	glycolytic process		O
	WP_001261460.1	30S ribosomal protein S6	rpsF	translation	ribosome	O
	WP_000481438.1	transketolase	tkt	pentose-phosphate shunt		NC
	WP_001074521.1	bifunctional autolysin Atl	atl	peptidoglycan catabolic process		Sec
	WP_000634175.1	universal stress protein UspA	uspA_2	response to stress	cytoplasm	O
	WP_001574556.1	Thermonuclease Nuc	nuc	pathogenesis	integral component of membrane	Sec

Table S4. The 10 most abundant protein species in *P. aeruginosa* mono- and co-culture surfaceomes. For the Gene Ontology (GO) annotations (The Gene Ontology Consortium 2017), only singe function and cellular component are displayed where several are available. Predicted secretion modes include the general secretory pathway (Sec), the twin-arginine translocation pathway (Tat), the lipoprotein secretory pathway (Lipo) and non-classical (NC) secretion (O: none available). *Predicted secretion modes were obtained from SignalP 5.0 and SecretomeP 2. (Bendtsen et al., 2005;Almagro Armenteros et al., 2019).

	Entry name	Function	Gene	GO biological process	GO cellular component	Secretion
***P. aeruginosa* monoculture surfaceome**	Q9HUC3_PSEAE	Polyhydroxyalkanoate synthesis protein PhaF	phaF			NC
	Q9I4Z7_PSEAE	DNA-binding protein from starved cells Dps	PA0962	cellular iron ion homeostasis	cell	O
	Q9I031_PSEAE	Probable ATP-binding component of ABC transporter	PA2812			O
	Q9HUY5_PSEAE	Mg(2+) transport ATPase, P-type 2	mgtA		integral component of plasma membrane	O
	Q9I5U9_PSEAE	PrkA AAA domain protein	PA0588			O
	Q9I2R2_PSEAE	Probable oxidoreductase	PA1833			O
	MASZ_PSEAE	Malate synthase G	glcB	glyoxylate catabolic process	cytosol	O
	G3XD52_PSEAE	(R)-specific enoyl-CoA hydratase	PA3302	fatty acid biosynthetic process	fatty acid synthase complex	O
	PHZM_PSEAE	Phenazine-1-carboxylate N-methyltransferase	phzM			O
	Q9HTL9_PSEAE	Enamine/imine deaminase	PA5339	organonitrogen compound catabolic process	cytosol	O
***P. aeruginosa* co-culture surfaceome**	ATPB_PSEAE	ATP synthase subunit beta	atpD	ATP synthesis coupled proton transport	plasma membrane	O
	RL2_PSEAE	50S ribosomal protein L2	rplB	cytoplasmic translation	cytosolic large ribosomal subunit	NC
	ODO2_PSEAE	Dihydrolipoyllysine-residue succinyltransferase component of 2-oxoglutarate dehydrogenase complex	sucB	L-lysine catabolic process to acetyl-CoA via saccharopine	oxoglutarate dehydrogenase complex	O
	DSBA_PSEAE	Thiol:disulfide interchange protein DsbA	dsbA	cell redox homeostasis	periplasmic space	Sec
	ALGP_PSEAE	Transcriptional regulatory protein AlgP	algP	alginic acid biosynthetic process		NC
	Q9I4Z7_PSEAE	DNA-binding protein from starved cells Dps	PA0962	cellular iron ion homeostasis	cell	O
	Q9HX76_PSEAE	Probable DNA binding protein	PA3940		cytosol	O
	G3XD14_PSEAE	RNA polymerase-binding transcription factor DksA	dksA	negative regulation of lipid biosynthetic process	cytoplasm	O
	Q9I4I2_PSEAE	Ribonucleoside-diphosphate reductase subunit beta	nrdB	deoxyribonucleotide biosynthetic process	integral component of membrane	O
	Q9I0H9_PSEAE	Peptidase	PA2659			Sec

Table S5. The 10 most abundant protein species in *S. aureus* mono- and co-culture surfaceomes. For the Gene Ontology (GO) annotations (The Gene Ontology Consortium 2017), only singe function and cellular component are displayed where several are available. Predicted secretion modes include the general secretory pathway (Sec), the twin-arginine translocation pathway (Tat), the lipoprotein secretory pathway (Lipo) and non-classical (NC) secretion (O: none available). *Predicted secretion modes were obtained from SignalP 5.0 and SecretomeP 2.0 (Bendtsen et al., 2005;Almagro Armenteros et al., 2019).

	Entry name	Function	Gene	GO biological process	GO cellular component	Secretion
***S. aureus* monoculture surfaceome**	WP_000825534.1	methionine ABC transporter substrate-binding protein	gmpC			Lipo
	WP_000129413.1	arginine deiminase	arcA	arginine catabolic process to ornithine	cytoplasm	O
	WP_000279414.1	glyceraldehyde-3-phosphate dehydrogenase 1	gapA	NADH regeneration	cell wall	O
	WP_001218603.1	aerobic glycerol-3-phosphate dehydrogenase	glpD	glycerol catabolic process	glycerol-3-phosphate dehydrogenase complex	NC
	WP_001131841.1	fructose-bisphosphate aldolase	fba	glycolytic process		O
	WP_000511135.1	ATP synthase subunit beta	atpD	ATP synthesis coupled proton transport	plasma membrane	O
	WP_000068176.1	pyruvate dehydrogenase E1 component subunit beta	pdhB	glycolytic process		O
	WP_000210828.1	serine/threonine dehydratase	tdcB	L-serine catabolic process		O
	WP_000034716.1	molecular chaperone DnaK	dnaK	cellular response to unfolded protein	cytoplasm	NC
	WP_001043863.1	DNA-binding protein HU	hup	chromosome condensation		O
***S. aureus* co-culture surfaceome**	WP_000863437.1	dihydrolipoyllysine-residue acetyltransferase component of pyruvate dehydrogenase complex	pdhC	glycolytic process		O
	WP_000129413.1	arginine deiminase	arcA	arginine catabolic process to ornithine	cytoplasm	O
	WP_001130051.1	malate:quinone oxidoreductase	mqo	tricarboxylic acid cycle	cytoplasm	O
	WP_000268484.1	30S ribosomal protein S2	rpsB	translation	small ribosomal subunit	O
	WP_000127572.1	trigger factor	tig	cell cycle	cytoplasm	O
	WP_000279414.1	glyceraldehyde-3-phosphate dehydrogenase 1	gapA	NADH regeneration	cell wall	O
	WP_000133953.1	30S ribosomal protein S1	rpsA		ribosome	O
	WP_000858795.1	asparagine--tRNA ligase	asnS	asparaginyl-tRNA aminoacylation	cytoplasm	O
	WP_000457386.1	50S ribosomal protein L21	rplU	translation	ribosome	O
	WP_000035325.1	pyruvate dehydrogenase E1 component subunit alpha	pdhA	glycolytic process		O

Table S6: Differences in *P. aeruginosa* protein abundance (Student's *t*-test, FDR 0.15) in co-culture vs. monoculture exoproteome.

Entry name	Protein function	STRING ID	Log_2 ΔLFQ	q value
Q9I4N7_PSEAE	Flagellar protein FlaG	flaG	5.2	0.0108
G3XCX2_PSEAE	Phage tail assembly protein	DR97_3594	4.8	0.0280
Q9HYP5_PSEAE	FlgM	flgM	4.5	0.0296
Q9I5S9_PSEAE	Probable bacteriophage protein	gpFII	4.5	0.0475
Q9HUD6_PSEAE	Putative secreted protein	DR97_2388	4.3	0.0312
G3XD39_PSEAE	Probable bacteriophage protein	gpFI	3.9	0.0286
Q9I613_PSEAE	Uncharacterized protein	DR97_3473	3.6	0.0279
G3XD68_PSEAE	Phage_TAC_13 domain-containing protein	#N/A	3.6	0.0264
Q9I5F6_PSEAE	Bifunctional protein PutA	putA	3.6	0.0702
HCP1_PSEAE	Protein hcp1	hcp1	3.5	0.0980
Q9HXV6_PSEAE	Uncharacterized protein	DR97_4193	3.5	0.0330
G3XCX5_PSEAE	Probable bacteriophage protein	J	3.4	0.0165
RECA_PSEAE	Protein RecA (Recombinase A)	recA	3.4	0.0335
G3XD38_PSEAE	Putative major tail protein V	#N/A	3.2	0.1283
ODO2_PSEAE	Dihydrolipoamide succinyltransferase component of 2-oxoglutarate dehydrogenase complex	sucB	3.1	0.0303
RL2_PSEAE	50S ribosomal protein L2	rplB	2.9	0.0381
Q9HUA1_PSEAE	Probable binding protein component of ABC transporter	gltI1	2.9	0.0114
G3XD71_PSEAE	Probable bacteriophage protein	DR97_3590	2.9	0.0295
FLID2_PSEAE	B-type flagellar hook-associated protein 2	fliD	2.9	0.0412
Q9I4P3_PSEAE	Flagellar hook-associated protein 1 FlgK	flgK	2.8	0.0524
Q9I130_PSEAE	Putative ferric enterobactin esterase	DR97_5981	2.8	0.0298
G3XDA6_PSEAE	Repressor, PtrB	DR97_3582	2.6	0.0272
FLICB_PSEAE	B-type flagellin	fliC	2.6	0.1034
Q9I4P2_PSEAE	Flagellar hook-associated protein type 3 FlgL	flgL	2.6	0.0938
PILY1_PSEAE	Type IV pilus biogenesis factor PilY1	pilY1	2.6	0.0901
Q9I3D3_PSEAE	2-oxoglutarate dehydrogenase (E1 subunit)	sucA	2.5	0.0394
Q9I5D1_PSEAE	AmpDh3	amiD_2	2.5	0.0000
Q9I129_PSEAE	Lipoprotein	DR97_5980	2.5	0.0987
LASA_PSEAE	Staphylolytic protease LasA	lasA	2.4	0.0458
CARB_PSEAE	Carbamoyl-phosphate synthase large chain	carB	2.3	0.0155
RL20_PSEAE	50S ribosomal protein L20	rplT	2.3	0.0686
RL5_PSEAE	50S ribosomal protein L5	rplE	2.3	0.0806
Q9HYZ6_PSEAE	Site-determining protein	minD	2.2	0.0287
G3XD55_PSEAE	Type 4 fimbrial biogenesis protein PilY2	pilY2	2.2	0.0311
RL31_PSEAE	50S ribosomal protein L31	DR97_2404	2.1	0.1141
RL29_PSEAE	50S ribosomal protein L29	rpmC	2.1	0.0991
RS8_PSEAE	30S ribosomal protein S8	rpsH	2.0	0.1443
Q9I131_PSEAE	Uncharacterized protein	DR97_5981	2.0	0.0299
Q9I4I1_PSEAE	Ribonucleoside-diphosphate reductase	nrdA	1.9	0.0829
Q9I3Q6_PSEAE	Flagellar hook-length control protein FliK	DR97_582	1.8	0.0373
RHO_PSEAE	Transcription termination factor Rho	rho	1.8	0.1433
RISB_PSEAE	6,7-dimethyl-8-ribityllumazine synthase	ribH	1.7	0.0165
Q9HYU8_PSEAE	Probable HIT family protein	DR97_4635	1.7	0.0162
Q9I4P9_PSEAE	Flagellar hook protein FlgE	flgE	1.7	0.0332
ATPD_PSEAE	ATP synthase subunit delta	atpH	1.7	0.0721
Q9HXM5_PSEAE	Inosine-5'-monophosphate dehydrogenase	guaB	1.6	0.0456
KDSB_PSEAE	3-deoxy-manno-octulosonate cytidylyltransferase	kdsB	1.5	0.0282
Q9HZM8_PSEAE	Ribonuclease E	rne	1.5	0.0218
Q9HYJ2_PSEAE	DNA damage-inducible protein YebG	DR97_4509	1.5	0.0419
GLN1B_PSEAE	Glutamine synthetase	glnA	1.5	0.0321
ATPE_PSEAE	ATP synthase epsilon chain	atpC	1.5	0.0333
Q9HW32_PSEAE	Insulin-cleaving metalloproteinase outer membrane protein	icmP	1.5	0.0387
Q9I4I2_PSEAE	Ribonucleoside-diphosphate reductase subunit beta	nrdB	1.4	0.1165
RS16_PSEAE	30S ribosomal protein S16	rpsP	1.3	0.0997
RL4_PSEAE	50S ribosomal protein L4	rplD	1.3	0.0282
Q9I5S8_PSEAE	Probable bacteriophage protein	#N/A	1.3	0.0746
Q9HW15_PSEAE	Probable short-chain dehydrogenase	speA3	1.2	0.1037
Q9HV16_PSEAE	Glycerophosphodiester phosphodiesterase	DR97_2136	1.2	0.0705
MAO1_PSEAE	NAD-dependent malic enzyme	maeA	1.2	0.0209
Q9I5E2_PSEAE	2-methylisocitrate lyase	prpB	1.2	0.0377
G3XD20_PSEAE	Periplasmic serine endoprotease DegP-like	mucD	1.2	0.1240
ALR2_PSEAE	Alanine racemase, catabolic	dadX	1.2	0.0269
Q9I5E4_PSEAE	Probable aconitate hydratase	acnD	1.1	0.0980
ODBB_PSEAE	2-oxoisovalerate dehydrogenase subunit beta	bkdA2	1.1	0.0300
STHA_PSEAE	Soluble pyridine nucleotide transhydrogenase	sthA	1.1	0.0373
ILVC_PSEAE	Ketol-acid reductoisomerase (NADP(+))	ilvC	1.0	0.0267
ATPB_PSEAE	ATP synthase subunit beta	atpD	1.0	0.0397
Q9HX39_PSEAE	Uncharacterized protein conserved in bacteria	DR97_3886	1.0	0.0309
HLDE_PSEAE	Bifunctional protein HldE	hldE	1.0	0.0696
Q9I6H5_PSEAE	D-3-phosphoglycerate dehydrogenase	serA	0.9	0.0842
Q9I5V9_PSEAE	Glutamyl-tRNA amidotransferase	DR97_3547	0.9	0.1490
LAP_PSEAE	Leucine aminopeptidase	DR97_5001	0.8	0.0431
RIBB_PSEAE	3,4-dihydroxy-2-butanone 4-phosphate synthase	ribB	0.8	0.1323
HIS7_PSEAE	Imidazoleglycerol-phosphate dehydratase	hisB	0.8	0.1295
DADA1_PSEAE	D-amino acid dehydrogenase 1	dadA1	0.8	0.0293
Y3453_PSEAE	UPF0502 protein PA3453	DR97_4468	0.6	0.1078

Entry name	Protein function	STRING ID	Log_2 ΔLFQ	q value
FABV_PSEAE	Enoyl-[acyl-carrier-protein] reductase [NADH]	fabV	0.5	0.1299
Q9HY50_PSEAE	Zinc-type alcohol dehydrogenase-like protein	DR97_4372	0.5	0.1483
Q9HVA8_PSEAE	Ferric iron-binding periplasmic protein HitA	fbpA	0.4	0.1293
SYM_PSEAE	Methionine--tRNA ligase	metG	0.4	0.1426
Q9HT52_PSEAE	Probable short-chain dehydrogenase	tsaC1	-0.5	0.1152
PQSE_PSEAE	2-aminobenzoylacetyl-CoA thioesterase	dfa3	-0.5	0.1297
DAPD_PSEAE	2,3,4,5-tetrahydropyridine-2,6-dicarboxylate N-succinyltransferase	dapD	-0.6	0.1318
Q9HW72_PSEAE	Pyruvate kinase	pykA	-0.6	0.0135
DHOM_PSEAE	Homoserine dehydrogenase	hom	-0.6	0.0830
Y1579_PSEAE	Uncharacterized protein PA1579	DR97_355	-0.6	0.0806
Q9HVA1_PSEAE	Acetolactate synthase isozyme III small subunit	ilvN	-0.6	0.0366
MURA_PSEAE	UDP-N-acetylglucosamine 1-carboxyvinyltransferase	murA	-0.6	0.1058
T23O_PSEAE	Tryptophan 2,3-dioxygenase	kynA	-0.6	0.1167
SUCD_PSEAE	Succinate--CoA ligase [ADP-forming] subunit alpha	sucD	-0.7	0.0663
Q9I2R7_PSEAE	Probable short-chain dehydrogenase	fabG_17	-0.7	0.0369
Q9HX51_PSEAE	Probable transcriptional regulator	DR97_3901	-0.7	0.1317
Q9HXR8_PSEAE	Probable FMN oxidoreductase	namA	-0.7	0.0560
P5217_PSEAE	Probable binding protein component of ABC iron transporter PA5217	DR97_2586	-0.7	0.0664
Q9I3W1_PSEAE	Putative secreted protein	DR97_640	-0.7	0.0309
Q9HW87_PSEAE	Formyltetrahydrofolate deformylase	purU	-0.7	0.1290
ASTD_PSEAE	N-succinylglutamate 5-semialdehyde dehydrogenase	astD	-0.8	0.0284
Q9I2W9_PSEAE	Phosphoenolpyruvate synthase	ppsA	-0.8	0.0501
Q9I0T2_PSEAE	Probable acyl-CoA dehydrogenase	mmgC3	-0.8	0.1428
Q9HT66_PSEAE	Cysteine hydrolase	DR97_2885	-0.8	0.0270
ALR1_PSEAE	Alanine racemase, biosynthetic	DR97_2281	-0.8	0.0775
Q9HYR6_PSEAE	Condensation domain protein	DR97_4598	-0.8	0.1323
GLYA2_PSEAE	Serine hydroxymethyltransferase 2	glyA3	-0.8	0.1486
Q9HTD1_PSEAE	Probable transcarboxylase subunit	oadA	-0.9	0.0666
Q9I067_PSEAE	Putative oxidoreductase	puuB1	-0.9	0.1421
SYV_PSEAE	Valine--tRNA ligase	valS	-0.9	0.0588
NUOCD_PSEAE	NADH-quinone oxidoreductase subunit C/D	nuoD	-0.9	0.0994
G3XDA5_PSEAE	Anaerobically-induced outer membrane porin OprE	oprE	-0.9	0.0424
PCKA_PSEAE	Phosphoenolpyruvate carboxykinase (ATP)	pckA	-0.9	0.0395
NADA_PSEAE	Quinolinate synthase A	nadA	-0.9	0.1403
AROB_PSEAE	3-dehydroquinate synthase	aroB	-0.9	0.1020
FABA_PSEAE	3-hydroxydecanoyl-[acyl-carrier-protein] dehydratase FabA	fabA	-0.9	0.0691
Q9I4Z7_PSEAE	Probable dna-binding stress protein	dps2	-0.9	0.0300
LEU1_PSEAE	2-isopropylmalate synthase	leuA	-1.0	0.0403
RECR_PSEAE	Recombination protein RecR	recR	-1.0	0.1000
Q9I1S0_PSEAE	NADP-dependent oxidoreductase	curA	-1.0	0.0966
AAT_PSEAE	Aspartate aminotransferase (AspAT)	aspC	-1.0	0.0526
Q9HZH0_PSEAE	Probable porin	opdQ	-1.0	0.0624
Y329_PSEAE	UPF0339 protein PA0329	DR97_3294	-1.0	0.0450
AROE_PSEAE	Shikimate dehydrogenase (NADP(+))	aroE	-1.0	0.1492
Q9HVV9_PSEAE	3-deoxy-D-manno-octulosonate 8-phosphate phosphatase KdsC	DR97_1637	-1.0	0.1164
Y3808_PSEAE	Uncharacterized protein PA3808	iscX	-1.0	0.1198
Q9HXG8_PSEAE	ABC transporter substrate-binding protein	DR97_4030	-1.0	0.0267
METH_PSEAE	Methionine synthase	metH	-1.0	0.0410
GLO2_PSEAE	Hydroxyacylglutathione hydrolase (Glyoxalase II)	gloB	-1.0	0.0951
Q9HY81_PSEAE	Probable peroxidase	tsaA	-1.0	0.0304
Q9I5A7_PSEAE	Inner membrane lipoprotein YiaD	yiaD_1	-1.0	0.0473
Q9I5A8_PSEAE	Beta-lactamase	ycbL	-1.0	0.1490
DAPB_PSEAE	4-hydroxy-tetrahydrodipicolinate reductase	dapB	-1.0	0.0325
Q9HZ15_PSEAE	Probable transcriptional regulator	DR97_4710	-1.1	0.0602
Q9I2F4_PSEAE	Ribokinase	rbsK	-1.1	0.0689
Q9I083_PSEAE	Probable outer membrane protein	oprD3	-1.1	0.0566
HCNB_PSEAE	Hydrogen cyanide synthase subunit HcnB	DR97_6236	-1.1	0.0730
Q9I348_PSEAE	Cysteine hydrolase	DR97_211	-1.1	0.0301
PGK_PSEAE	Phosphoglycerate kinase	pgk	-1.1	0.0411
ARGC_PSEAE	N-acetyl-gamma-glutamyl-phosphate reductase	argC	-1.1	0.0285
ETFB_PSEAE	Electron transfer flavoprotein subunit beta	etfB	-1.1	0.0364
ASPQ_PSEAE	Glutaminase-asparaginase	ansB	-1.1	0.0345
TPIS_PSEAE	Triosephosphate isomerase	tpiA	-1.1	0.1212
Q9HVW4_PSEAE	ABC transporter periplasmic binding protein MlaC	ttg2D	-1.1	0.1320
Q9I746_PSEAE	Putative secretion protein	DR97_3043	-1.1	0.1021
Q9I765_PSEAE	Oligopeptidase A	prlC	-1.1	0.0278
Q9HVS3_PSEAE	Probable metallopeptidase	pepQ	-1.2	0.1316
Q9HXY0_PSEAE	Probable aminotransferase	dapC	-1.2	0.0405
ETFA_PSEAE	Electron transfer flavoprotein subunit alpha	etfA	-1.2	0.0126
DAVT_PSEAE	5-aminovalerate aminotransferase DavT	gabT	-1.2	0.0325
OADC_PSEAE	Oxaloacetate decarboxylase	bcpA	-1.2	0.0077
GSHB_PSEAE	Glutathione synthetase	gshB	-1.2	0.0311
Q9HVU6_PSEAE	(R)-stereoselective amidase	DR97_1654	-1.2	0.0582
DLDH2_PSEAE	Dihydrolipoyl dehydrogenase	lpd	-1.2	0.0291
Q9I3L9_PSEAE	Sulfate-binding protein of ABC transporter	sbp3	-1.2	0.0379
ILVE_PSEAE	Branched-chain-amino-acid aminotransferase	ilvE	-1.2	0.0434
Q9I4C2_PSEAE	Class I SAM-dependent methyltransferase	DR97_718	-1.2	0.0758
Q9HW68_PSEAE	Fumarate hydratase class I	fumA	-1.2	0.0692
SPUD_PSEAE	Putrescine-binding periplasmic protein SpuD	potF1	-1.2	0.0274
Q14T74_PSEAE	Putative NAD(P) transhydrogenase, subunit alpha part 1	pntAA	-1.2	0.0281

Entry name	Protein function	STRING ID	Log_2 ΔLFQ	q value
Q9HUD3_PSEAE	Malic enzyme	maeB	-1.2	0.0302
AROQ1_PSEAE	3-dehydroquinate dehydratase 1	aroQ1	-1.2	0.0300
PDXA_PSEAE	4-hydroxythreonine-4-phosphate dehydrogenase	pdxA	-1.3	0.0778
Q9HTM3_PSEAE	UPF0701 protein	yicC	-1.3	0.1438
Q9HZH7_PSEAE	Molybdenum cofactor biosynthesis protein B	moaB_1	-1.3	0.1247
HUTI_PSEAE	Imidazolonepropionase	hutI	-1.3	0.0789
GCH1L_PSEAE	GTP cyclohydrolase 1 type 2 homolog	DR97_1622	-1.3	0.0277
Q9HU31_PSEAE	Amino acid (Lysine/arginine/ornithine/histidine/octopine) ABC transporter periplasmic binding protein	artJ	-1.3	0.0281
Q9HZI9_PSEAE	Universal stress protein	DR97_4920	-1.3	0.0120
THRC_PSEAE	Threonine synthase	thrC	-1.3	0.1385
Q9HWZ2_PSEAE	FecR protein	DR97_3832	-1.3	0.1296
G3XD47_PSEAE	Arginine/ornithine binding protein AotJ	argT3	-1.3	0.0129
Q9HY51_PSEAE	Antibiotic biosynthesis monooxygenase	ycnE	-1.3	0.0285
Q9I4S7_PSEAE	Probable esterase	DR97_897	-1.3	0.0876
HISZ_PSEAE	ATP phosphoribosyltransferase regulatory subunit	hisZ	-1.4	0.0950
ILVD_PSEAE	Dihydroxy-acid dehydratase (DAD)	ilvD	-1.4	0.0302
GATB_PSEAE	Aspartyl/glutamyl-tRNA(Asn/Gln) amidotransferase subunit B	gatB	-1.4	0.0991
Q9HY22_PSEAE	Response regulator ErdR	erdR	-1.4	0.1262
Q9HTU9_PSEAE	Glyoxalase	elbB	-1.4	0.0958
Q9I0R1_PSEAE	Nitroreductase	DR97_5594	-1.4	0.0323
Q9I2A6_PSEAE	3-hydroxybutyrate dehydrogenase	bdhA	-1.4	0.1166
CBPD_PSEAE	Chitin-binding protein CbpD (Protease LasD)	cbpD	-1.4	0.1499
GLMU_PSEAE	Bifunctional protein GlmU	glmU	-1.4	0.0295
Q9I6K7_PSEAE	Sulfate-binding protein	sbp1	-1.4	0.0675
GATA_PSEAE	Glutamyl-tRNA(Gln) amidotransferase subunit	gatA	-1.4	0.0708
NADC_PSEAE	Nicotinate-nucleotide pyrophosphorylase [carboxylating]	nadC	-1.4	0.1439
Q9HU22_PSEAE	Glucose-1-phosphate thymidylyltransferase	rfbA	-1.4	0.0984
NQOR_PSEAE	NAD(P)H dehydrogenase (quinone) / Flavoprotein WrbA	wrbA	-1.4	0.0977
HIS3_PSEAE	Phosphoribosyl-AMP cyclohydrolase	hisI	-1.4	0.1421
CATA_PSEAE	Catalase	katA	-1.4	0.0270
PURA_PSEAE	Adenylosuccinate synthetase	purA	-1.4	0.0147
RS18_PSEAE	30S ribosomal protein S18	rpsR	-1.5	0.0407
G3XD52_PSEAE	(R)-specific enoyl-CoA hydratase	phaJ	-1.5	0.0274
Q9HTR6_PSEAE	Nitrogen regulatory protein P-II 2	glnK	-1.5	0.0481
ACKA_PSEAE	Acetate kinase	ackA1	-1.5	0.0980
Q9HY11_PSEAE	Cytochrome D ubiquinol oxidase subunit II	DR97_4324	-1.5	0.1152
Q9HW45_PSEAE	Xenobiotic reductase	nemA3	-1.5	0.0086
TRPB_PSEAE	Tryptophan synthase beta chain	trpB	-1.5	0.0214
G3XDA2_PSEAE	3-oxoacyl-[acyl-carrier-protein] synthase 2	fabF1	-1.5	0.0274
DEF_PSEAE	Peptide deformylase	def_1	-1.5	0.0371
Y3435_PSEAE	Uncharacterized protein PA3435	cinC	-1.5	0.0622
Q9HXR0_PSEAE	Uncharacterized protein	DR97_4146	-1.5	0.0362
DAPA_PSEAE	4-hydroxy-tetrahydrodipicolinate synthase	dapA_1	-1.5	0.0366
PYRE_PSEAE	Orotate phosphoribosyltransferase	pyrE	-1.5	0.0308
CYSNC_PSEAE	Bifunctional enzyme CysN/CysC	cysN/C	-1.5	0.0445
MTIP_PSEAE	S-methyl-5'-thioinosine phosphorylase	mtnP	-1.5	0.0300
SPUE_PSEAE	Spermidine-binding periplasmic protein SpuE	potF3	-1.6	0.0200
Q9HVU7_PSEAE	Metalloprotease TldD	tldD	-1.6	0.0732
LOLA_PSEAE	Outer-membrane lipoprotein carrier protein	lolA	-1.6	0.1266
CHMU_PSEAE	Monofunctional chorismate mutase	DR97_2554	-1.6	0.0283
PROA_PSEAE	Gamma-glutamyl phosphate reductase	proA	-1.6	0.0611
GCH12_PSEAE	GTP cyclohydrolase 1 2	folE3	-1.6	0.0994
Q9I338_PSEAE	LTA synthase family protein	DR97_198	-1.6	0.0436
UBIC_PSEAE	Probable chorismate pyruvate-lyase	ubiC	-1.6	0.0949
Q9I2D5_PSEAE	Lipoprotein	DR97_5879	-1.6	0.0293
SAHH_PSEAE	Adenosylhomocysteinase	ahcY	-1.6	0.0098
Q9I0Z1_PSEAE	Oxidoreductase MexS	DR97_5681	-1.6	0.0460
Q9I296_PSEAE	Putative isovaleryl-CoA dehydrogenase	IVD1	-1.6	0.0294
Y4667_PSEAE	TPR repeat-containing protein PA4667	DR97_1976	-1.6	0.1251
Q9HTN7_PSEAE	Probable binding protein component of ABC dipeptide transporter	dppA1	-1.6	0.0337
Q9I3D5_PSEAE	Succinate dehydrogenase flavoprotein subunit	sdhA	-1.6	0.0218
Q9HX07_PSEAE	Murein peptide ligase	mpl	-1.7	0.0763
Q9I610_PSEAE	Probable acyl-CoA dehydrogenase	DR97_3476	-1.7	0.0321
FUR_PSEAE	Ferric uptake regulation protein	fur	-1.7	0.0222
Q9HXS8_PSEAE	Spermidine dehydrogenase, SpdH	DR97_4164	-1.7	0.0289
Q9I0D3_PSEAE	Cysteine synthase	cysK	-1.7	0.0355
Q9HZ93_PSEAE	Probable aldolase	eda_1	-1.7	0.0299
Y2980_PSEAE	UPF0434 protein PA2980	DR97_4959	-1.7	0.0665
TOLB_PSEAE	Tol-Pal system protein TolB	tolB	-1.7	0.1398
PDXJ_PSEAE	Pyridoxine 5'-phosphate synthase	pdxJ	-1.7	0.0276
BIOB_PSEAE	Biotin synthase	bioB	-1.7	0.0403
METK_PSEAE	S-adenosylmethionine synthase	metK	-1.7	0.0327
AK_PSEAE	Aspartokinase	lysC	-1.7	0.0302
Q9I2R2_PSEAE	Probable oxidoreductase	yhfP	-1.7	0.0148
Q9HWI2_PSEAE	Probable acyl-CoA dehydrogenase	DR97_3476	-1.7	0.0415
QOR_PSEAE	Quinone oxidoreductase	qor	-1.8	0.0667
SUHB_PSEAE	Inositol-1-monophosphatase	suhB	-1.8	0.1402
G3XCZ6_PSEAE	Malonyl CoA-acyl carrier protein transacylase	fabD	-1.8	0.0269
Q9I2C4_PSEAE	NAD+ dependent aldehyde dehydrogenase ExaC	aldA3	-1.8	0.0304
Q9HX05_PSEAE	Probable aldehyde dehydrogenase	aldA1	-1.8	0.0306
Q9HTF4_PSEAE	GbcA	gbcA	-1.8	0.1320

Entry name	Protein function	STRING ID	Log_2 ΔLFQ	q value
ARCA_PSEAE	Arginine deiminase	arcA	-1.8	0.0274
Q9I500_PSEAE	HIT domain-containing protein	DR97_978	-1.8	0.1013
CSRA_PSEAE	Translational regulator CsrA	DR97_1038	-1.8	0.0489
PURE_PSEAE	N5-carboxyaminoimidazole ribonucleotide mutase	purE	-1.8	0.0997
Q9HUP7_PSEAE	Putative ADP-ribose pyrophosphatase	DR97_2267	-1.8	0.0272
Q9I6Q7_PSEAE	Beta-ketoadipate enol-lactone hydrolase	catD	-1.8	0.0298
FADB_PSEAE	Fatty acid oxidation complex subunit alpha	fadB	-1.8	0.1171
Q9HTL9_PSEAE	Enamine/imine deaminase	yabJ_5	-1.8	0.0597
Y3998_PSEAE	UPF0250 protein PA3998	DR97_3869	-1.8	0.0272
LDC_PSEAE	Murein tetrapeptide carboxypeptidase	DR97_2567	-1.9	0.0373
Q9I0T0_PSEAE	Probable short-chain dehydrogenase	linC_1	-1.9	0.0226
Q9I6C3_PSEAE	Insulinase family protein	DR97_3338	-1.9	0.1300
NMO_PSEAE	Nitronate monooxygenase	DR97_920	-1.9	0.0269
6PGL_PSEAE	6-phosphogluconolactonase (6PGL)	pgl	-1.9	0.1264
GLMS_PSEAE	Glutamine--fructose-6-phosphate aminotransferase [isomerizing]	glmS	-1.9	0.0458
Q9I0V4_PSEAE	Metalloprotease TldD	DR97_5642	-1.9	0.0364
Q9I584_PSEAE	Morphogene protein BolA	bolA	-1.9	0.0290
Q9HUQ0_PSEAE	Probable binding protein component of ABC transporter	livJ	-1.9	0.0335
Q9I4W2_PSEAE	Lipoprotein	DR97_936	-1.9	0.0299
Q9I3C4_PSEAE	Dienelactone hydrolase	DR97_292	-1.9	0.0562
GATC_PSEAE	Glutamyl-tRNA(Gln) amidotransferase subunit C	gatC	-2.0	0.1180
Q9HTW6_PSEAE	Aminopeptidase P	pepP	-2.0	0.0778
HGD_PSEAE	Homogentisate 1,2-dioxygenase	hmgA	-2.0	0.0291
TAL_PSEAE	Transaldolase	tal	-2.0	0.1212
Q9I4D6_PSEAE	Probable hydrolase	ycaC	-2.0	0.0520
Q9I3D4_PSEAE	Succinate dehydrogenase (B subunit)	sdhB	-2.0	0.0323
RL28_PSEAE	50S ribosomal protein L28	rpmB	-2.0	0.0673
PYRR_PSEAE	Bifunctional protein PyrR	pyrR	-2.0	0.0375
MURD_PSEAE	UDP-N-acetylmuramoylalanine--D-glutamate ligase	murD	-2.0	0.0669
Q9I766_PSEAE	Gamma carbonic anhydrase family protein	yrdA	-2.0	0.0399
GGT_PSEAE	Glutathione hydrolase proenzyme	DR97_456	-2.0	0.0151
PHHC_PSEAE	Aromatic-amino-acid aminotransferase	phhC	-2.0	0.0140
HISX_PSEAE	Histidinol dehydrogenase	hisD	-2.0	0.0407
GMHA_PSEAE	Phosphoheptose isomerase	gmhA	-2.0	0.0432
Q9I015_PSEAE	Probable aminotransferase	alaA	-2.1	0.1495
Q9HVY1_PSEAE	Probable oxidoreductase	tas	-2.1	0.1268
Q9I5T1_PSEAE	Ribulose-phosphate 3-epimerase	rpe	-2.1	0.0672
Q9HYP8_PSEAE	STAS-domain containing protein PA14_20770	DR97_4580	-2.1	0.0286
Q9HXJ0_PSEAE	Iron-binding protein IscA	iscA	-2.1	0.0274
Q9HXK0_PSEAE	(R)-stereoselective amidase	yafV	-2.1	0.0266
Q9I1R3_PSEAE	Probable binding protein component of ABC transporter	DR97_6226	-2.1	0.0302
Q9HYB3_PSEAE	Leucyl-tRNA synthetase	DR97_4428	-2.1	0.0452
Q9I6W2_PSEAE	Uncharacterized protein PA14_02130	DR97_3126	-2.1	0.0594
Q9I2T8_PSEAE	Peptidylprolyl isomerase	ppiD	-2.1	0.0483
Q9I5I4_PSEAE	Probable enoyl-CoA hydratase/isomerase	DR97_1239	-2.1	0.0127
BAUA_PSEAE	Beta-alanine--pyruvate aminotransferase	aptA	-2.1	0.0897
Q9I078_PSEAE	Putative peroxidase	DR97_5186	-2.1	0.0427
METZ_PSEAE	O-succinylhomoserine sulfhydrylase	metZ	-2.2	0.0308
Q9I1R8_PSEAE	Probable dehydrogenase	yfjR	-2.2	0.0077
RNPH_PSEAE	Ribonuclease PH	rph	-2.2	0.0298
NUOG_PSEAE	NADH-quinone oxidoreductase subunit G	nuoG	-2.2	0.0875
Q9I6Y9_PSEAE	Nonspecific ribonucleoside hydrolase	nuh	-2.2	0.0295
IMM2_PSEAE	Pyocin-S2 immunity protein	imm1	-2.2	0.0715
GPMI_PSEAE	2,3-bisphosphoglycerate-independent phosphoglycerate mutase	gpmI	-2.2	0.0357
ISCS_PSEAE	Cysteine desulfurase IscS	iscS1	-2.2	0.1261
HUTH_PSEAE	Histidine ammonia-lyase	hutH	-2.3	0.0090
ASTE_PSEAE	Succinylglutamate desuccinylase	astE	-2.3	0.0397
G3XD40_PSEAE	Probable acyl-CoA thiolase	thlA	-2.3	0.0370
Q9I1E8_PSEAE	Acyl-CoA dehydrogenase	DR97_6102	-2.3	0.1242
HEM3_PSEAE	Porphobilinogen deaminase	hemC	-2.3	0.0314
ISPF_PSEAE	2-C-methyl-D-erythritol 2,4-cyclodiphosphate synthase	ispF	-2.3	0.0000
HSLO_PSEAE	33 kDa chaperonin (Heat shock protein 33 homolog)	hslO	-2.3	0.0869
Q9I612_PSEAE	Probable acyl-CoA dehydrogenase	DR97_3474	-2.3	0.1165
HEM6_PSEAE	Oxygen-dependent coproporphyrinogen-III oxidase	hemF	-2.4	0.0429
Q9HYK7_PSEAE	Ferredoxin--NADP+ reductase	fpr	-2.4	0.0160
Q9HVS6_PSEAE	DUF541 domain-containing protein	DR97_1674	-2.4	0.0590
Q9I5I3_PSEAE	Probable acyl-CoA dehydrogenase	Acad8	-2.4	0.0522
Q820A5_PSEAE	Probable pyridoxamine 5'-phosphate oxidase	phzD	-2.4	0.1208
Q9HUA7_PSEAE	Probable binding protein component of ABC transporter	fliY_1	-2.5	0.0264
G3XDB0_PSEAE	Catabolite repression control protein	crc	-2.5	0.0998
BRAC_PSEAE	Leucine-, isoleucine-, valine-, threonine-, and alanine-binding protein	braC	-2.5	0.0367
Y2116_PSEAE	Putative hydro-lyase PA2116	DR97_5736	-2.5	0.0298
MTNA_PSEAE	Methylthioribose-1-phosphate isomerase	mtnA	-2.5	0.0604
Q9I298_PSEAE	Putative 3-methylglutaconyl-CoA hydratase	liuC	-2.5	0.0417
FUMC1_PSEAE	Fumarate hydratase class II 1	fumC	-2.5	0.0494
Q9HU92_PSEAE	N-formylglutamate amidohydrolase	hutG3	-2.5	0.0285
FABY_PSEAE	Beta-ketoacyl-[acyl-carrier-protein] synthase FabY	DR97_2542	-2.5	0.0286
Q9HZC5_PSEAE	Aminopeptidase N	pepN	-2.5	0.0369
FABI_PSEAE	Enoyl-[acyl-carrier-protein] reductase [NADH] FabI	fabI	-2.5	0.0169
MTND_PSEAE	Acireductone dioxygenase	mtnD	-2.6	0.0277

Entry name	Protein function	STRING ID	Log_2 ΔLFQ	q value
ARUC_PSEAE	Succinylornithine transaminase/acetylornithine aminotransferase	aruC	-2.6	0.0094
Q9HW65_PSEAE	Protease	yfkM_1	-2.6	0.0998
DNAJ_PSEAE	Chaperone protein DnaJ	dnaJ	-2.6	0.0454
TGT_PSEAE	Queuine tRNA-ribosyltransferase	tgt	-2.7	0.0487
Q9HVZ7_PSEAE	UDP-N-acetylmuramoyl-tripeptide--D-alanyl-D-alanine ligase	murF	-2.7	0.0135
PYRC_PSEAE	Dihydroorotase	DR97_4415	-2.7	0.0280
ASTB_PSEAE	N-succinylarginine dihydrolase	astB	-2.7	0.0377
PUR2_PSEAE	Phosphoribosylamine--glycine ligase	purD	-2.7	0.0409
PORD_PSEAE	Porin D	oprD5	-2.7	0.0301
Q9HUE4_PSEAE	Homocysteine synthase	cysD1	-2.7	0.0143
CYSM_PSEAE	Cysteine synthase B	cysM	-2.7	0.0305
Q9I5I5_PSEAE	Probable enoyl-CoA hydratase/isomerase	DR97_1240	-2.7	0.0302
ALKD_PSEAE	2-dehydro-3-deoxy-phosphogluconate aldolase	eda_4	-2.7	0.0083
SKPL_PSEAE	Skp-like protein	skp	-2.8	0.0666
Q9HX85_PSEAE	ABC transporter substrate-binding protein	metQ1	-2.8	0.0307
G3XCV4_PSEAE	Probable pyridoxamine 5'-phosphate oxidase	phzD	-2.8	0.0874
AROC_PSEAE	Chorismate synthase	aroC	-2.8	0.0305
PQSD_PSEAE	Anthraniloyl-CoA anthraniloyltransferase	pqsD	-2.8	0.0298
KYNB_PSEAE	Kynurenine formamidase	kynB	-2.9	0.1429
Q9HVX5_PSEAE	Alpha/beta hydrolase	DR97_1618	-2.9	0.0365
RS19_PSEAE	30S ribosomal protein S19	rpsS	-2.9	0.0303
Q9HTI6_PSEAE	Choline ABC transporter substrate-binding protein	gbuC_2	-2.9	0.0405
EFG2_PSEAE	Elongation factor G 2	fusB	-3.0	0.0292
SECB_PSEAE	Protein-export protein SecB	secB	-3.0	0.0761
PHZB2_PSEAE	Phenazine biosynthesis protein PhzB2	phzB2_2	-3.0	0.0271
LIUE_PSEAE	3-hydroxy-3-isohexenylglutaryl-CoA/hydroxy-methylglutaryl-CoA lyase	HMGCL	-3.0	0.0072
Q9I6G0_PSEAE	L-threonine dehydratase	ilvA1	-3.1	0.0297
CLPP2_PSEAE	ATP-dependent Clp protease proteolytic subunit 2	clpP2	-3.1	0.0663
Q9I659_PSEAE	Probable ClpA/B protease ATP binding subunit	clpB1	-3.1	0.0413
CATB_PSEAE	Catalase (Paraquat-inducible catalase isozyme B)	katB	-3.1	0.0274
Q9HT28_PSEAE	Class I SAM-dependent methyltransferase	DR97_2925	-3.2	0.0319
Q9HX12_PSEAE	Aldehyde dehydrogenase, PaaZ	phaJ4	-3.2	0.0277
RMLC_PSEAE	dTDP-4-dehydrorhamnose 3,5-epimerase	rfbC	-3.3	0.0302
PUR8_PSEAE	Adenylosuccinate lyase	DR97_5333	-3.3	0.0139
Q9HY02_PSEAE	S-formylglutathione hydrolase	fghA	-3.3	0.0108
Q9HZ04_PSEAE	Probable glycine betaine-binding protein	gbuC_3	-3.4	0.0158
NUOF_PSEAE	NADH-quinone oxidoreductase subunit F	nuoF	-3.4	0.0368
PHZD1_PSEAE	Phenazine biosynthesis protein PhzD1	DR97_3698	-3.4	0.0135
Q9HZA1_PSEAE	Enamine deaminase RidA	DR97_4810	-3.4	0.0271
EDD_PSEAE	Phosphogluconate dehydratase	edd	-3.5	0.0098
PQSB_PSEAE	2-heptyl-4(1H)-quinolone synthase subunit PqsB	pqsB	-3.6	0.0353
AHPF_PSEAE	Alkyl hydroperoxide reductase subunit F	ahpF	-3.6	0.0090
Q9HTG8_PSEAE	Peptidase M19	DR97_2772	-3.6	0.0275
FADH_PSEAE	Glutathione-independent formaldehyde dehydrogenase	fdhA	-3.7	0.0536
GSHR_PSEAE	Glutathione reductase	gor	-3.7	0.0661
G3P_PSEAE	Glyceraldehyde-3-phosphate dehydrogenase	gap1	-3.8	0.0000
Q9I2F8_PSEAE	Binding protein component of ABC ribose transporter	rbsB	-3.8	0.0000
Q9I6H4_PSEAE	FAD-binding oxidoreductase	DR97_3281	-3.8	0.0132
Q9HZI4_PSEAE	MOSC domain-containing protein	DR97_4915	-3.9	0.0135
Q9I081_PSEAE	Cupin	DR97_5191	-3.9	0.0272
Q9HVR9_PSEAE	Probable binding protein component of ABC transporter	dppA3	-3.9	0.0000
PHZD2_PSEAE	Phenazine biosynthesis protein PhzD2	DR97_3698	-4.0	0.0000
Y3922_PSEAE	Uncharacterized protein PA3922	DR97_3944	-4.1	0.0280
Q9I593_PSEAE	Probable alkyl hydroperoxide reductase	DR97_1096	-4.1	0.0108
Q9HVS5_PSEAE	Probable binding protein component of ABC transporter	dppA9	-4.3	0.0120
Q9I1E7_PSEAE	Alkyl hydroperoxide reductase AhpD	DR97_6101	-4.5	0.0310
Q9HVS1_PSEAE	Probable binding protein component of ABC transporter	dppA5	-4.5	0.0278
Y3332_PSEAE	Uncharacterized PhzA/B-like protein PA3332	DR97_4595	-4.6	0.0294
MMSA_PSEAE	Methylmalonate-semialdehyde dehydrogenase [acylating]	mmsA1	-4.7	0.0103
HUTU_PSEAE	Urocanate hydratase	hutU	-4.8	0.0080
Q9HZ48_PSEAE	Probable binding protein component of ABC sugar transporter	DR97_4746	-5.4	0.0083
PHZB1_PSEAE	Phenazine biosynthesis protein PhzB1	phzB1	-5.7	0.0289
PHZM_PSEAE	Phenazine-1-carboxylate N-methyltransferase	phzM	-5.8	0.0000

Table S7: Differences in *S. aureus* protein abundance (Student's *t*-test, FDR 0.15) in co-culture vs. monoculture exoproteome.

Entry name	Protein function	STRING ID	Log_2 ΔLFQ	q value
WP_000752917.1	CsbD family protein	AID39348.1	3.7	0.0634
WP_000401377.1	phosphopantetheine adenylyltransferase	coaD	2.0	0.0480
WP_000447678.1	beta-hydroxyacyl-ACP dehydratase	fabZ	2.0	0.0873
WP_000228670.1	protein-tyrosine-phosphatase	ptpA	1.9	0.0000
WP_000180460.1	DUF1128 domain-containing protein	AID40418.1	1.8	0.0489
WP_000818278.1	succinate dehydrogenase flavoprotein subunit	sdhA	1.5	0.0704
WP_000181127.1	polyisoprenoid-binding protein	AID41402.1	1.4	0.0733

Entry name	Protein function	STRING ID	Log_2 ΔLFQ	q value
WP_000018608.1	cell division protein SepF	sepF	1.4	0.1032
WP_000848351.1	phosphoribosylformylglycinamidine synthase subunit PurS	purS	1.2	0.0680
WP_001231458.1	diacylglycerol kinase	dagK	1.2	0.0886
WP_001123276.1	tautomerase	AID39820.1	1.1	0.0853
WP_000214898.1	DUF2187 domain-containing protein	AID39466.1	1.0	0.1477
WP_000277983.1	formimidoylglutamase	hutG	1.0	0.1327
WP_000457386.1	50S ribosomal protein L21	rplU	1.0	0.0484
WP_000075713.1	heme-dependent peroxidase	AID39084.1	0.9	0.0000
WP_000781946.1	zinc-binding alcohol dehydrogenase	AID40879.1	0.9	0.0843
WP_000811163.1	thioredoxin	AID40215.1	0.8	0.0535
WP_000446724.1	7-cyano-7-deazaguanine synthase QueC	queC	0.8	0.0725
WP_000934799.1	single-stranded DNA-binding protein	ssb	0.8	0.1487
WP_000249652.1	tRNA uridine-5-carboxymethylaminomethyl(34) synthesis enzyme MnmG	gidA	0.8	0.0476
WP_000668339.1	cell-wall-binding lipoprotein	AID39529.1	0.8	0.0367
WP_000640738.1	1-phosphofructokinase	fruB	0.8	0.0511
WP_000148605.1	redox-regulated molecular chaperone Hsp33	hslO	0.7	0.0966
WP_000032653.1	NUDIX domain-containing protein	AID40145.1	0.7	0.0904
WP_001793983.1	aminopeptidase PepS	AID40417.1	0.6	0.0681
WP_000197806.1	phosphopentomutase	deoB	0.6	0.0564
WP_001261460.1	30S ribosomal protein S6	rpsF	0.6	0.1468
WP_001096577.1	50S ribosomal protein L30	rpmD	0.6	0.1022
WP_000985618.1	bacillithiol system redox-active protein YtxJ	AID39249.1	0.5	0.1490
WP_000193707.1	phosphogluconate dehydrogenase (NADP(+)-dependent, decarboxylating)	gnd	0.5	0.0564
WP_000631982.1	cysteine--tRNA ligase	cysS	0.5	0.1474
WP_000160304.1	purine-nucleoside phosphorylase	AID40824.1	0.5	0.0881
WP_000514375.1	nitrate reductase subunit alpha	narG	0.4	0.0532
WP_000863437.1	dihydrolipoyllysine-residue acetyltransferase component of pyruvate dehydrogenase complex	pdhC	0.4	0.0569
WP_000918664.1	DNA-directed RNA polymerase subunit beta	rpoB	0.4	0.0871
WP_000734077.1	alanine--tRNA ligase	alaS	0.4	0.0708
WP_001260089.1	triose-phosphate isomerase	tpiA	0.4	0.1029
WP_000626504.1	elongation factor P	efp	0.3	0.1456
WP_000035325.1	pyruvate dehydrogenase E1 component subunit alpha	pdhA	0.3	0.1208
WP_000081181.1	beta-ketoacyl-[acyl-carrier-protein] synthase II	fabF	-0.2	0.1218
WP_000424963.1	GMP synthase (glutamine-hydrolyzing)	guaA	-0.3	0.0931
WP_000842032.1	lipase	AID41384.1	-0.3	0.0966
WP_000933774.1	ribose-phosphate pyrophosphokinase	prs	-0.3	0.1130
WP_000057594.1	cysteine synthase	cysK	-0.3	0.0700
WP_000068176.1	pyruvate dehydrogenase E1 component subunit beta	pdhB	-0.3	0.0863
WP_000186043.1	tyrosine--tRNA ligase	tyrS	-0.4	0.0715
WP_000473654.1	glucose-specific phosphotransferase enzyme IIA component	crr	-0.4	0.1110
WP_000277602.1	universal stress protein	AID40175.1	-0.4	0.0843
WP_000082722.1	oligoendopeptidase F	AID39438.1	-0.4	0.0603
WP_000048712.1	uracil phosphoribosyltransferase	upp	-0.5	0.0704
WP_001251219.1	glucosamine-6-phosphate isomerase	AID40324.1	-0.5	0.0892
WP_000098285.1	lipoteichoic acid synthase	ltaS	-0.5	0.1277
WP_001185462.1	hydroxymethylpyrimidine/phosphomethylpyrimidine kinase	AID39077.1	-0.5	0.0691
WP_000863474.1	signal recognition particle protein	ffh	-0.5	0.1045
WP_000157650.1	50S ribosomal protein L25	ctc	-0.5	0.0873
WP_000959426.1	alanine dehydrogenase	ald1	-0.5	0.0668
WP_001130051.1	malate:quinone oxidoreductase	AID41318.1	-0.5	0.0541
WP_001074749.1	phosphoglycerate kinase	pgk	-0.5	0.0693
WP_000034728.1	pyridoxal 5'-phosphate synthase lyase subunit PdxS	pdxS	-0.5	0.0714
WP_000358009.1	UDP-N-acetylglucosamine 1-carboxyvinyltransferase	murA1	-0.5	0.1316
WP_000532966.1	YebC/PmpR family DNA-binding transcriptional regulator	AID39177.1	-0.5	0.1221
WP_000908974.1	phenylalanine--tRNA ligase subunit beta	pheT	-0.6	0.0440
WP_000169223.1	6-phospho-beta-galactosidase	lacG	-0.6	0.1409
WP_000185311.1	glycerophosphodiester phosphodiesterase	glpQ	-0.6	0.0613
WP_000955797.1	bifunctional acetaldehyde-CoA/alcohol dehydrogenase	adhE	-0.6	0.0499
WP_000210828.1	serine/threonine dehydratase	tdcB	-0.7	0.0523
WP_000116227.1	class II fumarate hydratase	fumC	-0.7	0.0644
WP_000279414.1	Glyceraldehyde-3-phosphate dehydrogenase 1	gapA1	-0.7	0.0501
WP_000858795.1	asparagine--tRNA ligase	asnS	-0.7	0.0706
WP_000670755.1	FAA hydrolase family protein	AID39404.1	-0.7	0.0469
WP_001230225.1	hydroxymethylbilane synthase	hemC	-0.7	0.1326
WP_000257888.1	DUF1447 family protein	AID39527.1	-0.7	0.0885
WP_001218603.1	aerobic glycerol-3-phosphate dehydrogenase	glpD	-0.7	0.1176
WP_000958522.1	chromosome partitioning protein ParA	AID40854.1	-0.7	0.0703
WP_000813536.1	pyruvate decarboxylase	AID38614.1	-0.7	0.0518
WP_000290401.1	aldehyde dehydrogenase	aldA	-0.8	0.1416
WP_000166055.1	fatty acid-binding protein DegV	AID39887.1	-0.8	0.0694
WP_000138487.1	NAD-specific glutamate dehydrogenase	gluD	-0.8	0.0720
WP_001043863.1	DNA-binding protein HU	AID39935.1	-0.8	0.0752
WP_000202178.1	glycine dehydrogenase	gcvPB	-0.8	0.0884
WP_000219068.1	catabolite control protein A	ccpA	-0.8	0.0852
WP_001118708.1	nitrite reductase large subunit	nasD	-0.8	0.1120
WP_000872486.1	2-C-methyl-D-erythritol 4-phosphate cytidylyltransferase	tarI	-0.8	0.0656
WP_000136257.1	thiol peroxidase Tpx	tpx	-0.9	0.0498

Entry name	Protein function	STRING ID	Log_2 ΔLFQ	q value
WP_000860045.1	3-methyl-2-oxobutanoate hydroxymethyltransferase	panB	-0.9	0.0575
WP_000178881.1	30S ribosomal protein S8	rpsH	-0.9	0.0524
WP_001274017.1	30S ribosomal protein S20	rpsT	-0.9	0.0711
WP_001074353.1	carbamate kinase 1	arcC1	-0.9	0.0684
WP_000503824.1	molybdenum cofactor biosynthesis protein	moaB	-1.0	0.0547
WP_001279341.1	PDZ domain-containing protein	AID40197.1	-1.0	0.0931
WP_000867193.1	dihydroxyacetone kinase subunit L	AID39159.1	-1.1	0.0833
WP_000902175.1	hypothetical protein	AID39870.1	-1.1	0.0547
WP_000163281.1	type 1 glutamine amidotransferase	AID40346.1	-1.1	0.1177
WP_001172340.1	acetyl-CoA synthetase	AID41319.1	-1.1	0.0505
WP_000737654.1	manganese transport protein C (MntC)	AID39138.1	-1.2	0.1210
WP_000660054.1	argininosuccinate synthase	argG	-1.2	0.0588
WP_000167269.1	3-oxoacyl-[acyl-carrier-protein] reductase	fabG	-1.2	0.0603
WP_000437472.1	phosphocarrier protein HPr	AID39520.1	-1.2	0.0484
WP_001124985.1	arginine repressor	AID39985.1	-1.2	0.0590
WP_000792567.1	immunoglobulin-binding protein sbi	sbi	-1.2	0.1414
WP_001283444.1	MarR family transcriptional regulator	rat	-1.3	0.1475
WP_000163995.1	6-phospho-beta-glucosidase	bglA	-1.3	0.0491
WP_000036076.1	HTH-type transcriptional regulator SarR	sarR	-1.3	0.0657
WP_000186306.1	Cof-type HAD-IIB family hydrolase	AID39789.1	-1.3	0.0562
WP_001051856.1	YtxH domain-containing protein	AID40224.1	-1.3	0.0714
WP_000649898.1	glutamate ABC transporter permease	AID40328.1	-1.3	0.0692
WP_001270861.1	glutamate-1-semialdehyde 2,1-aminomutase	hemL1	-1.3	0.0551
WP_001018677.1	transcriptional regulator SarA	sarA	-1.3	0.1165
WP_001030080.1	glycine--tRNA ligase	glyQS	-1.5	0.0693
WP_000690439.1	pyridoxal 5'-phosphate synthase glutaminase subunit PdxT	pdxT	-1.5	0.0573
WP_000942303.1	ABC transporter ATP-binding protein	AID39848.1	-1.5	0.1493
WP_000717560.1	ATP-dependent 6-phosphofructokinase	pfkA	-1.5	0.1047
AIO22395.1	p_multicopper oxidase	#N/A	-1.5	0.0981
WP_000277155.1	hypothetical multicopper oxidase protein	#N/A	-1.5	0.0991
WP_000545373.1	Asp-tRNA(Asn)/Glu-tRNA(Gln) amidotransferase GatCAB subunit B	gatB	-1.5	0.0510
WP_000751265.1	transglycosylase IsaA	isaA	-1.6	0.1188
WP_000019697.1	aminomethyl-transferring glycine dehydrogenase	gcvPA	-1.6	0.0718
WP_000745926.1	clumping factor B (ClfB)	clfB	-1.6	0.0881
WP_001283612.1	redox-sensing transcriptional repressor Rex	rex	-1.7	0.0488
WP_000735554.1	putative exported protein	AID39120.1	-1.7	0.0360
WP_000876758.1	HTH-type transcriptional regulator SarS	sarS	-1.7	0.0524
WP_000057330.1	UMP kinase	pyrH	-1.7	0.0862
WP_000134231.1	GTP cyclohydrolase I FolE2	folE2	-1.8	0.0400
WP_000088195.1	cysteine hydrolase	entB2	-1.9	0.0430
WP_000097322.1	ribosome-binding factor A	rbfA	-2.0	0.0892
WP_001174260.1	YpdA family putative bacillithiol disulfide reductase	AID39942.1	-2.2	0.0270
WP_000027924.1	Asp-tRNA(Asn)/Glu-tRNA(Gln) amidotransferase GatCAB subunit A	gatA	-2.4	0.0522
WP_000617735.1	ribosomal subunit interface protein	hpf	-3.4	0.0510
WP_000976253.1	dihydroxyacetone kinase subunit DhaK	AID39158.1	-3.5	0.0432

Table S8: Differentially expressed *P. aeruginosa* proteins (Student's *t*-test, FDR 0.15) in co-culture vs. monoculture surfaceome.

Entry name	Protein function	STRING ID	Log_2 ΔLFQ	q value
ODO2_PSEAE	Dihydrolipoyllysine-residue succinyltransferase component of 2-oxoglutarate dehydrogenase complex	sucB	6.7	0.0000
ETFB_PSEAE	Electron transfer flavoprotein subunit beta	etfB	5.5	0.0048
RL2_PSEAE	50S ribosomal protein L2	rplB	5.4	0.0057
Q9HTQ4_PSEAE	Cytochrome c5	cycB	5.3	0.0000
Q9HX76_PSEAE	Probable DNA binding protein	hupA	5.2	0.0045
EFP_PSEAE	Elongation factor P	efp	5.0	0.0037
RL9_PSEAE	50S ribosomal protein L9	rplI	4.9	0.0000
AZUR_PSEAE	Azurin	azu	4.9	0.0049
METK_PSEAE	S-adenosylmethionine synthase	metK	4.7	0.0040
ILVC_PSEAE	Ketol-acid reductoisomerase (NADP(+))	ilvC	4.5	0.0059
Q9HTD1_PSEAE	Probable transcarboxylase subunit	oadA	4.4	0.0036
Q9HW68_PSEAE	Fumarate hydratase class I	fumA	4.3	0.0000
Q9HV96_PSEAE	Polynucleotide kinase	DR97_2043	4.3	0.0000
Q9HVU0_PSEAE	Rod shape-determining protein MreB	mreB	4.3	0.0000
Q9HVA3_PSEAE	Phosphatidylserine synthase	pssA	4.2	0.0000
CATA_PSEAE	Catalase	katA	4.2	0.0150
P5217_PSEAE	Probable binding protein component of ABC iron transporter PA5217	DR97_2586	4.1	0.0052
Q9I5I4_PSEAE	Probable enoyl-CoA hydratase/isomerase	DR97_1239	4.0	0.0136
ALGP_PSEAE	Transcriptional regulatory protein AlgP	algP	4.0	0.0055
GLN1B_PSEAE	Glutamine synthetase	glnA	4.0	0.0164
RL16_PSEAE	50S ribosomal protein L16	rplP	3.9	0.0000
NUSG_PSEAE	Transcription termination/antitermination protein NusG	nusG	3.9	0.0039

Entry name	Protein function	STRING ID	Log_2 ΔLFQ	q value
RF3_PSEAE	Peptide chain release factor 3	prfC	3.8	0.0073
Q9I3D3_PSEAE	2-oxoglutarate dehydrogenase (E1 subunit)	sucA	3.8	0.0000
SPUE_PSEAE	Spermidine-binding periplasmic protein SpuE	potF3	3.8	0.0050
PQSD_PSEAE	Anthraniloyl-CoA anthraniloyltransferase	pqsD	3.8	0.0133
SYC_PSEAE	Cysteine--tRNA ligase	cysS	3.8	0.0000
IPYR_PSEAE	Inorganic pyrophosphatase	ppa	3.8	0.0000
RL11_PSEAE	50S ribosomal protein L11	rplK	3.7	0.0042
ENO_PSEAE	Enolase	eno	3.7	0.0162
Q9HUT3_PSEAE	Probable bacterioferritin	DR97_2231	3.6	0.0057
Q9HVF9_PSEAE	M48 family peptidase	DR97_1939	3.6	0.0045
Q9I4I1_PSEAE	Ribonucleoside-diphosphate reductase	nrdA	3.5	0.0041
NUOB_PSEAE	NADH-quinone oxidoreductase subunit B	nuoB	3.4	0.0000
Q9I5E5_PSEAE	2-methylaconitate cis-trans isomerase PrpF	prpF	3.4	0.0281
Q9HVW4_PSEAE	ABC transporter periplasmic binding protein MlaC	ttg2D	3.4	0.0000
Q9HW72_PSEAE	Pyruvate kinase	pykA	3.4	0.0728
PYRX_PSEAE	Dihydroorotase-like protein	pyrC'	3.4	0.0000
Q9HW45_PSEAE	Xenobiotic reductase	nemA3	3.3	0.0077
SYI_PSEAE	Isoleucine--tRNA ligase	ileS	3.3	0.0163
FABY_PSEAE	Beta-ketoacyl-[acyl-carrier-protein] synthase FabY	DR97_2542	3.3	0.0163
CBPD_PSEAE	Chitin-binding protein CbpD (Protease LasD)	cbpD	3.3	0.0193
GLMU_PSEAE	Bifunctional protein GlmU	glmU	3.3	0.0165
Q9I5F6_PSEAE	Bifunctional protein PutA	putA	3.3	0.0073
PYRB_PSEAE	Aspartate carbamoyltransferase	pyrB	3.2	0.0043
CLPV1_PSEAE	Protein ClpV1	clpV1-1	3.2	0.0137
MQO2_PSEAE	Probable malate:quinone oxidoreductase 2	mqo2	3.2	0.0056
GATB_PSEAE	Aspartyl/glutamyl-tRNA	gatB	3.1	0.0075
PILH_PSEAE	Protein PilH	pilH	3.1	0.0109
GALU_PSEAE	UTP--glucose-1-phosphate uridylyltransferase	galU	3.1	0.0055
PPK1_PSEAE	Polyphosphate kinase	ppk1	3.1	0.0135
Q9HV83_PSEAE	Probable aminotransferase	alaC	3.0	0.0115
Q9HVJ1_PSEAE	Probable ATP-binding component of ABC transporter	DR97_1900	3.0	0.0053
IF3_PSEAE	Translation initiation factor IF-3	infC	3.0	0.1142
BAMD_PSEAE	Outer membrane protein assembly factor BamD	yfiO	3.0	0.0061
PHZM_PSEAE	Phenazine-1-carboxylate N-methyltransferase	phzM	3.0	0.0161
ISPG_PSEAE	4-hydroxy-3-methylbut-2-en-1-yl diphosphate synthase	ispG	3.0	0.0158
Q9I5A3_PSEAE	Peptidyl-prolyl cis-trans isomerase	slyD	3.0	0.0033
Q9HTP2_PSEAE	Probable aldehyde dehydrogenase	puuC5	2.9	0.0045
ATPB_PSEAE	ATP synthase subunit beta	atpD	2.9	0.0000
SECA_PSEAE	Protein translocase subunit SecA	secA	2.9	0.0048
Q9HW32_PSEAE	Insulin-cleaving metalloproteinase outer membrane protein	icmP	2.9	0.0113
Q9I2W9_PSEAE	Phosphoenolpyruvate synthase	ppsA	2.9	0.0032
Q9HXP8_PSEAE	Signal recognition particle protein	ffh	2.9	0.0000
Q9HVA8_PSEAE	Ferric iron-binding periplasmic protein HitA	fbpA	2.9	0.0047
PHZD1_PSEAE	Phenazine biosynthesis protein PhzD1	DR97_3698	2.9	0.0131
RS19_PSEAE	30S ribosomal protein S19	rpsS	2.9	0.0035
G6PI_PSEAE	Glucose-6-phosphate isomerase	pgi	2.9	0.0041
Q9I765_PSEAE	Oligopeptidase A	prlC	2.8	0.0046
SYK_PSEAE	Lysine--tRNA ligase	lysS	2.8	0.0283
Q9I4I2_PSEAE	Ribonucleoside-diphosphate reductase subunit beta	nrdB	2.8	0.0708
PHZD2_PSEAE	Phenazine biosynthesis protein PhzD2	DR97_3698	2.8	0.0163
ALR2_PSEAE	Alanine racemase, catabolic	dadX	2.8	0.0030
SAHH_PSEAE	Adenosylhomocysteinase	ahcY	2.8	0.0130
SYR_PSEAE	Arginine--tRNA ligase	argS	2.7	0.0061
Q9HW49_PSEAE	Universal stress protein	DR97_1527	2.7	0.0167
PVDA_PSEAE	L-ornithine N(5)-monooxygenase	pvdA	2.7	0.0067
CATB_PSEAE	Catalase B	katB	2.6	0.0035
Q9HTD9_PSEAE	Alcohol dehydrogenase	adhA	2.6	0.0159
Q9HVX4_PSEAE	Cytochrome D ubiquinol oxidase subunit III	DR97_1619	2.6	0.0034
STHA_PSEAE	Soluble pyridine nucleotide transhydrogenase	sthA	2.6	0.0111
Q9I4S1_PSEAE	17 kDa surface antigen	slyB	2.6	0.0039
LEU1_PSEAE	2-isopropylmalate synthase	leuA	2.5	0.0063
Q9I5E2_PSEAE	2-methylisocitrate lyase	prpB	2.5	0.0036
DAPA_PSEAE	4-hydroxy-tetrahydrodipicolinate synthase	dapA_1	2.5	0.0129
PARE_PSEAE	DNA topoisomerase 4 subunit B	parE	2.5	0.0058
SYFB_PSEAE	Phenylalanine--tRNA ligase beta subunit	pheT	2.5	0.0145
BFR_PSEAE	Bacterioferritin	bfr1	2.4	0.0161
TOP1_PSEAE	DNA topoisomerase 1	DR97_4926	2.4	0.0054
BAUA_PSEAE	Beta-alanine--pyruvate aminotransferase	aptA	2.4	0.0170
PROA_PSEAE	Gamma-glutamyl phosphate reductase	proA	2.4	0.0136
Q9HUA1_PSEAE	Probable binding protein component of ABC transporter	gltI1	2.4	0.0156
SURA_PSEAE	Chaperone SurA	surA	2.4	0.0061
PILJ_PSEAE	Protein PilJ	pilJ	2.4	0.0163
ATPG_PSEAE	ATP synthase gamma chain	atpG	2.4	0.0162
PK21A_PSEAE	Polyphosphate:ADP/GDP phosphotransferase	DR97_3098	2.3	0.0132
Q9HUE1_PSEAE	ATPase nvolved in chromosome partitioning	DR97_2383	2.3	0.0038
G3XCT6_PSEAE	Probable two-component sensor	DR97_565	2.3	0.0196
Q9HT76_PSEAE	Vitamin B12-dependent ribonucleotide reductase	nrdZ	2.2	0.0279
Q9I659_PSEAE	Probable ClpA/B protease ATP binding subunit	clpB1	2.2	0.0165
RDGC_PSEAE	Recombination-associated protein RdgC	rdgC	2.2	0.0195
RS9_PSEAE	30S ribosomal protein S9	rpsI	2.2	0.0882
Q9HZP1_PSEAE	Probable transcriptional regulator	icaR	2.2	0.0060

Entry name	Protein function	STRING ID	Log_2 ΔLFQ	q value
Q9I2C4_PSEAE	NAD+ dependent aldehyde dehydrogenase ExaC	aldA3	2.2	0.0243
Q9I2T4_PSEAE	Probable binding protein component of ABC transporter	DR97_69	2.2	0.0130
Q9HU13_PSEAE	ADP compounds hydrolase NudE	nudE	2.2	0.0037
ASPA_PSEAE	Aspartate ammonia-lyase	aspA	2.2	0.0112
Q9HX05_PSEAE	Probable aldehyde dehydrogenase	aldA1	2.2	0.0161
Q9I5E4_PSEAE	Probable aconitate hydratase	acnD	2.2	0.0210
Q9I500_PSEAE	HIT domain-containing protein	DR97_978	2.1	0.0766
Q9HZ32_PSEAE	NAD(P)H nitroreductase YdjA	ydjA_2	2.1	0.0039
CYSM_PSEAE	Cysteine synthase B	cysM	2.1	0.0244
PROB_PSEAE	Glutamate 5-kinase	proB	2.1	0.0166
Q9HV73_PSEAE	Two-component response regulator CbrB	zraR1	2.1	0.0967
ARLY_PSEAE	Argininosuccinate lyase	argH	2.1	0.0285
PUR7_PSEAE	Phosphoribosylaminoimidazole-succinocarboxamide synthase	purC	2.1	0.0386
GSHB_PSEAE	Glutathione synthetase	gshB	2.1	0.0043
DAPB_PSEAE	4-hydroxy-tetrahydrodipicolinate reductase	dapB	2.0	0.1209
AROB_PSEAE	3-dehydroquinate synthase	aroB	2.0	0.0181
Q9I2T8_PSEAE	Peptidylprolyl isomerase	ppiD	2.0	0.0000
Q9HYR6_PSEAE	Condensation domain protein	DR97_4598	2.0	0.0054
DSBA_PSEAE	Thiol:disulfide interchange protein DsbA	dsbA	2.0	0.0158
PDXJ_PSEAE	Pyridoxine 5'-phosphate synthase	pdxJ	2.0	0.0040
Q9HVV5_PSEAE	Ribosomal subunit interface protein	raiA	1.9	0.0139
A2MGH_PSEAE	Alpha-2-macroglobulin homolog	DR97_1668	1.9	0.0159
Q9I649_PSEAE	Protein phosphatase 2C domain-containing protein	DR97_3437	1.9	0.0155
ZIPA_PSEAE	Cell division protein ZipA	zipA	1.9	0.0319
Q9I3X2_PSEAE	DNA helicase	#N/A	1.9	0.0074
GYRB_PSEAE	DNA gyrase subunit B	gyrB	1.9	0.0042
Q9HVS1_PSEAE	Probable binding protein component of ABC transporter	dppA5	1.9	0.0057
Q9I171_PSEAE	Metal ABC transporter substrate-binding protein	DR97_6023	1.9	0.0226
Q9I0D5_PSEAE	AAA ATPase	DR97_5252	1.8	0.0211
ILVE_PSEAE	Branched-chain-amino-acid aminotransferase	ilvE	1.8	0.0110
ARCC_PSEAE	Carbamate kinase	arcC	1.8	0.1213
GSA_PSEAE	Glutamate-1-semialdehyde 2,1-aminomutase	hemL	1.8	0.0165
Q9HVK8_PSEAE	Endopeptidase La	DR97_1880	1.8	0.0168
OBG_PSEAE	GTPase Obg	cgtA	1.8	0.0844
Q9I338_PSEAE	LTA synthase family protein	DR97_198	1.8	0.0152
PUR5_PSEAE	Phosphoribosylformylglycinamidine cyclo-ligase	purM	1.7	0.0164
PYRG_PSEAE	CTP synthase	pyrG	1.7	0.0157
Y3998_PSEAE	UPF0250 protein PA3998	DR97_3869	1.7	0.0076
COBW_PSEAE	Protein CobW	cobW	1.7	0.0355
Q9HXK0_PSEAE	(R)-stereoselective amidase	yafV	1.7	0.0157
Q9I2N2_PSEAE	Molybdate-binding periplasmic protein ModA	modA	1.7	0.0135
Q9HTI6_PSEAE	Choline ABC transporter substrate-binding protein	gbuC_2	1.6	0.0044
LOLA_PSEAE	Outer-membrane lipoprotein carrier protein	lolA	1.6	0.0149
BAUC_PSEAE	Putative 3-oxopropanoate dehydrogenase	iolA2	1.6	0.1390
E1JGJ8_PSEAE	Peptide chain release factor 2	prfB	1.6	0.0162
ARGB_PSEAE	Acetylglutamate kinase	argB	1.6	0.0384
Q9I508_PSEAE	Arsenate reductase	DR97_988	1.6	0.0140
MURA_PSEAE	UDP-N-acetylglucosamine 1-carboxyvinyltransferase	murA	1.6	0.0161
MTNA_PSEAE	Methylthioribose-1-phosphate isomerase	mtnA	1.6	0.0058
PURA_PSEAE	Adenylosuccinate synthetase	purA	1.6	0.0831
Q7DC81_PSEAE	Phenazine biosynthesis protein PhzE	DR97_3697	1.6	0.0424
CLPP2_PSEAE	ATP-dependent Clp protease proteolytic subunit 2	clpP2	1.5	0.1218
G3XD17_PSEAE	Thiol:disulfide interchange protein	dsbC	1.5	0.1166
HISZ_PSEAE	ATP phosphoribosyltransferase regulatory subunit	hisZ	1.5	0.0078
RPSH_PSEAE	RNA polymerase sigma-H factor	algU	1.5	0.0427
TOLR_PSEAE	Tol-Pal system protein TolR	tolR	1.5	0.0353
Q9HWW7_PSEAE	Probable thioredoxin	trx-1	1.4	0.0172
DPO1_PSEAE	DNA polymerase I	polA	1.4	0.1137
Q9HZI4_PSEAE	MOSC domain-containing protein	DR97_4915	1.4	0.0147
PCTC_PSEAE	Methyl-accepting chemotaxis protein PctC	pctC	1.4	0.0778
RIBB_PSEAE	3,4-dihydroxy-2-butanone 4-phosphate synthase	ribB	1.4	0.1137
Q9HXJ3_PSEAE	Cro/Cl family transcriptional regulator	DR97_4065	1.4	0.1213
GCSH2_PSEAE	Glycine cleavage system H protein 2	DR97_2583	1.4	0.0614
TAL_PSEAE	Transaldolase	tal	1.4	0.0380
Q9I6G0_PSEAE	L-threonine dehydratase	ilvA1	1.3	0.0184
Q9I2S7_PSEAE	Lysine-specific pyridoxal 5'-phosphate-dependent carboxylase, LdcA	adiA	1.3	0.0388
HEM6_PSEAE	Oxygen-dependent coproporphyrinogen-III oxidase	hemF	1.3	0.0347
COAD_PSEAE	Phosphopantetheine adenylyltransferase	coaD	1.3	0.0163
Q9I748_PSEAE	EvpB family type VI secretion protein TssC	DR97_3041	1.3	0.0357
HIS7_PSEAE	Imidazoleglycerol-phosphate dehydratase	hisB	1.3	0.0253
G3XD54_PSEAE	DNA polymerase III subunit epsilon	dnaQ	1.2	0.0727
PCTA_PSEAE	Methyl-accepting chemotaxis protein PctA	pctA	1.2	0.1263
Q9I0T0_PSEAE	Probable short-chain dehydrogenase	linC_1	1.2	0.0201
Q9I6J5_PSEAE	Probable glutamine synthetase	puuA	1.2	0.1391
Q9HVL5_PSEAE	Octaprenyl-diphosphate synthase	ispB	1.2	0.0295
SYT_PSEAE	Threonine--tRNA ligase	thrS	1.1	0.0213
Q9I5V9_PSEAE	Glutamyl-tRNA amidotransferase	DR97_3547	1.1	0.0657
Q9HW65_PSEAE	Protease	yfkM_1	1.1	0.1338
Q9I348_PSEAE	Cysteine hydrolase	DR97_211	1.1	0.0477
Q9I157_PSEAE	PvdL	pvdL	1.0	0.0564
Q9I704_PSEAE	Alkylphosphonate utilization protein	DR97_3085	1.0	0.0182

Entry name	Protein function	STRING ID	Log_2 ΔLFQ	q value
GCH1L_PSEAE	GTP cyclohydrolase 1 type 2 homolog	DR97_1622	1.0	0.0283
Q9HV81_PSEAE	Neutral zinc metallopeptidase	ypfJ	1.0	0.0457
HIS3_PSEAE	Phosphoribosyl-AMP cyclohydrolase	hisI	1.0	0.0445
PCTB_PSEAE	Methyl-accepting chemotaxis protein PctB	pctB	1.0	0.1185
Q9HV64_PSEAE	DUF748 domain-containing protein	DR97_2078	1.0	0.0474
Q9HW07_PSEAE	ATP:cob	DR97_1577	0.9	0.0314
THIG_PSEAE	Thiazole synthase	thiG	0.9	0.1368
ILVD_PSEAE	Dihydroxy-acid dehydratase	ilvD	0.9	0.1385
GPDA_PSEAE	Glycerol-3-phosphate dehydrogenase [NAD(P)+]	gpsA	0.9	0.1223
Q9HZA6_PSEAE	Motility protein FimV	DR97_4818	0.9	0.0169
Q9HUA3_PSEAE	Proline iminopeptidase	pip	0.9	0.0430
TRMB_PSEAE	tRNA (guanine-N(7)-)-methyltransferase	trmB	0.8	0.0059
RSMH_PSEAE	Ribosomal RNA small subunit methyltransferase H	rsmH	0.8	0.0698
Q9HZ19_PSEAE	CsaA protein	csaA	0.8	0.1440
Q9I067_PSEAE	Putative oxidoreductase	puuB1	0.8	0.1138
Q9HY13_PSEAE	D-xylulose 5-phosphate	DR97_4326	0.6	0.0148
SPUD_PSEAE	Putrescine-binding periplasmic protein SpuD	potF1	0.6	0.0338
Q9HUD3_PSEAE	Malic enzyme	maeB	0.5	0.0617
Q9HV74_PSEAE	Two-component sensor CbrA	cbrA	0.5	0.0716
SYGB_PSEAE	Glycine--tRNA ligase beta subunit	glyS	0.4	0.1218
HIS61_PSEAE	Imidazole glycerol phosphate synthase subunit hisF1	hisF	-0.4	0.1362
Q9HWI2_PSEAE	Probable acyl-CoA dehydrogenase	DR97_3476	-0.4	0.0382
Q9HX44_PSEAE	Probable acyl-CoA dehydrogenase	aidB	-0.6	0.1433
Q9HWZ2_PSEAE	FecR protein	DR97_3832	-0.6	0.0848
PILS_PSEAE	Sensor protein PilS	pilS	-0.7	0.1365
Q9I4V1_PSEAE	Probable short-chain dehydrogenase	DR97_921	-0.7	0.0727
MURE_PSEAE	UDP-N-acetylmuramoyl-L-alanyl-D-glutamate--2,6-diaminopimelate ligase	murE	-0.8	0.0190
PUR4_PSEAE	Phosphoribosylformylglycinamidine synthase	purL	-0.8	0.0928
Q9HZ86_PSEAE	Nucleotide sugar epimerase/dehydratase WbpM	wbpM	-0.8	0.0944
Q9HUM2_PSEAE	Protein HflK	hflK	-0.8	0.1264
SYM_PSEAE	Methionine--tRNA ligase	metG	-0.8	0.0336
Q9HZM8_PSEAE	Ribonuclease E	rne	-0.9	0.0180
Q9HYR8_PSEAE	Probable non-ribosomal peptide synthetase	DR97_4600	-0.9	0.0610
SYQ_PSEAE	Glutamine--tRNA ligase	glnS	-0.9	0.0579
Q9HUG4_PSEAE	Glycosyltransferase	DR97_2356	-1.0	0.1034
Q9HVV6_PSEAE	Probable ATP-binding component of ABC transporter	lptB	-1.0	0.1185
Q9I6C1_PSEAE	Signal recognition particle receptor FtsY	ftsY	-1.0	0.0167
Q9HXY1_PSEAE	Methionine aminopeptidase	map1	-1.0	0.0596
Q9I0Y8_PSEAE	Efflux pump membrane transporter	bepE	-1.0	0.0084
G3XD52_PSEAE	(R)-specific enoyl-CoA hydratase	phaJ	-1.1	0.0179
MASZ_PSEAE	Malate synthase G	glcB	-1.1	0.1213
Q9HXM5_PSEAE	Inosine-5'-monophosphate dehydrogenase	guaB	-1.1	0.1190
Q9I520_PSEAE	Chain-length determining protein	DR97_1001	-1.1	0.0349
MUTL_PSEAE	DNA mismatch repair protein MutL	mutL	-1.1	0.0197
NUOG_PSEAE	NADH-quinone oxidoreductase subunit G	nuoG	-1.2	0.0279
Q9I031_PSEAE	Probable ATP-binding component of ABC transporter	drrA	-1.3	0.0706
Q9I1U6_PSEAE	Aldehyde dehydrogenase	DR97_6257	-1.3	0.0568
Q9HXI0_PSEAE	Preprotein translocase subunit YajC	yajC	-1.3	0.0459
Q9HYB3_PSEAE	Leucyl-tRNA synthetase	DR97_4428	-1.3	0.0160
Q9I755_PSEAE	IcmF1	DR97_3034	-1.4	0.0248
Q9I0A7_PSEAE	Probable restriction-modification system protein	#N/A	-1.4	0.0000
Q9HZ04_PSEAE	Probable glycine betaine-binding protein	gbuC_3	-1.4	0.0191
AMGK_PSEAE	N-acetylmuramate/N-acetylglucosamine kinase	DR97_3565	-1.4	0.0037
Q9HU31_PSEAE	Amino acid (Lysine/arginine/ornithine/histidine/octopine) ABC transporter periplasmic binding protein	artJ	-1.4	0.0137
PUR1_PSEAE	Amidophosphoribosyltransferase	purF	-1.5	0.0345
Q9HYY3_PSEAE	Periplasmic tail-specific protease	tsp	-1.5	0.0483
Q9HU22_PSEAE	Glucose-1-phosphate thymidylyltransferase	rfbA	-1.5	0.0215
RLUB_PSEAE	Ribosomal large subunit pseudouridine synthase B	rluB	-1.7	0.0160
Q9HTL9_PSEAE	Enamine/imine deaminase	yabJ_5	-1.8	0.0134
Q9HUG0_PSEAE	Probable carbamoyl transferase	novN_1	-1.9	0.0724
Q9I0Z1_PSEAE	Oxidoreductase MexS	DR97_5681	-2.2	0.0108
ACSA2_PSEAE	Acetyl-coenzyme A synthetase 2	acsB	-2.2	0.0351
Q9HTT1_PSEAE	Uncharacterized protein	DR97_2640	-2.3	0.0317
Q9HTY1_PSEAE	Inorganic triphosphatase	DR97_2578	-2.3	0.0316
Y587_PSEAE	UPF0229 protein PA0587	DR97_3556	-2.3	0.0035
Q9I179_PSEAE	Probable non-ribosomal peptide synthetase	pvdI	-2.4	0.0000
COQ7_PSEAE	2-nonaprenyl-3-methyl-6-methoxy-1,4-benzoquinol hydroxylase	coq7	-2.4	0.0335
DCTP_PSEAE	C4-dicarboxylate-binding periplasmic protein DctP	dctP1	-2.5	0.0848
Q9I5F9_PSEAE	Lon protease	lon2	-2.6	0.0502
Q9I246_PSEAE	Probable carbamoyl transferase	DR97_5777	-2.6	0.0178
ALGR_PSEAE	Positive alginate biosynthesis regulatory protein	algR	-2.7	0.0829
Q9I2F8_PSEAE	Binding protein component of ABC ribose transporter	rbsB	-3.1	0.0388
Q9HU89_PSEAE	Probable ATP-binding component of ABC transporter	opuAA	-3.3	0.0036
Q9HTT8_PSEAE	Heme biosynthesis protein HemY	hemY	-3.4	0.0192
Q9HTF4_PSEAE	GbcA	gbcA	-3.4	0.0031
SYFA_PSEAE	Phenylalanine--tRNA ligase alpha subunit	pheS	-3.8	0.0106
HUTU_PSEAE	Urocanate hydratase	hutU	-4.7	0.0000
Q9I612_PSEAE	Probable acyl-CoA dehydrogenase	DR97_3474	-5.1	0.0000
Q9HUY5_PSEAE	Mg(2+) transport ATPase, P-type 2	mgtA	-5.2	0.0116

Entry name	Protein function	STRING ID	Log_2 ΔLFQ	q value
Q9I5U9_PSEAE	PrkA AAA domain protein	yeaG	-5.7	0.0000
Q9I2R2_PSEAE	Probable oxidoreductase	yhfP	-5.7	0.0000

Table S9: Differentially express *S. aureus* proteins (Student's *t*-test, FDR 0.15) in co-culture vs. monoculture surfaceome.

Entry name	Protein function	STRING ID	Log_2 ΔLFQ	q value
WP_000792567.1	immunoglobulin-binding protein sbi	sbi	1.3	0.0099
WP_000133953.1	30S ribosomal protein S1	rpsA	0.6	0.0199
WP_000863437.1	dihydrolipoyllysine-residue acetyltransferase component of pyruvate dehydrogenase complex	pdhC	0.3	0.0564
WP_000391030.1	cell division protein FtsA	ftsA	0.2	0.0481
WP_000808968.1	50S ribosomal protein L31 type B	rpmE2	0.2	0.0793
WP_000035325.1	pyruvate dehydrogenase E1 component subunit alpha	pdhA	-0.2	0.0401
WP_001083335.1	2-deoxyribose-5-phosphate aldolase	deoC2	-0.3	0.0525
WP_000723304.1	UDP-glucose 4-epimerase	AID39048.1	-0.4	0.0071
WP_001130051.1	malate:quinone oxidoreductase	AID41318.1	-0.4	0.0027
WP_000863556.1	superoxide dismutase [Mn/Fe] 1 SodA	sodA	-0.5	0.0026
WP_001074619.1	50S ribosomal protein L1	rplA	-0.6	0.0025
WP_000460244.1	peptide chain release factor 1	prfA	-0.6	0.0025
WP_000002746.1	3-oxoacyl-ACP reductase	butA	-0.7	0.0026
WP_001206107.1	aldehyde dehydrogenase family protein	AID40808.1	-0.7	0.0009
WP_000888997.1	cell division protein FtsZ	ftsZ	-0.8	0.0094
WP_000457386.1	50S ribosomal protein L21	rplU	-0.8	0.0000
WP_000116227.1	class II fumarate hydratase	fumC	-0.8	0.1111
WP_001137495.1	30S ribosomal protein S7	rpsG	-0.9	0.0046
WP_000911041.1	rod shape-determining protein MreC	AID40117.1	-0.9	0.0013
WP_000057594.1	cysteine synthase	cysK	-0.9	0.0019
WP_000737711.1	hypothetical membrane lipoprotein	AID41063.1	-1.0	0.0419
WP_000091975.1	50S ribosomal protein L6	rplF	-1.0	0.0000
WP_001033875.1	5'-nucleotidase, lipoprotein e(P4) family	AID38732.1	-1.0	0.0299
WP_000161314.1	glucokinase	glcK	-1.0	0.0016
WP_000975347.1	glycosyltransferase family 2 protein	AID38679.1	-1.0	0.0112
WP_000044796.1	aspartate--tRNA ligase	aspS	-1.0	0.0009
WP_000572877.1	adenylosuccinate lyase	purB	-1.0	0.0093
WP_000095328.1	adenylosuccinate synthetase	purA	-1.0	0.0038
WP_000836472.1	rhodanese-like domain-containing protein	AID40229.1	-1.0	0.0026
WP_001115431.1	dihydrolipoyllysine-residue succinyltransferase component of 2-oxoglutarate dehydrogenase complex	odhB	-1.0	0.0071
WP_000933956.1	DNA-directed RNA polymerase subunit omega	rpoZ	-1.0	0.0059
WP_000255583.1	DNA gyrase subunit B	gyrB	-1.1	0.0016
WP_000811375.1	ribonuclease J 1	rnj1	-1.2	0.0000
WP_000129413.1	arginine deiminase	arcA	-1.2	0.0000
WP_000670755.1	FAA hydrolase family protein	AID39404.1	-1.2	0.0000
WP_000492114.1	UPF0365 family protein	AID40039.1	-1.2	0.0005
WP_001049957.1	peptide chain release factor 3	prfC	-1.2	0.0439
WP_000769416.1	D-alanyl-lipoteichoic acid biosynthesis protein DltD	dltD	-1.2	0.0026
WP_000722165.1	bifunctional phosphopantothenoylcysteine decarboxylase/phosphopantothenate--cysteine ligase CoaBC	AID39651.1	-1.2	0.0032
WP_000105070.1	glucose-6-phosphate dehydrogenase	zwf	-1.2	0.0000
AIO22395.1	p_multicopper oxidase	#N/A	-1.2	0.0000
WP_001006454.1	cysteine desulfurase	AID39352.1	-1.2	0.0016
WP_001021468.1	adenylate kinase	adk	-1.3	0.0122
WP_000064078.1	DNA-binding response regulator	srrA	-1.3	0.0208
WP_000690439.1	pyridoxal 5'-phosphate synthase glutaminase subunit PdxT	pdxT	-1.3	0.0000
WP_000240920.1	hypothetical membrane protein	tarL	-1.3	0.0019
WP_001010762.1	cytochrome ubiquinol oxidase subunit I	qoxB	-1.3	0.0000
WP_000673317.1	glycine glycyltransferase FemA	femA	-1.3	0.0017
WP_000514375.1	nitrate reductase subunit alpha	narG	-1.3	0.0019
WP_001289711.1	uncharacterized N-acetyltransferase protein	AID39615.1	-1.3	0.0000
WP_001074405.1	Fe-S cluster assembly protein SufB	AID39354.1	-1.3	0.0063
WP_000808894.1	aldo/keto reductase	AID39107.1	-1.4	0.0000
WP_001180212.1	NADP-dependent oxidoreductase	AID40881.1	-1.4	0.0000
WP_000956150.1	SMC family protein	AID38875.1	-1.4	0.0205
WP_000136257.1	thiol peroxidase Tpx	tpx	-1.4	0.0016
WP_001147955.1	thioredoxin	AID39315.1	-1.4	0.0013
WP_000684568.1	octopine dehydrogenase	AID40990.1	-1.4	0.0006
WP_000217452.1	SDR family oxidoreductase	AID41172.1	-1.4	0.0000
WP_000757543.1	MarR family transcriptional regulator	rot	-1.4	0.0035
WP_000648118.1	YtxH domain-containing protein	AID40309.1	-1.4	0.0019
WP_000066379.1	3-deoxy-7-phosphoheptulonate synthase	AID40207.1	-1.4	0.0000
WP_000711897.1	signal peptidase IB (spsB)	spsB	-1.4	0.0016
WP_001117071.1	cell division protein DivIVA	AID39631.1	-1.4	0.0000
WP_001794472.1	quinone oxidoreductase	AID41065.1	-1.4	0.0000
WP_001227718.1	PTS glucose EIICBA component	ptsG	-1.4	0.0000
WP_001185462.1	hydroxymethylpyrimidine/phosphomethylpyrimidine kinase	AID39077.1	-1.4	0.0081
WP_000390829.1	sigma-B regulation protein serine phosphatase RsbU	AID40753.1	-1.5	0.0000

Entry name	Protein function	STRING ID	Log_2 ΔLFQ	q value
WP_000150173.1	UDP-N-acetylmuramate--L-alanine ligase	murC	-1.5	0.0016
WP_000897132.1	ATP-dependent Clp protease ATP-binding subunit ClpC	clpC	-1.5	0.0000
WP_000701483.1	DNA-directed RNA polymerase subunit delta	rpoE	-1.5	0.0000
WP_001048374.1	DUF4479 and tRNA_bind_bactPheRS domain-containing protein	AID40213.1	-1.5	0.0000
WP_000610203.1	ribitol-5-phosphate dehydrogenase	tarJ	-1.5	0.0009
WP_000040067.1	acetate kinase	ackA	-1.5	0.0000
WP_000259736.1	molybdopterin molybdenumtransferase	moeA	-1.5	0.0005
WP_000240651.1	molecular chaperone GroEL	groL	-1.5	0.0000
WP_001118708.1	nitrite reductase large subunit	nasD	-1.6	0.0026
WP_000110252.1	succinyl-CoA ligase subunit alpha	sucD	-1.6	0.0000
WP_000754458.1	hypothetical membrane Fe-trasport lipoprotein	AID39244.1	-1.6	0.0006
WP_000199080.1	acetyl-CoA acyltransferase	AID38845.1	-1.6	0.0009
WP_001250339.1	Mur ligase family protein	AID40431.1	-1.6	0.0000
WP_001068529.1	aminotransferase class I/II-fold pyridoxal phosphate-dependent enzyme	AID40480.1	-1.6	0.0009
WP_000181322.1	6-phosphogluconolactonase	AID40465.1	-1.6	0.0000
WP_000257888.1	DUF1447 family protein	AID39527.1	-1.6	0.0000
WP_000011827.1	Putative N-acetyltransferase YedL/Putative exported protein	AID39783.1	-1.6	0.0000
WP_000426914.1	acyl carrier protein	acpP	-1.6	0.0771
WP_000047355.1	malonyl CoA-ACP transacylase	fabD	-1.6	0.0000
WP_001050913.1	ribonuclease Y	rny	-1.6	0.0000
WP_038413163.1	collagen adhesin	AID41408.1	-1.6	0.0013
WP_000367420.1	BrxA/BrxB family bacilliredoxin	AID39979.1	-1.6	0.0000
WP_000411087.1	D-alanine aminotransferase	dat	-1.6	0.0025
WP_000751265.1	transglycosylase IsaA	isaA	-1.6	0.0000
WP_000866426.1	glucosamine-6-phosphate deaminase	nagB	-1.6	0.0009
WP_000969811.1	DNA polymerase III subunit beta	dnaN	-1.6	0.0000
WP_000004981.1	Adenosine 5'-monophosphoramidase	AID40308.1	-1.7	0.0000
WP_001138360.1	50S ribosomal protein L20	rplT	-1.7	0.0063
WP_000677261.1	FMN-dependent NADPH-azoreductase	azo1	-1.7	0.0000
WP_001059082.1	cold-shock protein CspC	AID39302.1	-1.7	0.0000
WP_000134779.1	DUF1292 domain-containing protein	AID40084.1	-1.7	0.0009
WP_000818278.1	succinate dehydrogenase flavoprotein subunit	sdhA	-1.7	0.0000
WP_000134963.1	thioredoxin reductase TrxB	trxB	-1.7	0.0000
WP_000277155.1	hypothetical multicopper oxidase protein	#N/A	-1.7	0.0016
WP_000623906.1	DAK2 domain-containing protein	AID39667.1	-1.7	0.0026
WP_000448898.1	alpha/beta hydrolase	AID41272.1	-1.7	0.0009
WP_000532966.1	YebC/PmpR family DNA-binding transcriptional regulator	AID39177.1	-1.7	0.0084
WP_000138342.1	penicillin-binding protein 2	pbp2	-1.8	0.0084
WP_000611456.1	UDP-N-acetylmuramoyl-tripeptide--D-alanyl-D-alanine ligase	murF	-1.8	0.0013
WP_001041575.1	heme uptake protein IsdB	isdB	-1.8	0.0013
WP_000884337.1	serine--tRNA ligase	serS	-1.8	0.0079
WP_000673098.1	glycine glycyltransferase FemB	femB	-1.8	0.0016
WP_000129645.1	D-alanine--poly(phosphoribitol) ligase	dltA	-1.8	0.0000
WP_001055897.1	XRE family transcriptional regulator	AID38865.1	-1.8	0.0009
WP_001792529.1	LytR-Cps2A-Psr (LCP) family transcriptional regulator	AID40999.1	-1.8	0.0006
WP_000058389.1	NAD-dependent malic enzyme 4	AID40171.1	-1.8	0.0009
WP_001018928.1	thioredoxin TrxA	trxA	-1.8	0.0000
WP_000547687.1	50S ribosomal protein L24	rplX	-1.8	0.0192
WP_000631982.1	cysteine--tRNA ligase	cysS	-1.8	0.0000
WP_001125210.1	2,3-bisphosphoglycerate-dependent phosphoglycerate mutase	gpmA	-1.8	0.0000
WP_000197806.1	phosphopentomutase	deoB	-1.8	0.0016
WP_000551283.1	hypoxanthine-guanine phosphoribosyltransferase	hpt	-1.8	0.0000
WP_001286320.1	cell division regulator GpsB	gpsB	-1.9	0.0000
WP_001085498.1	2,3-bisphosphoglycerate-independent phosphoglycerate mutase	gpmI	-1.9	0.0000
WP_000786119.1	YhgE/Pip domain-containing protein	AID41355.1	-1.9	0.0000
WP_000160304.1	purine-nucleoside phosphorylase	AID40824.1	-1.9	0.0000
WP_000201875.1	lipoyl synthase	AID39361.1	-1.9	0.0000
WP_000569649.1	DNA-directed RNA polymerase subunit alpha	rpoA	-1.9	0.0400
WP_000290401.1	aldehyde dehydrogenase	aldA	-1.9	0.0400
WP_000334506.1	VOC family protein	AID39871.1	-1.9	0.0000
WP_000172190.1	hydroxymethylglutaryl-CoA synthase	mvaS	-1.9	0.0078
WP_000120494.1	serine hydroxymethyltransferase	glyA	-2.0	0.0000
WP_000649898.1	glutamate ABC transporter permease	AID40328.1	-2.0	0.0000
WP_001801513.1	anti-sigma B factor RsbW	rsbW	-2.0	0.0009
WP_000035058.1	peptidyl-prolyl cis-trans isomerase	AID39390.1	-2.0	0.0000
WP_001031407.1	class I fructose-bisphosphate aldolase	fda	-2.0	0.0000
WP_001093552.1	hypothetical membrane lipoprotein	AID39217.1	-2.0	0.0000
WP_000066056.1	argininosuccinate lyase	argH	-2.0	0.0005
WP_001557601.1	HIRAN-domain-containing restriction endonuclease	AID38752.1	-2.0	0.0009
WP_000819098.1	DNA gyrase subunit A	gyrA	-2.0	0.0000
WP_001283055.1	RNA polymerase sigma factor SigA	rpoD	-2.0	0.0026
WP_000159960.1	CTP synthetase	pyrG	-2.0	0.0000
WP_000170162.1	Asp-tRNA(Asn)/Glu-tRNA(Gln) amidotransferase GatCAB subunit C	gatC	-2.0	0.0000
WP_000782130.1	foldase protein PrsA	prsA	-2.0	0.0016
WP_000825817.1	alkaline shock response membrane anchor protein AmaP	AID40877.1	-2.0	0.0009
WP_000589549.1	arsenate reductase family protein	AID39316.1	-2.0	0.0000
WP_001266540.1	PTS fructose transporter subunit IIC	fruA	-2.0	0.0000
WP_000863474.1	signal recognition particle protein	ffh	-2.0	0.0000
WP_000669728.1	protein MAP	AID40482.1	-2.1	0.0000
WP_000976253.1	dihydroxyacetone kinase subunit DhaK	AID39158.1	-2.1	0.0000

Entry name	Protein function	STRING ID	Log_2 ΔLFQ	q value
WP_000277421.1	dihydroorotate dehydrogenase (quinone)	AID41301.1	-2.1	0.0000
WP_000241346.1	ATP synthase subunit delta	atpH	-2.1	0.0025
WP_000279414.1	Glyceraldehyde-3-phosphate dehydrogenase 1	gapA1	-2.1	0.0000
WP_000913317.1	peptide-methionine (R)-S-oxide reductase	msrB	-2.1	0.0000
WP_000496092.1	GTPase ObgE	obg	-2.1	0.0000
WP_001791219.1	translation initiation factor IF-3	infC	-2.1	0.0000
WP_001155271.1	formate dehydrogenase subunit alpha	fdhF	-2.1	0.0000
WP_000067112.1	N-acetyltransferase	AID41211.1	-2.1	0.0000
WP_000413875.1	lipid II:glycine glycyltransferase FemX	femX	-2.1	0.0000
WP_000521495.1	phosphoglucosamine mutase	glmM	-2.1	0.0000
WP_000192183.1	fructose 1,6-bisphosphatase	fbp	-2.1	0.0000
WP_001280006.1	ribosome-recycling factor	frr	-2.1	0.0000
WP_000138487.1	NAD-specific glutamate dehydrogenase	gluD	-2.1	0.0000
WP_000974881.1	ATP synthase subunit alpha	atpA	-2.1	0.0000
WP_000259692.1	L-glutamate gamma-semialdehyde dehydrogenase	rocA	-2.1	0.0000
WP_000481438.1	transketolase	tkt	-2.1	0.0000
WP_001213992.1	YbaB/EbfC family nucleoid-associated protein	AID38970.1	-2.1	0.0035
WP_000867193.1	dihydroxyacetone kinase subunit L	AID39159.1	-2.2	0.0000
WP_001140871.1	manganese-dependent inorganic pyrophosphatase	ppaC	-2.2	0.0000
WP_000334466.1	glutamine--fructose-6-phosphate aminotransferase	glmS	-2.2	0.0000
WP_000911657.1	pyruvate formate-lyase-activating enzyme	pflA	-2.2	0.0000
WP_001240826.1	ESAT-6 secretion system extracellular protein A	esxA	-2.2	0.0063
WP_000093347.1	aminomethyltransferase	gcvT	-2.2	0.0000
WP_000382892.1	lantibiotic ABC transporter ATP-binding protein	AID41204.1	-2.2	0.0000
WP_000157603.1	ATP synthase subunit gamma	atpG	-2.2	0.0000
WP_001283784.1	glutamate--tRNA ligase	gltX	-2.2	0.0000
WP_000608835.1	iron-sulfur cluster repair di-iron protein ScdA	scdA	-2.2	0.0000
WP_000034716.1	molecular chaperone DnaK	dnaK	-2.2	0.0000
WP_000431312.1	transcription elongation factor GreA	greA	-2.2	0.0000
WP_000169223.1	6-phospho-beta-galactosidase	lacG	-2.2	0.0016
WP_000958522.1	chromosome partitioning protein ParA	AID40854.1	-2.2	0.0000
WP_001279341.1	PDZ domain-containing protein	AID40197.1	-2.2	0.0000
WP_000101976.1	DNA-binding response regulator	walR	-2.2	0.0000
WP_000626504.1	elongation factor P	efp	-2.2	0.0000
WP_000379051.1	HslU--HslV peptidase ATPase subunit	hslU	-2.3	0.0000
WP_000734077.1	alanine--tRNA ligase	alaS	-2.3	0.0000
WP_000813536.1	pyruvate decarboxylase	AID38614.1	-2.3	0.0017
WP_001261460.1	30S ribosomal protein S6	rpsF	-2.3	0.0000
WP_001228158.1	alpha/beta hydrolase	AID41225.1	-2.3	0.0000
WP_000219068.1	catabolite control protein A	ccpA	-2.3	0.0000
WP_000729731.1	aconitate hydratase	acnA	-2.3	0.0000
WP_000250820.1	2-amino-3-ketobutyrate CoA ligase	AID39045.1	-2.3	0.0000
WP_000205576.1	Fe-S cluster assembly protein SufD	AID39351.1	-2.3	0.0000
WP_000872486.1	2-C-methyl-D-erythritol 4-phosphate cytidylyltransferase	tarI	-2.3	0.0000
WP_000163281.1	type 1 glutamine amidotransferase	AID40346.1	-2.4	0.0000
WP_000160914.1	undecaprenyldiphospho-muramoylpentapeptide beta-N-acetylglucosaminyltransferase	murG	-2.4	0.0000
WP_001051856.1	YtxH domain-containing protein	AID40224.1	-2.4	0.0000
WP_000265408.1	dipeptidase PepV	AID40223.1	-2.4	0.0000
WP_000545373.1	Asp-tRNA(Asn)/Glu-tRNA(Gln) amidotransferase GatCAB subunit B	gatB	-2.4	0.0016
WP_000019697.1	aminomethyl-transferring glycine dehydrogenase	gcvPA	-2.4	0.0019
WP_000167269.1	3-oxoacyl-[acyl-carrier-protein] reductase	fabG	-2.4	0.0000
WP_000782741.1	molybdate ABC transporter substrate-binding protein	modA	-2.4	0.0000
WP_001011603.1	glutamate-1-semialdehyde 2,1-aminomutase	hemL2	-2.4	0.0006
WP_001274017.1	30S ribosomal protein S20	rpsT	-2.4	0.0000
WP_000182215.1	nucleotide exchange factor GrpE	grpE	-2.4	0.0000
WP_001051120.1	methionine--tRNA ligase	metG	-2.5	0.0000
WP_000184947.1	1,4-dihydroxy-2-naphthoyl-CoA synthase	menB	-2.5	0.0000
WP_000668822.1	lipoate--protein ligase A	AID39465.1	-2.5	0.0000
WP_000277602.1	universal stress protein	AID40175.1	-2.5	0.0097
WP_001062173.1	DEAD/DEAH box family ATP-dependent RNA helicase CshB	cshB	-2.5	0.0000
WP_000717560.1	ATP-dependent 6-phosphofructokinase	pfkA	-2.5	0.0000
WP_000933774.1	ribose-phosphate pyrophosphokinase	prs	-2.5	0.0009
WP_001269643.1	pur operon repressor	purR	-2.6	0.0000
WP_000154506.1	3-hydroxyacyl-CoA dehydrogenase	AID38649.1	-2.6	0.0000
WP_001096494.1	transaldolase	AID40252.1	-2.6	0.0000
WP_001062662.1	pyruvate oxidase CidC	AID41229.1	-2.6	0.0000
WP_000689997.1	alanine dehydrogenase	ald2	-2.6	0.0000
WP_000425358.1	valine--tRNA ligase	valS	-2.6	0.0000
WP_000395692.1	D-alanine--poly(phosphoribitol) ligase subunit 2	dltC	-2.6	0.0000
WP_000916187.1	50S ribosomal protein L27	rpmA	-2.6	0.0026
WP_000825937.1	amino acid ABC transporter substrate-binding protein	AID41110.1	-2.6	0.0000
WP_001038301.1	DNA polymerase I	polA	-2.6	0.0000
WP_000140675.1	ATP synthase subunit B	atpF	-2.6	0.0000
WP_000082722.1	oligoendopeptidase F	AID39438.1	-2.6	0.0000
WP_000422864.1	3-hexulose-6-phosphate synthase	AID39068.1	-2.6	0.0000
WP_000166055.1	fatty acid-binding protein DegV	AID39887.1	-2.7	0.0000
WP_001226833.1	urocanate hydratase	hutU	-2.7	0.0000
WP_001791353.1	putative heat induced stress protein	AID38877.1	-2.7	0.0000
WP_000004086.1	30S ribosomal protein S17	rpsQ	-2.7	0.0000
WP_000562498.1	class 1b ribonucleoside-diphosphate reductase subunit beta	nrdF	-2.7	0.0000
WP_000290491.1	glycine cleavage system protein H	gcvH	-2.7	0.0000

Entry name	Protein function	STRING ID	Log_2 ΔLFQ	q value
WP_001196351.1	proline dehydrogenase	AID40238.1	-2.7	0.0000
WP_000584639.1	DNA double-strand break repair Rad50 ATPase	AID40313.1	-2.8	0.0000
WP_000193707.1	phosphogluconate dehydrogenase (NADP(+)-dependent, decarboxylating)	gnd	-2.8	0.0000
WP_000244860.1	septation ring formation regulator EzrA	ezrA	-2.8	0.0000
WP_000081181.1	beta-ketoacyl-[acyl-carrier-protein] synthase II	fabF	-2.8	0.0000
WP_000186043.1	tyrosine--tRNA ligase	tyrS	-2.8	0.0000
WP_000930503.1	alkyl hydroperoxide reductase subunit F	ahpF	-2.8	0.0000
WP_000791398.1	uncharacterized leukocidin-like protein 2	AID40551.1	-2.8	0.0000
WP_000076025.1	branched chain amino acid aminotransferase	ilvE	-2.8	0.0000
WP_000159638.1	D-alanine--D-alanine ligase	ddl	-2.9	0.0000
WP_000491755.1	DUF47 domain-containing protein	AID39171.1	-2.9	0.0000
WP_000109909.1	phosphoenolpyruvate carboxykinase (ATP)	pckA	-2.9	0.0009
WP_077670284.1	MSCRAMM family adhesin SdrC	sdrC	-2.9	0.0017
WP_001232655.1	pyruvate kinase	pyk	-2.9	0.0000
WP_000942303.1	ABC transporter ATP-binding protein	AID39848.1	-2.9	0.0000
WP_000985472.1	50S ribosomal protein L2	rplB	-2.9	0.0381
WP_000435132.1	threonine--tRNA ligase	thrS	-2.9	0.0000
WP_001283444.1	MarR family transcriptional regulator	rat	-3.0	0.0000
WP_000473654.1	glucose-specific phosphotransferase enzyme IIA component	crr	-3.0	0.0000
WP_000043642.1	translation initiation factor IF-2	infB	-3.0	0.0000
WP_000814087.1	proline--tRNA ligase	proS	-3.0	0.0017
WP_000057330.1	UMP kinase	pyrH	-3.0	0.0000
WP_001108721.1	leucine--tRNA ligase	leuS	-3.0	0.0000
WP_000202178.1	glycine dehydrogenase	gcvPB	-3.0	0.0000
WP_001115101.1	transcription termination factor Rho	rho	-3.1	0.0000
WP_000090796.1	30S ribosomal protein S13	rpsM	-3.1	0.0000
WP_000160848.1	iron-regulated surface determinant protein A (IsdA)	isdA	-3.1	0.0000
WP_000046076.1	NADH dehydrogenase	AID39377.1	-3.1	0.0000
WP_000027924.1	Asp-tRNA(Asn)/Glu-tRNA(Gln) amidotransferase GatCAB subunit A	gatA	-3.1	0.0026
WP_000955797.1	bifunctional acetaldehyde-CoA/alcohol dehydrogenase	adhE	-3.1	0.0000
WP_000182648.1	translational GTPase TypA	AID39546.1	-3.1	0.0000
WP_001273085.1	50S ribosomal protein L10	rplJ	-3.1	0.0026
WP_000048712.1	uracil phosphoribosyltransferase	upp	-3.1	0.0000
WP_000180655.1	2-oxoglutarate dehydrogenase E1 component	odhA	-3.2	0.0000
WP_000472293.1	ATP-dependent Clp protease ATP-binding subunit ClpX	clpX	-3.2	0.0000
WP_000745926.1	clumping factor B (ClfB)	clfB	-3.2	0.0027
WP_000358009.1	UDP-N-acetylglucosamine 1-carboxyvinyltransferase	murA1	-3.2	0.0017
WP_001218603.1	aerobic glycerol-3-phosphate dehydrogenase	glpD	-3.2	0.0027
WP_000168845.1	Fe-S cluster assembly ATPase SufC	AID39350.1	-3.2	0.0000
WP_000040043.1	phosphoenolpyruvate--protein phosphotransferase	ptsI	-3.3	0.0000
WP_001063330.1	ATP-dependent Clp protease ATP-binding subunit	clpL	-3.3	0.0016
WP_001167888.1	ATP-dependent metallopeptidase FtsH/Yme1/Tma family protein	ftsH	-3.3	0.0000
WP_000417008.1	2-hydroxyacid dehydrogenase	AID40993.1	-3.3	0.0000
WP_001049165.1	ATP-dependent Clp protease proteolytic subunit ClpP (subunit specified manually)	clpP	-3.3	0.0000
WP_001251219.1	glucosamine-6-phosphate isomerase	AID40324.1	-3.4	0.0000
WP_000368166.1	DNA recombination/repair protein RecA	recA	-3.4	0.0000
WP_000024830.1	50S ribosomal protein L4	rplD	-3.5	0.0000
WP_001288196.1	lysine--tRNA ligase	lysS	-3.5	0.0000
WP_000727325.1	DUF4889 domain-containing protein	AID41078.1	-3.5	0.0000
WP_001270861.1	glutamate-1-semialdehyde 2,1-aminomutase	hemL1	-3.5	0.0000
WP_000055337.1	GTP-sensing pleiotropic transcriptional regulator CodY	codY	-3.5	0.0000
WP_000268754.1	30S ribosomal protein S16	rpsP	-3.6	0.0105
WP_000225845.1	bifunctional methylenetetrahydrofolate dehydrogenase/methenyltetrahydrofolate cyclohydrolase	folD	-3.6	0.0000
WP_000926310.1	50S ribosomal protein L16	rplP	-3.6	0.0000
WP_001031880.1	L-lactate dehydrogenase	ldh1	-3.6	0.0000
WP_001020801.1	succinyl-CoA ligase subunit beta	sucC	-3.6	0.0000
WP_000506536.1	protein translocase subunit SecA 1	secA1	-3.6	0.0000
WP_001096577.1	50S ribosomal protein L30	rpmD	-3.7	0.0000
WP_001126603.1	glutamine synthetase	glnA	-3.7	0.0000
WP_000149412.1	formate--tetrahydrofolate ligase	fhs	-3.7	0.0000
WP_000178881.1	30S ribosomal protein S8	rpsH	-3.7	0.0000
WP_001018328.1	30S ribosomal protein S15	rpsO	-3.7	0.0000
WP_000160212.1	50S ribosomal protein L3	rplC	-3.7	0.0000
WP_000542274.1	50S ribosomal protein L17	rplQ	-3.7	0.0000
WP_000954802.1	nitric oxide dioxygenase	AID38659.1	-3.7	0.0000
WP_001074521.1	bifunctional autolysin Atl	atl	-3.8	0.0019
WP_000766074.1	50S ribosomal protein L15	rplO	-3.9	0.0000
WP_000918664.1	DNA-directed RNA polymerase subunit beta	rpoB	-3.9	0.0000
WP_000388082.1	50S ribosomal protein L23	rplW	-3.9	0.0000
WP_001030080.1	glycine--tRNA ligase	glyQS	-3.9	0.0000
WP_001790547.1	30S ribosomal protein S9	rpsI	-3.9	0.0000
WP_000644737.1	50S ribosomal protein L29	rpmC	-4.0	0.0000
WP_000424963.1	GMP synthase (glutamine-hydrolyzing)	guaA	-4.0	0.0000
WP_000383814.1	DUF948 domain-containing protein	AID40210.1	-4.1	0.0000
WP_000855505.1	class 1b ribonucleoside-diphosphate reductase subunit alpha	nrdE	-4.1	0.0000
WP_000167322.1	ornithine--oxo-acid transaminase	AID39393.1	-4.1	0.0000
WP_000034728.1	pyridoxal 5'-phosphate synthase lyase subunit PdxS	pdxS	-4.1	0.0000
WP_000124353.1	30S ribosomal protein S19	rpsS	-4.2	0.0000

Entry name	Protein function	STRING ID	Log_2 ΔLFQ	q value
WP_000090514.1	30S ribosomal protein S4	rpsD	-4.2	0.0000
WP_001260089.1	triose-phosphate isomerase	tpiA	-4.2	0.0000
WP_000511135.1	ATP synthase subunit beta	atpD	-4.2	0.0017
WP_001085792.1	50S ribosomal protein L11	rplK	-4.2	0.0000
WP_000437472.1	phosphocarrier protein HPr	AID39520.1	-4.3	0.0000
WP_000101625.1	30S ribosomal protein S11	rpsK	-4.3	0.0000
WP_001074353.1	carbamate kinase 1	arcC1	-4.3	0.0000
WP_001178940.1	DEAD/DEAH box family ATP-dependent RNA helicase	cshA	-4.4	0.0000
WP_077670278.1	clumping factor A (ClfA)	clfA	-4.4	0.0000
WP_000181404.1	50S ribosomal protein L19	rplS	-4.4	0.0000
WP_000774286.1	phosphate acetyltransferase	pta	-4.5	0.0000
WP_000660054.1	argininosuccinate synthase	argG	-4.5	0.0000
WP_000387527.1	50S ribosomal protein L22	rplV	-4.6	0.0000
WP_000157650.1	50S ribosomal protein L25	ctc	-4.7	0.0000
WP_000136159.1	ornithine carbamoyltransferase ArcB	arcB	-4.7	0.0000
WP_000068176.1	pyruvate dehydrogenase E1 component subunit beta	pdhB	-4.7	0.0026
WP_000617735.1	ribosomal subunit interface protein	hpf	-4.8	0.0000
WP_001074749.1	phosphoglycerate kinase	pgk	-4.8	0.0000
WP_000442480.1	nucleoside-diphosphate kinase	ndk	-5.0	0.0000
WP_000959426.1	alanine dehydrogenase	ald1	-5.1	0.0000
WP_000825534.1	methionine ABC transporter substrate-binding protein (aka NLPA lipoprotein)	AID38955.1	-5.6	0.0173
WP_000210828.1	serine/threonine dehydratase	tdcB	-5.6	0.0000
WP_001043863.1	DNA-binding protein HU	AID39935.1	-6.3	0.0000
WP_001242307.1	pyrimidine-nucleoside phosphorylase	pdp	-6.3	0.0000
WP_000634175.1	universal stress protein UspA	AID40179.1	-6.4	0.0000
WP_001131841.1	fructose-bisphosphate aldolase	fba	-6.7	0.0000

References

Almagro Armenteros, J.J., Tsirigos, K.D., Sønderby, C.K., Petersen, T.N., Winther, O., Brunak, S., Von Heijne, G., and Nielsen, H. (2019). SignalP 5.0 improves signal peptide predictions using deep neural networks. *Nat Biotechnol* 37**,** 420-423.

Bendtsen, J.D., Kiemer, L., Fausbøll, A., and Brunak, S. (2005). Non-classical protein secretion in bacteria. *BMC Microbiol* 5**,** 58.

Brohée, S., and Van Helden, J. (2006). Evaluation of clustering algorithms for protein-protein interaction networks. *BMC Bioinformatics* 7**,** 488.

Szklarczyk, D., Gable, A.L., Lyon, D., Junge, A., Wyder, S., Huerta-Cepas, J., Simonovic, M., Doncheva, N.T., Morris, J.H., Bork, P., Jensen, L.J., and Mering, C.V. (2019). STRING v11: protein-protein association networks with increased coverage, supporting functional discovery in genome-wide experimental datasets. *Nucleic Acids Res* 47**,** D607-d613.
